# Generative Deep Learning Pipeline Yields Potent Gram-Negative Antibiotics

**DOI:** 10.1021/jacsau.5c00602

**Published:** 2025-09-09

**Authors:** Martin F. Köllen, Maximilian G. Schuh, Robin Kretschmer, Joshua Hesse, Dominik Schum, Junhong Chen, Annkathrin I. Bohne, Dominik P. Halter, Stephan A. Sieber

**Affiliations:** † TUM School of Natural Sciences, Department of Bioscience, Center for Functional Protein Assemblies (CPA), Chair of Organic Chemistry II, 9184 Technical University of Munich, 85748 Garching bei München, Germany; ‡ TUM School of Natural Sciences, Department of Chemistry, Catalysis Research Center (CRC), Chair of Inorganic and Metal−Organic Chemistry, Technical University of Munich, 85748 Garching bei München, Germany; § TUM School of Natural Sciences, Department of Bioscience, Center for Functional Protein Assemblies (CPA), Chair of Biochemistry, Technical University of Munich, 85748 Garching bei München, Germany; ∥ Faculty of Applied Engineering, Department of Biochemical and Chemical Engineering, Research Group Applied Electrochemistry & Catalysis (ELCAT), University of Antwerp, 2610 Antwerp, Belgium

**Keywords:** deep learning, machine learning, drug discovery, antibiotics, Gram-negative, MRSA, de novo drug design, automated synthesis

## Abstract

The escalating crisis of multiresistant bacteria demands the rapid discovery of novel antibiotics that transcend the limitations imposed by the biased chemical space of current libraries. To address this challenge, we introduce an innovative deep learning-driven pipeline for *de novo* antibiotic design. Our unique approach leverages a chemical language model to generate structurally unprecedented antibiotic candidates. The model was trained on a diverse chemical space of drug-like molecules and natural products. We then applied transfer learning using a data set of diverse antibiotic scaffolds to refine its generative capabilities. Using predictive modeling and expert curation, we prioritized the most promising compounds for synthesis. This pipeline identified a lead candidate with potent activity against methicillin-resistant *Staphylococcus aureus*. We then performed iterative refinement by synthesizing 40 derivatives of the lead compound. This effort produced a suite of active compounds, with 30 showing activity against *S. aureus* and 17 against *Escherichia coli*. Among these, lead compound **D8** exhibited remarkable submicromolar and single-digit micromolar potency against the aforementioned pathogens, respectively. Mechanistic investigations point to the reductive generation of reactive species as its primary mode of action. This work validates a deep-learning pipeline that explores chemical space to generate antibiotic candidates. This process yields a potent nitrofuran derivative and a set of experimentally validated scaffolds to seed future antibiotic development.

## Graphical abstract

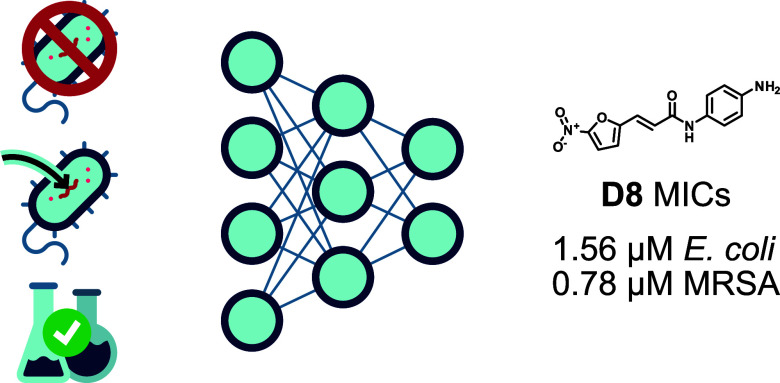

## Introduction

In recent decades, multidrug-resistant bacteria have spread at an alarming rate, while the discovery of new antibiotics has slowed significantly.^1^ As a result, scientists and the World Health Organization (WHO) warn of an impending “post-antibiotic era”.^2–4^ Of particular concern are Gram-negative strains such as uropathogenic *Escherichia coli*, which have a double membrane that is almost impenetrable to antibiotic molecules. One major challenge in antibiotic discovery is that existing public, commercial, and proprietary molecular libraries occupy a chemical space that differs from what is empirically needed.^4–6^ To overcome limitations such as antibiotic target space as well as uptake into bacteria, this study explores uncharted chemical space by generating *de novo* molecules. However, navigating such an immense chemical space—estimated to contain up to 10^60^ possible molecules—poses a significant challenge, as traditional high-throughput screening and trial-and-error synthesis are both time-consuming and costly.^7,8^ Recent developments in deep learning (DL) have introduced generative models that can design molecules *de novo* by learning patterns that underlie chemical structures, thereby exploring the densely populated chemical space.^9–13^ Among these methods, chemical language models (CLMs) trained on molecules as strings (e.g., simplified molecular-input line-entry system (SMILES) notation) have proven effective in generating novel and viable compounds.^11,12,14–16^ By leveraging techniques such as transfer learning, these models can be adapted to specific targets even when available data is limited. We present a framework that combines multiple state-of-the-art machine learning (ML) and DL strategies into a streamlined generation and curation process for *de novo* antibiotic design. Our approach enables rapid generation of custom virtual libraries, offering a practical solution to explore chemical space efficiently and to accelerate early stage drug discovery.

We employ a CLM trained on data from three different chemical domains to maximize the chance to identify potent and novel chemical molecules.^11^ First, bioactive compounds from ChEMBL were incorporated to familiarize the model with drug-like molecules.^17^ Second, the natural product space was included, given its historical significance, e.g., in antibiotic drug discovery, with approximately 60–80% of all antibiotic and antitumor drugs deriving or inspired from natural products.^18–20^ Finally, we introduced a structurally diverse set of antibiotic molecules to avoid over-representing well-researched scaffolds and to prioritize diverse chemical structures.^21^ Our study includes the synthesis of the selected compounds, performing a structure–activity relationship (SAR) study, and evaluating their antibiotic activity and human toxicity. We proceeded in the following steps:

1. ***De novo*scaffold generation.** We applied transfer learning of known antibiotics to a CLM (Figure 1a).^11^ To minimize bias toward well-explored scaffolds, we selected 131 structurally diverse molecules by reducing the Tanimoto similarity among them.
2. **Scaffold prioritization.** We applied a curation pipeline to identify scaffolds that are both synthetically accessible and likely to exhibit antibiotic activity. First, we assessed synthetic feasibility by DL methods.^22,23^ Next, we performed a weighted ensemble prediction of whether the *de novo* molecules accumulate in Gram-negative bacteria, applying automated machine learning (AutoML) and text-based zero-shot molecular property predictions (Figure 1a).^24–26^

3. **Synthesis and biological evaluation.** We performed retrosynthetic analyses for the most promising scaffolds and picked the most accessible ones. In the end, we successfully synthesized 11 candidates (Figure 1b). We tested their antibacterial activity against Gram-positive *Staphylococcus aureus*, but also challenging Gram-negative *E. coli* and *Pseudomonas aeruginosa* *.*
4. **Structure–activity relationship.** Employing an automated parallelized synthesis strategy, we created a set of 40 derivatives and tested them in a direct-to-biology (D2B) approach for their minimal inhibitory concentration (MIC) (Figure 1c).
5. **Hit evaluation and mode of action studies.** Lastly, we investigated the most potent compound for its efficacy against an ESKAPEE panel, as well as for its toxicity in human cells, and we performed assay- and mass spectrometry-based mode of action (MoA) studies.

**Figure 1:**
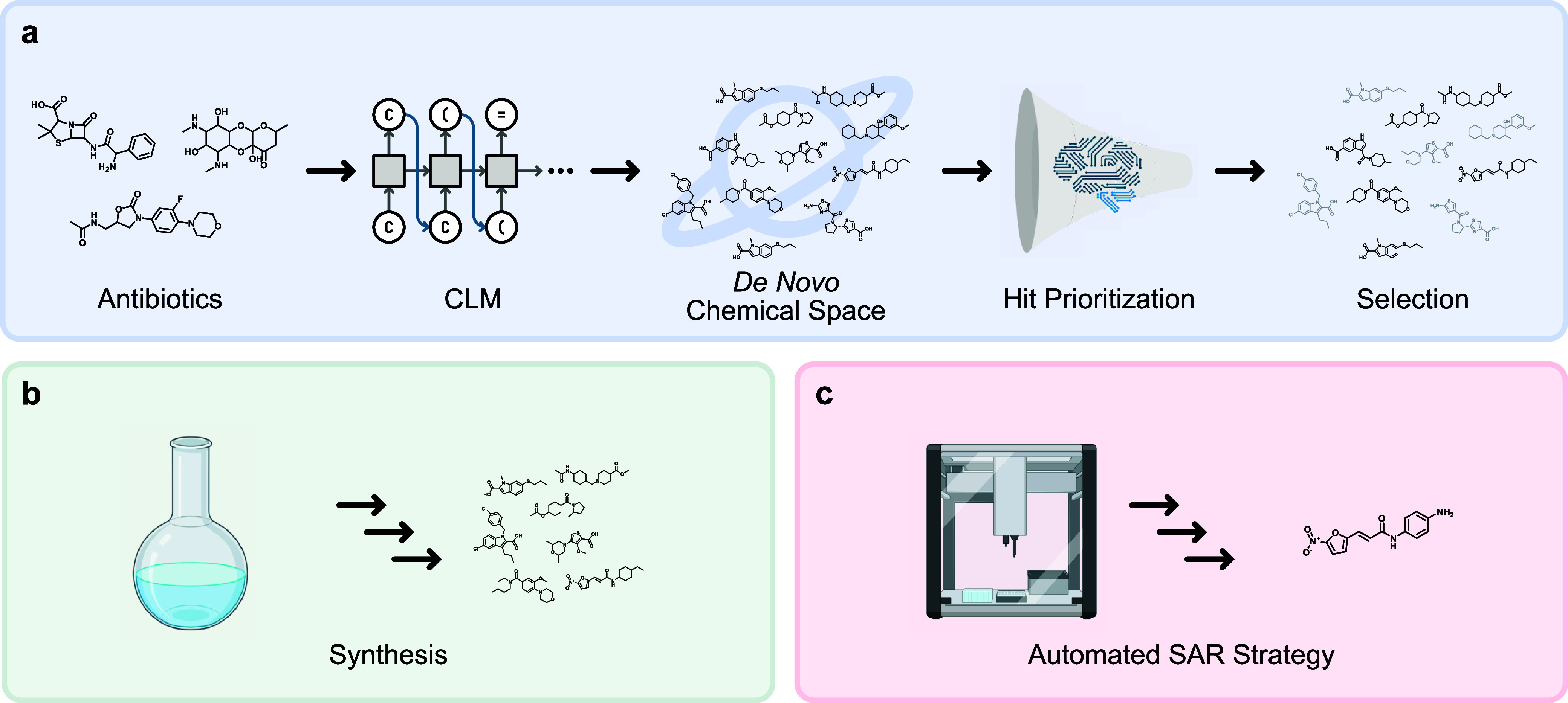
Pipeline overview. (a) We applied CLM-based transfer learning to generate new molecules trained on antibiotic data. To prioritize scaffolds for synthesis, we applied multiple ML and DL methods. (b) Synthetic routes toward the selected molecules were developed and the candidates were synthesized. (c) A robot-assisted SAR study was conducted to refine the hit molecule. Created with BioRender.com.

## Results and Discussion

### *De Novo* Design and Curation of Antibiotic-Like Scaffolds

#### Model Architecture

We applied transfer learning in two steps to generate *de novo* compounds from a CLM trained on drug-like molecules, natural products and antibiotic scaffold data. Specifically, this CLM was implemented as a recurrent neural network (RNN) with long short-term memory (LSTM) for SMILES-based chemical structure generation.^11,27^ For this process, each token of the SMILES string vocabulary is converted into a 71 bit one-hot encoded vector representation.^11^

#### Molecular Data

In detail, bioactive and standardized training data were compiled from ChEMBL 24 yielding ∼365,000 molecules in form of SMILES strings.^11,17^ Furthermore, molecules from the natural product space (MEGx collection, Analyticon Discovery GmbH), as well as antimicrobial scaffolds (PubChem database “antibiotic” subset) were collected for transfer learning steps. To reduce bias toward well-studied antibiotic scaffolds, we fine-tuned our model on a curated set of 131 structurally distinct “representative” antibiotic scaffolds selected from 2239 molecules retrieved from PubChem (Figures S2 and S5). Although the raw number of known antibiotics is large, the majority are close analogues derived from a limited number of core scaffolds. Previous studies have shown that nearly all approved antibiotics fall into fewer than 20–40 mechanistically distinct classes, comprising roughly 70 unique drug scaffolds.^28–31^ Including the full data set would therefore risk overrepresenting dominant scaffolds and reinforcing structural bias in the model. Our approach aligns with other generative design strategies that promote scaffold diversity and prevent structural redundancy.^32–34^ Furthermore, the selected method is optimized for low-data regimes.^11^

#### Transfer Learning

Finally, transfer learning was carried out for 40 epochs, in which a total of 3553 molecules were designed.^21^ Figure 2a shows the structural differences of molecule sets using a uniform manifold approximation and projection (UMAP) visualization, highlighting a clear distinction between the chemical spaces of bioactive molecules from ChEMBL and natural products. The antibiotics selected as one transfer learning set are distributed across both regions. We can observe the transfer learning of the CLM (Figure 2a) by looking at the shifts in the structural similarity of the generated scaffolds over the training epochs: at epoch 0, the molecules are aligned with the ChEMBL space, whereas at epoch 40, the *de novo* compounds predominantly resemble those in the transfer learning sets.

**Figure 2:**
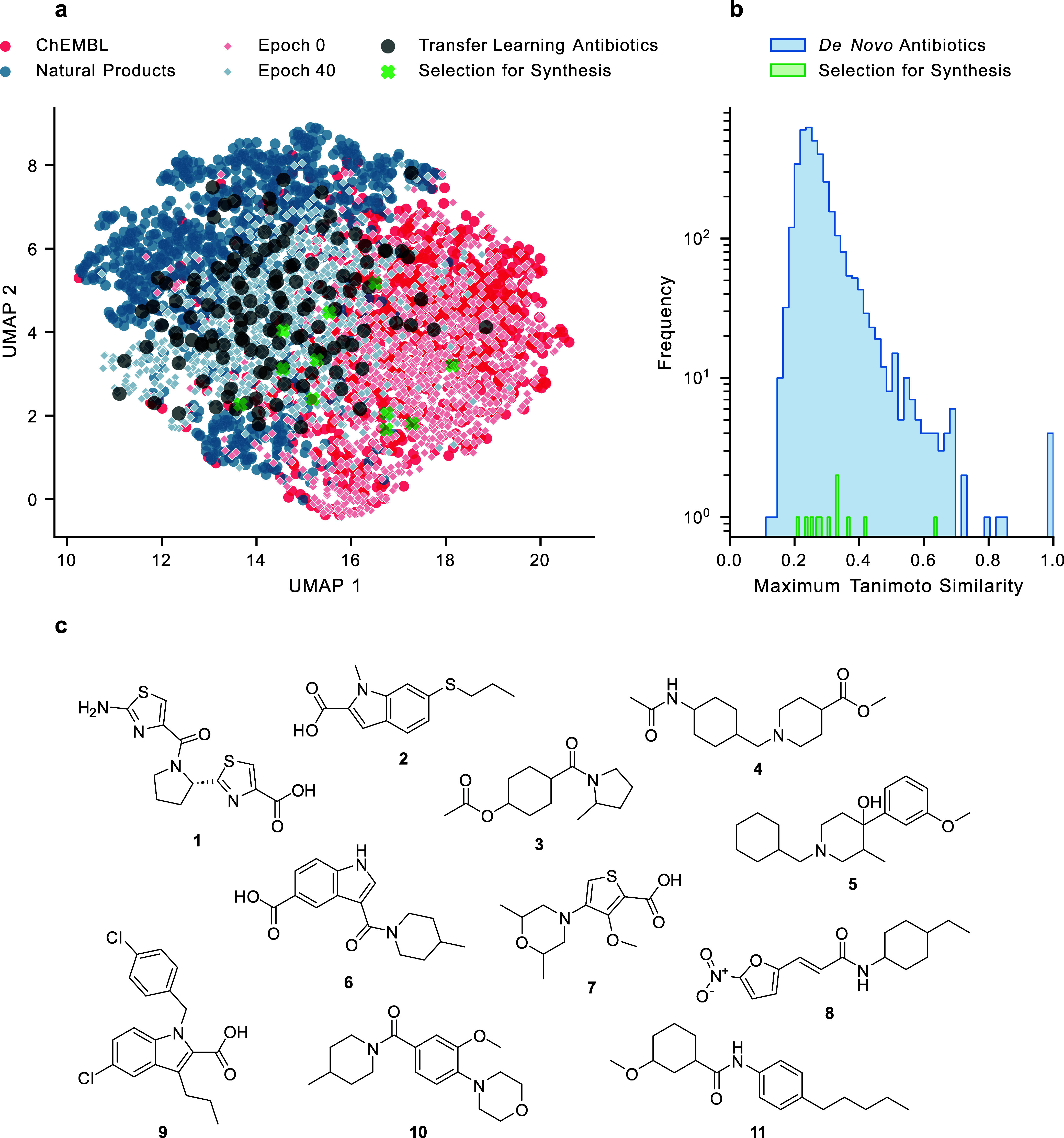
Molecule generation and selection. (a) UMAP plot of molecule sets (1000 molecules were randomly sampled for ChEMBL 24 and natural product (MEGx) groups). (b) We compared all generated molecules to 2239 existing antibiotics deposited in PubChem. The maximum Tanimoto similarity is presented. (c) These generated molecules were synthesized.

#### *De Novo* Curation

We implemented a curation pipeline to identify synthetically accessible scaffolds with a high likelihood of antibiotic activity. First, we assessed synthetic feasibility using the retrosynthetic accessibility score (RAscore).^22–26^ Second, to ensure effective accumulation in Gram-negative bacteria, we combined predictions from two distinct ML models. The first is an AutoML model trained on a known bacterial accumulation data set.^24,25^ The second is TwinBooster, a zero-shot prediction method that can handle novel and distant structures in chemical space.^26^ We calculated a final score using a weighted average of these outputs, where the weighting coefficient is the Tanimoto similarity (*s*) of a given molecule to the AutoML training set (mean top five similar molecules). This strategy dynamically prioritizes the AutoML prediction for molecules similar to the training data (*s* → 1) and the TwinBooster prediction for novel scaffolds (*s* → 0), ensuring robust accumulation predictions across diverse chemical space. The specific formula, along with experimental validation for this model, is provided in the Supporting Information (Figure S1 and sections “Additional Results and Discussion” and “Methods”).

Overall, our approach balanced synthetic accessibility with biological relevance, refining our selection of promising antibiotic scaffolds.

### From Prediction to Realization: Final Selection and Synthesis of the Candidates

All generated molecules were screened against the PubChem database, revealing only 177 known matches, emphasizing the ability of the model to generate novel structures. We further compared the generated molecules to existing antibiotic structures from PubChem (Figures 2b, S3 and S4), and observed a low Tanimoto similarity. To ensure novelty, we confirmed that all *de novo* scaffolds selected for synthesis are dissimilar to known antibiotics, with a maximum Tanimoto similarity well below 0.5 for 10 out of 11 molecules. Using the RAscore and the weighted accumulation ranking as an orientation for prioritization, the most promising molecules were inspected by a chemist for synthetic accessibility, as well as other parameters like hydrolytic, oxidative instability, probable insolubility or well-known functional group-mediated toxicity. The remaining candidates were subjected to detailed retrosynthetic analysis and the most accessible compounds were selected for synthesis. This led to the preparation of 11 distinct candidates shown in Figure 2c, which we tested for their antibacterial activity against three laboratory- and clinically relevant strains (*S. aureus* USA300, *E. coli* K12 and *P. aeruginosa* PAO1). The detailed synthetic routes are described in the Supporting Information (Figures S9–S19).

Satisfyingly, nitrofuran **8** exhibited an MIC of 6.3 μM in *S. aureus*, albeit no activity against the two Gram-negative bacteria. All other candidates showed no activity against any of the tested bacteria at up to 100 μM. Of note: the nitrofuran moiety lends its name to the known class of nitrofuran-antibiotics such as nitrofurantoin and nitrofurazone. However, all members of that class share a characteristic hydrazone moiety, while the alkylated acrylamide of **8** is a unique feature of this antibiotic. Thus, our approach demonstrated the ability of our model to recognize and adapt privileged structures to uncover unexplored chemical space.

### From Hit to Lead: Refining the Hit and Gain Insight into Its Antibacterial Profile

#### SAR Study

To evaluate the optimization potential of compound **8**, as well as its SAR, we prepared a library of derivatives. We hypothesized the nitrofuran part of the molecule to be essential for the activity and thus left it unchanged in our first panel of derivatives. Instead, we focused on the amide-half of the molecule for derivatization. We generated a set of molecules based on possible coupling products of 3-(5-nitro-2-furyl)­acrylic acid with 198 amines from our in house library. Subsequently, we used our AutoML model to predict and rank all derivatives based on their accumulation in *E. coli*.

The 48 derivatives with the highest accumulation score were selected for synthesis on a 0.1 mmol-scale in a robot-assisted parallelized synthesis panel, employing a *O*-(7-azabenzotriazol-1-yl)-1,1,3,3-tetramethyluronium hexafluorophosphate (HATU)-mediated amide coupling protocol, followed by precipitation of the products in water. After precipitation, 45 crude products were obtained and analyzed by high-performance liquid chromatography–mass spectrometry (HPLC–MS), confirming the successful synthesis of 40 derivatives, while 5 mixtures contained only unidentifiable species (see Table S1 and section “SAR study” of the Supporting Information). Following a direct-to-biology (D2B) approach, all crude products were tested directly for their activity in *S. aureus* USA300, *E. coli* K12 and *P. aeruginosa* PAO1 at fixed concentrations of 50, 100 and 200 μM (molar masses of pure products were assumed).

Of the 45 crude products tested, 18 showed activity in *S. aureus* at 50 μM or below. To our satisfaction, 17 products exhibited antibiotic effects on *E. coli*, which is a remarkable feature given its constrains in compound uptake. The most active compounds against *S. aureus* and *E. coli* (24 in total (**D1–D24**), see Table 1 and Table S2) were purified if necessary and subsequently tested for their MIC. Interestingly, distinct clusters of active and inactive structures were obtained for both *S. aureus* and *E. coli* in the UMAP (Figure 3).

**Table 1:**
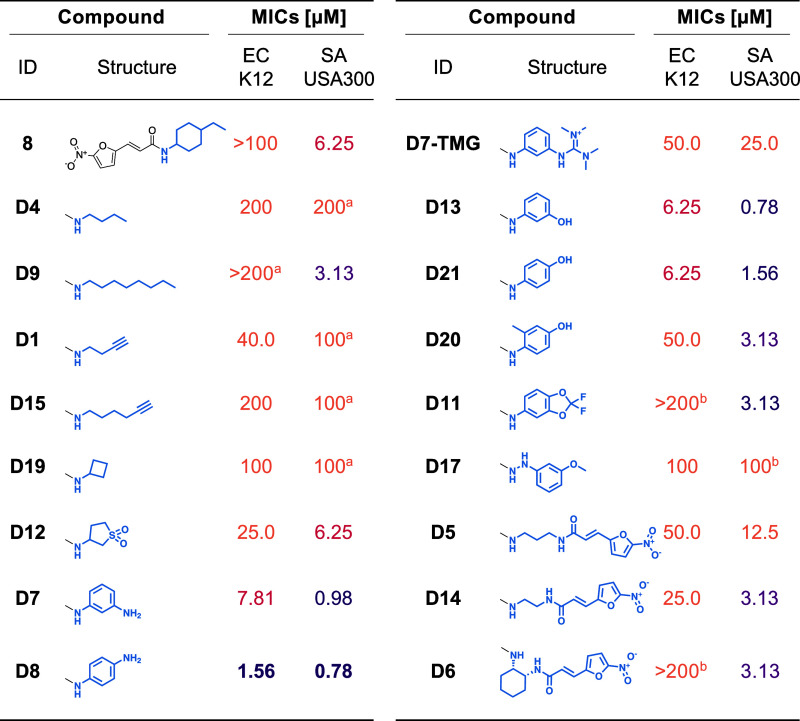
MICs and Structures of Selected Derivatives of Compound 8 in *E. coli* K12 (EC K12) and *S. aureus* USA300 (SA USA300**)** ^a^

a We grouped according to functional groups. Further, we color-coded the values, the darker the color, the lower the MIC. The number of the derivative is based on the predicted rank. (a) Determined under different conditions with a 100-fold inoculum. (b) Determined under different conditions before purification and with a 100-fold inoculum.

**Figure 3:**
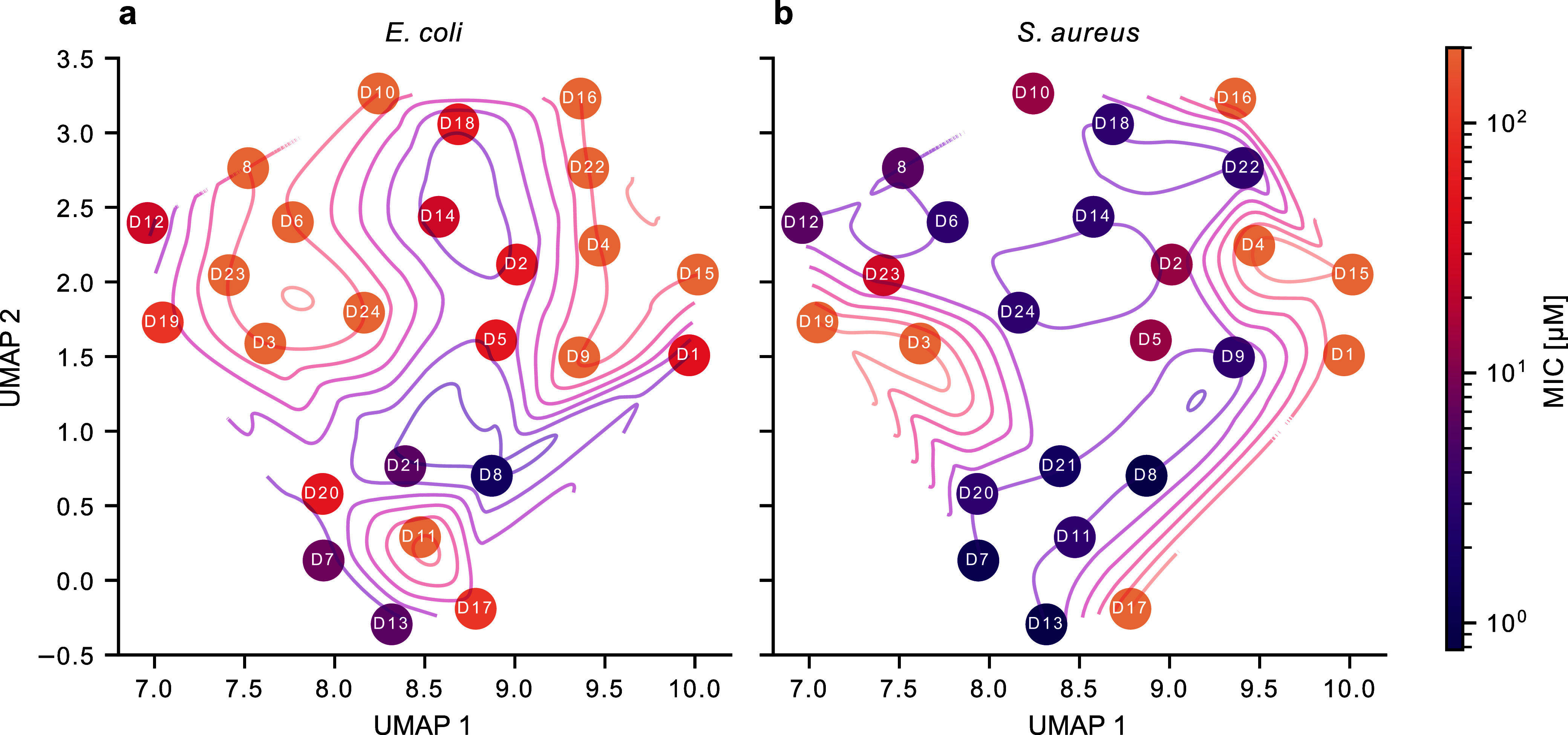
Structure–activity relationship visualization. Tested derivatives of **8** are represented as UMAP where structurally similar compounds are close and vice versa. They are color coded by MIC for both *E. coli* (a) and *S. aureus* (b). The darker the color, the lower the MIC.

One cluster, comprising compounds **D7**, **D8**, **D11**, **D13**, **D17**, **D20** and **D21**, is of particular interest, as it bears the most potent derivatives for both bacteria (**D8**: 0.78 μM and 1.56 μM, **D13**: 0.78 μM and 6.25 μM, **D7**: 0.98 μM and 7.81 μM, **D21**: 1.56 μM and 6.25 μM in *S. aureus* and *E. coli*, respectively), except for **D11** (3.13 μM and >200 μM) and **D17** (100 μM in both bacteria).

The seven derivatives in this cluster are unified by a phenyl residue with an amino or (substituted) hydroxy group in the meta or para position (Table 1). **D11** and **D17** differ from the other members of the cluster by the etherification of their hydroxy groups. This suggests that the presence of a free amine (**D7**, **D8**) or alcohol (**D13**, **D20**, **D21**) in meta or para position is beneficial for the activity. This is in line with the eNTRy-rules for uptake in Gram-negative bacteria proposed by Richter et al., claiming the need for an ionizable nitrogen, among other criteria.^24^ A similar effect can be observed for tetramethylguanidinylation, as compound **D7-TMG** is comparably active in *S. aureus* and *E. coli* (25.0 μM and 50.0 μM, respectively). This further supports that the eNTRy-rules can be applied to our compounds.

All other derivatives are significantly less active in *E. coli*, with sulfolane **D12** being a rare exception (6.25 μM in *S. aureus*, 25.0 μM in *E. coli*). For the bis-acylated derivatives **D2** (12.5 μM and 50.0 μM), **D5** (12.5 μM and 50.0 μM), **D14** (3.13 μM and 25.0 μM) and **D6** (3.13 μM and >200 μM), there seems to be a trend of increasing activity with decreasing length of the alkyl linker.

For alkyl residues, there seems to be no uniform relation between activity and chain length. **D9** with an *n*-octyl-chain is surprisingly active in *S. aureus* (3.13 μM), while for *E. coli* shorter chains seem slightly favorable (**D4** (200 μM) and **D19** (100 μM)). With alkyne residues, a similar trend is observed: the short-chained **D1** is five times more active in *E. coli* than the long-chained variant **D15** (40.0 μM vs 200 μM), but they are equally active in *S. aureus* (100 μM).

#### ESKAPEE Panel, Resistance Generation and Toxicity Study

In conclusion, derivative **D8** with a *p*-aminophenyl residue was found to be the most potent hit in both *S. aureus* USA300 (a methicillin-resistant clinical isolate) and type strain *E. coli* K12 with MICs of 0.78 and 1.56 μM, respectively. Importantly, **D8** is much more active compared to the nitrofuran-containing, marketed antibiotics nitrofurantoin (100 μM in *S. aureus*, 50.0 μM in *E. coli*) and nitrofurazone (25.0 and 12.5 μM) shown in Figure 4b, highlighting the power of the DL-designed and -curated scaffolds.

**Figure 4:**
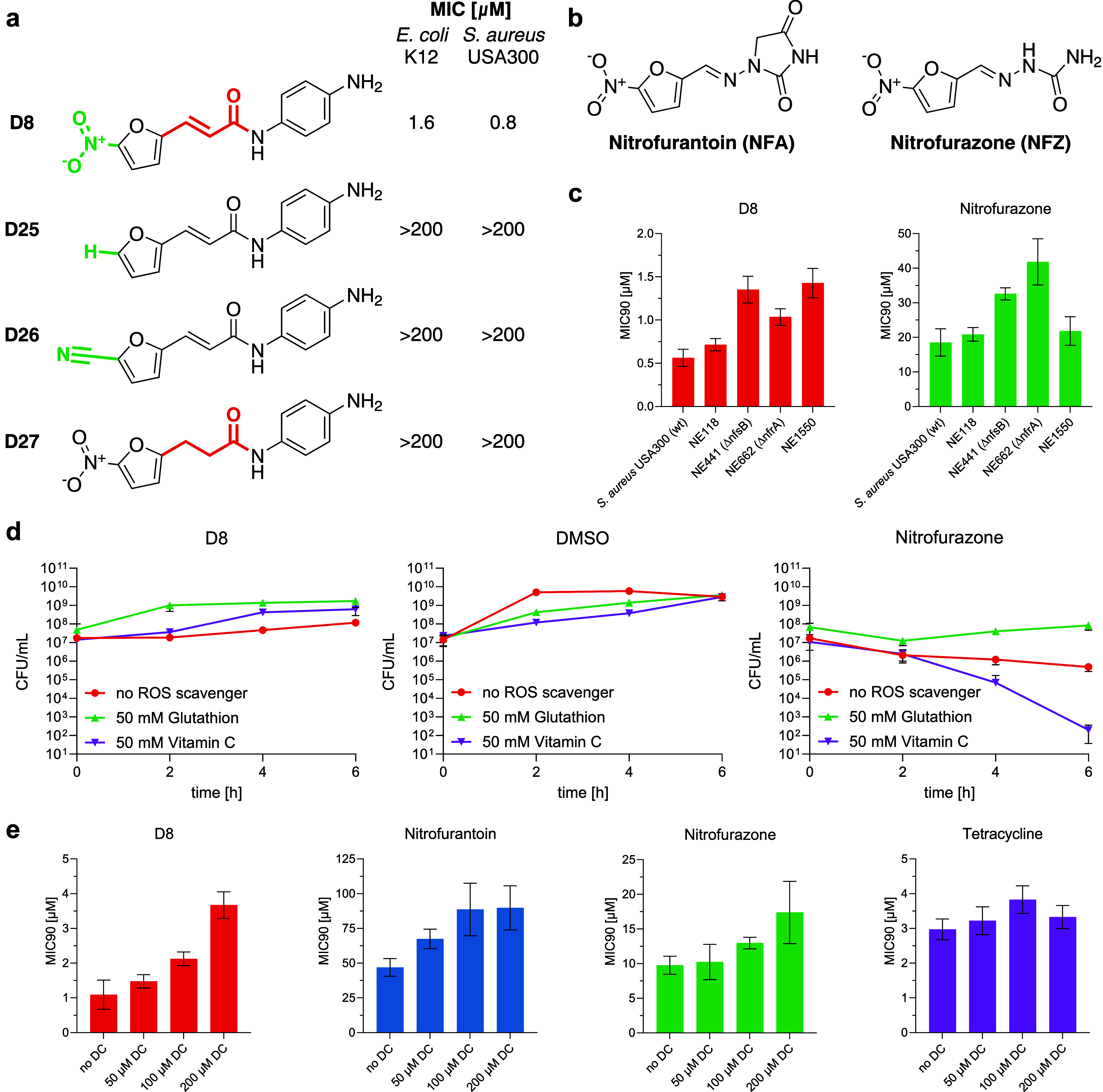
Assay-based mode of action studies. (a) Structures of **D8**-derivatives lacking essential functional groups along with their MICs in *E. coli* and *S. aureus*. (b) Structures of commercial nitrofuran antibiotics nitrofurantoin and nitrofurazone. (c) MIC_90_-values of antibiotics **D8** (red bars) and nitrofurazone (green bars) in wild-type *S. aureus* USA300 and four transposon mutants of different nitroreductases. Data represent mean ± std. of the EC_10_ determined from a nonlinear regression fitted to OD_600_ measurements of cultures incubated with different concentrations of the antibiotic for 16 h, determined in triplicates in *n* = 2 independent experiments. (d) Effect of ROS-scavengers l-glutathione (50 mM, green) and vitamin C (50 mM, purple) on the growth of *E. coli* K12 in the presence of antibiotics **D8** (16 μM), nitrofurazone (125 μM) or DMSO (1% (v/v)). After 0, 2, 4 and 6 h of incubation, viable cells (CFU/mL) were determined in triplicate at up to three different dilutions each. Data represent mean ± std. of *n* = 2 independent experiments. (e) MIC_90_-values of antibiotics **D8** (red bars), nitrofurantoin (blue bars), nitrofurazone (green bars) and tetracycline (purple bars) in *E. coli* K12 in the presence of increasing concentrations of nitroreductase-inhibitor dicoumarol (DC) (0, 50, 100 and 200 μM). Data represent mean ± std. of the EC_10_ determined from a nonlinear regression fitted to OD_600_ measurements of cultures incubated with different concentrations of the antibiotic for 16 h, determined in triplicates in *n* = 2 independent experiments.

Given its superior activity, **D8** was additionally tested for activity against all other ESKAPEE pathogens (Tab. S3). Type strain *Klebsiella pneumoniae* DSM 30104 and clinical isolate *E. coli* CFT073 showed low MICs of 3.13 μM and 6.25 μM, respectively. For *Enterococcus faecium* DSM 20477 (multiresistant type strain), clumpy growth prevented a reliable readout by optical density (OD_600_) measurement, but readout by eye showed an MIC of ∼50 μM. The multiresistant type strain *Enterobacter cloacae* DSM 30,054 showed a high MIC of 100 μM. *Acinetobacter baumannii* and *P. aeruginosa* are known to be intrinsically insensitive to nitrofuran antibiotics.^35,36^ This holds true for our compound, as multiresistant type strain *A. baumannii* DSM 30,007 and clinical isolate *P. aeruginosa* PAO1 did not show susceptibility to **D8** up to 200 μM.

A resistance generation assay by serial passage in *E. coli* K12 with **D8** and nitrofurantoin for 7 days showed 4- to 8-fold increased resistance development for **D8** compared to nitrofurantoin (see Table S4). However, the solubility limit of nitrofurantoin was reached at only 16-fold MIC (800 μM), limiting the generation of higher resistances. Resistant mutants of **D8** showed strong cross-resistance to nitrofurantoin and vice versa, suggesting a shared resistance mechanism (see Table S5).

**D8** and nitrofurantoin were tested for their toxicity on human cells in an MTT assay in human embryonal kidney cells (HEK293). With an IC_50_ of 10 μM, **D8** exhibited moderate toxicity, leaving a potential therapeutic window of 13-fold compared to its MIC in *S. aureus* and 6-fold compared to the MIC in *E. coli* (Figure S6). While the tested concentration range of marketed nitrofurantoin was not sufficient to reliably fit a nonlinear regression for IC_50_ determination, a 40% reduction in metabolic activity was observed at the highest concentration tested (200 μM). This suggests an IC_50_ of ∼250 μM, which is five times the MIC in *E. coli* and thus on par with the window observed for **D8**. Notably, albeit this narrow therapeutic window, nitrofurantoin is a first-line treatment for uncomplicated urinary tract infections (UTIs),^36^ for which *E. coli* is one of the most relevant pathogens.

### Mode of Action Determination

Remarkably, despite the market entry of nitrofuran antibiotics in the 1940s,^37^ very little is known about their MoA. They are prodrugs and require reductive activation of the nitro group in bacteria by NAD­(P)­H-dependent nitroreductases (NTRs). Oxygen-insensitive Type I NTRs perform consecutive two-electron reductions of the nitro group to form nitroso and hydroxylamino derivatives, which can react with cellular biomolecules (e.g., proteins and nucleic acids). On the other hand, nitrofurans are reduced to a radical anion by oxygen-sensitive Type II NTRs in a one-electron reduction, which is reoxidized in the presence of oxygen to form a superoxide radical (O_2_^•–^) and thus reactive oxygen species (ROS).^38–41^ *P. aeruginosa* is known to have a specific resistance against nitrofuran antibiotics, which we also observed for all our derivatives. This suggests a similar MoA for our derivatives.^35,36^ The main difference between known nitrofuran antibiotics and the molecules discovered here is the central bridge of the molecule: a hydrazone in marketed nitrofurans versus an acrylamide in our compounds. This acrylamide, an electrophilic Michael acceptor moiety, could either change the redox potential or enable a different MoA by covalently modifying nucleophilic side chains of biomolecules, such as proteins.

In order to elucidate the MoA, we conducted a series of complementary experiments to investigate whether our compounds share a common MoA with classical nitrofurans or act through a different mechanism. These included a “scaffold pruning” study to assess the contribution of key functional groups, time-kill assays with ROS scavengers to evaluate the role of oxidative stress, as well as inhibition and transposon mutagenesis of bacterial NTRs to probe bioactivation pathways. Finally, our MoA experiments culminate in mass spectrometry full proteome profiling, enabling quantitative assessment of global protein expression changes in response to antibacterial treatment.

#### Scaffold Pruning Study

We synthesized derivatives **D25–27** of our most potent hit **D8** and investigated the role of characteristic functional groups (Figure 4a, for synthetic route see Figures S20–S23). Replacing the nitro group of **D8** by a hydrogen atom (**D25**) or a similarly electron-withdrawing nitrile group (**D26**) resulted in complete loss of activity in both *S. aureus* and *E. coli*. In addition, a control compound lacking the Michael acceptor (**D27**) showed no activity. Interestingly, cyclic voltammetry measurements revealed a correlation between the redox potential and the antibiotic activity of nitrofurans (see Figure 4a, as well as Figures S7 and S8 and section “Cyclic Voltammetry Measurements” of the Supporting Information). The first reductive event for **D8** occurs at a cathodic peak potential (*E*_p,c_) of −420 mV against Ag/AgCl, which is significantly less negative than for the analogous reduction of nitrofurantoin (*E*_p,c_ = −580 mV) and **D27** (*E*_p,c_ = −680 mV). The more negative redox potential of **D27** shows that the redox potential depends on the overall size of the delocalized π-electron system and conjugation effects that connect the redox reactive site to electronic structure modulating substituents at the other end of the molecule. Changes in redox potential in this order of magnitude are known to be decisive for the rate of the reductive activation and generation of ROS under aerobic conditions, which is in line with the loss in activity.^42–45^ Taken together, these results suggest that on the one hand, the nitro group is essential for the activity, supporting an NTR-based mechanism, and on the other hand the reduction of the C=C double bond significantly alters the electrochemical potential required for enzymatic activation.

#### Assay-Based Mode of Action Elucidation

To further validate the hypothesis of a ROS-mediated MoA, we performed a time-kill-assay with **D8** in the presence of the ROS-scavengers glutathione (GSH) and vitamin C (50 mM) in *E. coli* to determine whether the presence of the scavengers restores bacterial growth.^46–48^ Nitrofurazone was included as a positive control (Figure 4d). In the vehicle control, both vitamin C and GSH initially slowed growth compared to dimethyl sulfoxide (DMSO), but all cultures reached similar bacterial densities after 6 h. At 10× MIC (16 μM), **D8** showed a bacteriostatic effect in the absence of scavengers. GSH provided immediate protection, restoring growth to the level of DMSO without scavengers. Vitamin C showed a delayed but significant protective effect. For nitrofurazone, GSH enhanced survival, while vitamin C led to a bactericidal effect after 6 h—even though pure nitrofurazone was only bacteriostatic at 10× MIC (125 μM).

Bacteria have several NTRs, though not all are well studied, and nitrofurans can likely be activated by multiple enzymes. We therefore used dicoumarol (DC), a known pan-NTR inhibitor,^49–51^ to determine the MICs of **D8**, positive controls nitrofurantoin and nitrofurazone, and the negative control tetracycline in *S. aureus* and *E. coli*, in the presence of increasing DC concentrations (0, 50, 100 and 200 μM). In *E. coli*, a concentration-dependent increase in MIC was observed for **D8** and the positive controls upon treatment with increasing concentrations of DC, whereas tetracycline’s MIC remained unaffected (Figure 4e). No effect was observed in *S. aureus*.

To further identify specific NTRs involved in activation, we repeated the experiment using various NTR transposon mutants from the Nebraska Transposon Mutant Library (NTML) for all four *S. aureus* USA300 proteins annotated as NTRs in UniProt.^52^ These include transposons of NtrA, previously shown to activate nitrofurans (NE1550),^53–55^ as well as homologues of well-known NfsA (NE662) and NfsB (NE441) from *E. coli*.^38,55–57^ Mutants NE441, NE1550, and NE662 showed clear MIC-shifts to higher concentrations for **D8**, which is in line with reduced susceptibility to nitrofurazone by NTR mutants NE441 and NE662 (Figure 4e). These results demonstrate the role of various NTRs in compound activation and induction of ROS.

#### Mass Spectrometry-Based Mode of Action Profiling

To additionally investigate the mechanistic similarity of our lead compound **D8** to known antibiotic classes, we performed a correlation analysis based on phenotypic changes in the *E. coli* proteome upon treatment with different antibiotics. The changes were determined by comparing the results of MS-based label-free full proteome analyses (Figure 5). Our results demonstrate a high Pearson correlation coefficient (*r* = 0.74–0.82) between compound **D8** and known nitrofuran antibiotics nitrofurantoin and nitrofurazone, strongly indicating a shared MoA.

**Figure 5:**
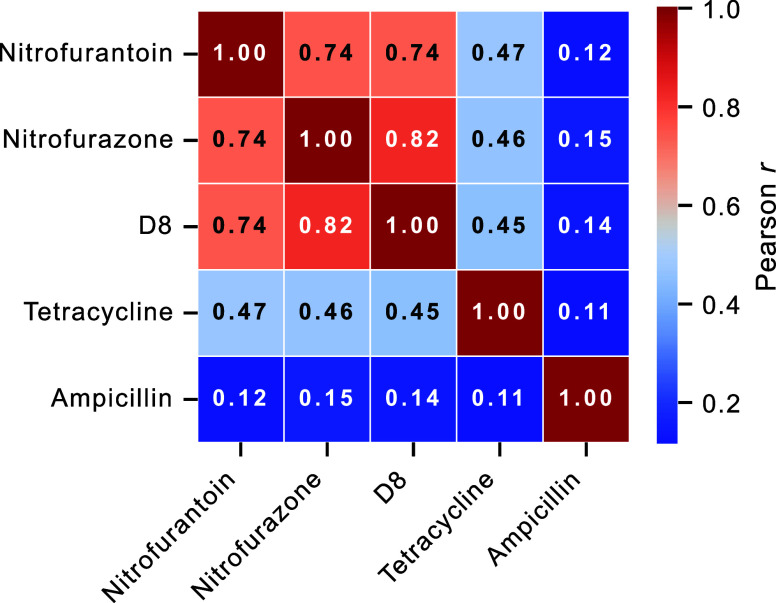
Mass spectrometry-based mode of action profiling. Label-free full proteome LC–MS/MS was performed on antibiotics-treated *E. coli* K12 cells. Differential protein abundances were quantified as log_2_ fold-changes relative to the DMSO control and subjected to significance filtering (adjusted *p* < 0.05). Shown is a heat map of Pearson correlation coefficients between the profiles of significantly altered proteins across *n* = 4 biological replicates per treatment. High Pearson *r* values across the antibiotics indicate shared MoA signatures, whereas lower *r* values between different antibiotics reflect distinct mechanisms.

In contrast, the correlation between **D8** and tetracycline, an antibiotic that belongs to a distinctly different class and inhibits bacterial protein synthesis,^58^ was considerably lower (*r* = 0.45). Importantly, we observed negligible correlation between **D8** and ampicillin, again underscoring significant mechanistic divergence from β-lactam antibiotics, which cause interference with the bacterial cell wall synthesis.^59,60^

Collectively, this data provides robust evidence for our hypothesis that **D8** shares its MoA with the nitrofuran antibiotic class, distinctively differing in antibacterial profile from antibiotics of other structural classes. Thus, our DL pipeline has successfully produced a novel structural variant that retains the validated and FDA-approved MoA of nitrofurans while showing significantly improved antibacterial activity.

## Conclusions

By applying transfer learning from structurally diverse known antibiotics to a CLM, and curating the generated molecules using predictive DL models and a chemist-in-the-loop approach, we created a library of synthetically accessible novel antibiotic candidates. Among the 11 molecules synthesized, nitrofuran **8** exhibited single-digit micromolar activity against a clinical methicillin-resistant *S. aureus* strain.

Based on this promising discovery, we trained an AutoML model on a Gram-negative accumulation data set. We subsequently selected 48 derivatives for a robot-assisted synthesis panel, resulting in 17 molecules with activity against *E. coli*. Our most potent derivative **D8** showed an MIC of 0.78 μM in *S. aureus* USA300 and 1.6 μM in *E. coli* K12, which represents a significant improvement over marketed nitrofurans. Importantly, **D8** showed only moderate toxicity toward human cells (IC_50_ 10 μM in HEK 293 cells) and a wider therapeutic window than the FDA-approved drug nitrofurantoin.

Mechanistic studies—including scaffold pruning, time-kill assays with ROS scavengers, and MIC profiling in bacteria with inhibited or knocked-out NTRs—support a mode of action similar to nitrofuran antibiotics. This is additionally backed by a correlation analysis based on phenotypic changes in the *E. coli* proteome upon treatment with different antibiotics, where **D8** showed a strong correlation with nitrofurantoin and nitrofurazone, and no correlation with the other classes tested.

Overall, our study demonstrates a powerful, validated end-to-end pipeline integrating state-of-the-art DL techniques, automated synthesis, and experimental validation. This approach accelerates novel antibiotic discovery, leading to the identification of a privileged scaffold with potential for structural diversification and compounds—such as **D8**—exhibiting significantly improved potency compared to classical nitrofurans. This success underscores the model’s ability to recognize key pharmacophores and tailor structures to meet specific design constraints, such as optimizing Gram-negative cellular uptake, as represented by the substantial gain in Gram-negative potency after AutoML-guided derivatization. Our results support the practical application of these integrated techniques, establishing a benchmark for their application and highlighting their potential in addressing the urgent need for new antibiotics. Furthermore, this work provides a clear framework for future collaborative drug discovery, demonstrating the synergy achieved when medicinal chemistry, synthetic chemistry, and computer science interact effectively.

## Supplementary Material



## Data Availability

Our code and data is deposited on GitHub https://github.com/sieber-lab/AIbiotics. Further, our mass spectrometry data can be accessed on PRIDE with the data set identifier PXD066005. The system used for computational work is equipped with an AMD Ryzen Threadripper PRO 5995WX CPU with 64/128 cores/threads and 1024 GB RAM. The server is also powered by an NVIDIA RTX 4090 GPU with 24 GB VRAM. The Gram-negative accumulation data set can be found at https://github.com/HergenrotherLab/GramNegAccum.

## Notes

The Supporting Information contains additional results and discussion as well as the methods section (PDF)
