# Supplementary material for: Generative Deep Learning Pipeline Yields Potent Gram-Negative Antibiotics
Source: JACS Au. 2025 Sep 9;5(9):4249–59. doi: 10.1021/jacsau.5c00602 (PMC12458054; doi:10.1021/jacsau.5c00602)
Supplement: Supplementary file 1 [file au5c00602_si_001.pdf]

Supporting Information: Generative Deep Learning Pipeline Yields Potent Gram-negative Antibiotics

Martin F. Köllen,[†,‖] Maximilian G. Schuh,[†,‖] Robin Kretschmer,[†] Joshua Hesse,[†] Dominik Schum,[†] Junhong Chen,[‡] Annkathrin I. Bohne,[¶] Dominik P. Halter,[‡,§] and Stephan A. Sieber[*,†]

†*Technical University of Munich, TUM School of Natural Sciences, Department of Bioscience, Center for Functional Protein Assemblies (CPA), Chair of Organic Chemistry II, 85748 Garching bei München, Germany*

‡*Technical University of Munich, TUM School of Natural Sciences, Department of Chemistry, Catalysis Research Center (CRC), Chair of Inorganic and Metal-Organic Chemistry, 85748 Garching bei München, Germany*

¶*Technical University of Munich, TUM School of Natural Sciences, Department of Bioscience, Center for Functional Protein Assemblies (CPA), Chair of Biochemistry, 85748 Garching bei München, Germany*

§*University of Antwerp, Faculty of Applied Engineering, Department of Biochemical and Chemical Engineering, Research Group Applied Electrochemistry & Catalysis (ELCAT), 2610 Antwerp, Belgium.*

‖*Contributed equally to this work*

E-mail: stephan.sieber@tum.de

Additional Results and Discussion

TwinBooster Accumulation Prediction

This represents the original and unchanged text prompt, taken from the Richter et al. publication, used as TwinBooster prediction input (original text quoted as received):[S1]

> Accumulation of drugs in Gram-negative bacteria. The accumulation method was adapted from known protocols13,14,18 and liquid chromatography with tandem mass spectroscopy (LC–MS/MS) was used to quantify accumulation of each compound. The assay method was evaluated with antibiotics that have known high (tetracycline, ciprofloxacin and chloramphenicol) and low (novobiocin, erythromycin, rifampicin, vancomycin, daptomycin, clindamycin, mupirocin and fusidic acid) levels of accumulation. Ampicillin was also used as a 'low-accumulation' control as it is rapidly covalently appended to penicillin-binding proteins, preventing measurement by LC–MS/MS. To account for the possibility of non-specific binding to the outer membrane, Gram-negative-active antibiotics with various charged states at physiological pH were chosen: tetracycline (positively charged), ciprofloxacin (zwitterionic) and chloramphenicol (neutral) (structures shown in Supplementary Table 1). The results show a significantly higher level of accumulation in E. coli for the Gram negative-active compounds as compared to compounds with low Gram-negative antibacterial activity and ampicillin, consistent with measurement of accumulation as opposed to non-specific binding (Fig. 1a). To further ensure that variations in observed accumulation levels were not due to differences in non-specific affinity for the membrane, penetrance was perturbed by co-treating E. coli with the membrane-permeabilizing agent colistin19; an increase in accumulation for low-accumulating antibiotics (novobiocin, erythromycin, rifampicin and fusidic acid) was observed in this experiment (Fig. 1b). The accumulation assay was performed in triplicate in batches of

ten samples, with each batch containing either tetracycline or ciprofloxacin as a positive control. E. coli MG1655 was used for these experiments as this strain has been only minimally altered from its K-12 progenitor33. For each replicate, 2.5 ml of an overnight culture of E. coli was diluted into 250 ml of fresh Luria Bertani (LB) broth (Lennox) and grown at 37 °C with shaking to an optical density (OD600) of 0.55. The bacteria were pelleted at 3,220 r.c.f. for 10 min at 4 °C and the supernatant was discarded. The pellets were re-suspended in 40 ml of phosphate buffered saline (PBS) and pelleted as before, and the supernatant was discarded. The pellets were re-suspended in 8.8mL of fresh PBS and aliquoted into ten 1.5ml eppendorf tubes (875µl each). The number of colony-forming units (CFUs) was determined by a calibration curve. The samples were equilibrated at 37 °C with shaking for 5 min, compound was added (final concentration =50 µM), and then samples were incubated at 37 °C with shaking for 10 min. A 10 min time point was chosen because it is longer than the predicted amount of time required to reach a steady-state concentration34, but short enough to minimize metabolic and growth changes (no changes in OD600 observed; CFUs were reduced by a factor of five after ciprofloxacin treatment for 10 min, but no other antibiotics had an effect). After incubation, 800 µl of the cultures were carefully layered on 700 µl of silicone oil (9:1 AR20/Sigma High Temperature, cooled to −78 °C). Bacteria were pelleted through the oil by centrifuging at 13,000 r.c.f. for 2 min at room temperature (supernatant remains above the oil); the supernatant and oil were then removed by pipetting. To lyse the samples, each pellet was dissolved in 200 µl of water, and then they were subjected to three freeze-thaw cycle of three minutes in liquid nitrogen followed by three minutes in a water bath at 65 °C. The lysate was pelleted at 13,000 r.c.f. for 2 min at room temperature and the supernatant was collected (180 µl). The debris was re-suspended in 100µl of methanol and pelleted as before. The supernatants

were removed and combined with the previous supernatants collected. Finally, remaining debris was removed by centrifuging at 20,000 r.c.f. for 10 min at room temperature. Supernatants were analysed by LC–MS/MS. Samples were analysed with the 5500 QTRAP LC/MS/MS system (AB Sciex) with a 1200 series HPLC system (Agilent Technologies) including a degasser, an autosampler, and a binary pump. The liquid chromatography separation was performed on an Agilent SB-Aq column (4.6 × 50 mm, 5 µm) (Agilent Technologies) with mobile phase A (0.1 % formic acid in water) and mobile phase B (0.1 % formic acid in acetonitrile). The flow rate was 0.3 ml min−1. The linear gradient was as follows: 0–3 min, 100 % mobile phase A; 10–15 min, 2 % mobile phase A; 15.521 min, 100 % mobile phase A. The autosampler was set at 5 °C. The injection volume was 15 µl. Mass spectra were acquired with both positive electrospray ionization at the ion spray voltage of 5,500 V and negative electrospray ionization at the ion spray voltage of −4,500 V. The source temperature was 450 °C. The curtain gas, ion source gas 1, and ion source gas 2 were 33, 50 and 65, respectively. Multiple reaction monitoring was used to quantify metabolites. Power analysis was not used to determine the number of replicates. Error bars represent the standard error of the mean of three biological replicates. All compounds evaluated in biological assays were ≥95 % pure. (Richter et al.[S2])

Accumulation Validation We validated our accumulation prediction using a weighted prediction approach and compared it with zero-shot and automated machine learning (AutoML) models. To assess predictive performance, we conducted a stratified 10-fold cross-validation on the training dataset from Richter et al.[S2] Fig. S1 summarizes model performance in terms of precision recall area under curve (PR AUC) and receiver operating characteristic area under curve (ROC AUC). While TwinBooster, a zero-shot model not specifically fine-tuned for chemical data or accumulation prediction, demonstrated solid performance

(ROC AUC = 0.655 ± 0.193), AutoML provided notable improvements over the zero-shot baseline (ROC AUC = 0.933 ± 0.112). However, our weighted prediction method, which incorporates training set similarity, achieved the highest performance and lowest standard deviation (ROC AUC = 0.955 ± 0.103). Thus, this robust and accurate weighted model was selected for downstream predictions.

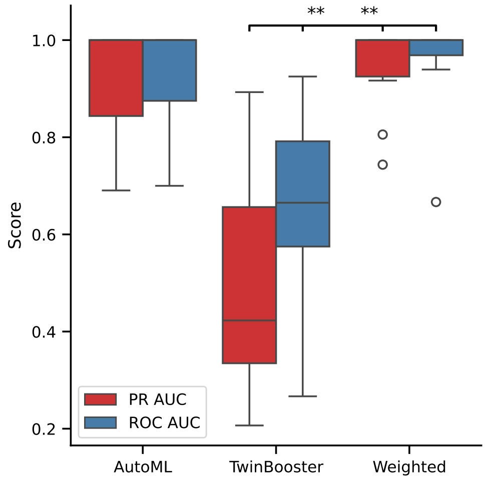

Figure S1: Cross-validation performance of accumulation prediction models on the Richter et al. dataset. Predictive performance was evaluated using stratified 10-fold cross-validation and is reported in terms of PR AUC and ROC AUC. The weighted prediction model, which accounts for training set similarity, outperformed both the zero-shot TwinBooster model and AutoML, achieving the highest scores with the lowest variability (**$p < 0.01$, Wilcoxon test).

Scaffold Diversity Enhances Generative Model Output

To assess the impact of scaffold diversity on model output, we compared molecules generated by the same chemical language model (CLM) procedure fine-tuned on two datasets: the 2236 full antibiotic structure set and a curated subset of 131 structurally diverse scaffolds. The curated set was constructed by removing close analogues to avoid overrepresentation of any single scaffold class. Pairwise Tanimoto similarity analysis revealed that molecules generated from the diverse scaffold set exhibited significantly lower structural similarity (Fig. S2), indicating greater chemical diversity and reduced risk of mode collapse or memorization. This confirms that scaffold selection plays a critical role in shaping the diversity of generated

compounds.

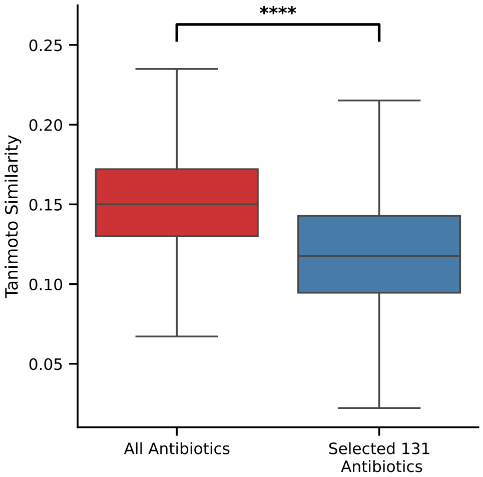

Figure S2: Pairwise Tanimoto similarity of generated molecules fine-tuned on either the full antibiotic set or the curated subset of 131 diverse scaffolds. Molecules generated using the 131 scaffold set exhibit significantly lower structural similarity (****$p < 0.0001$, Mann–Whitney U test), indicating enhanced chemical diversity and reduced redundancy. Outliers are not shown.

De Novo Similarity Analysis

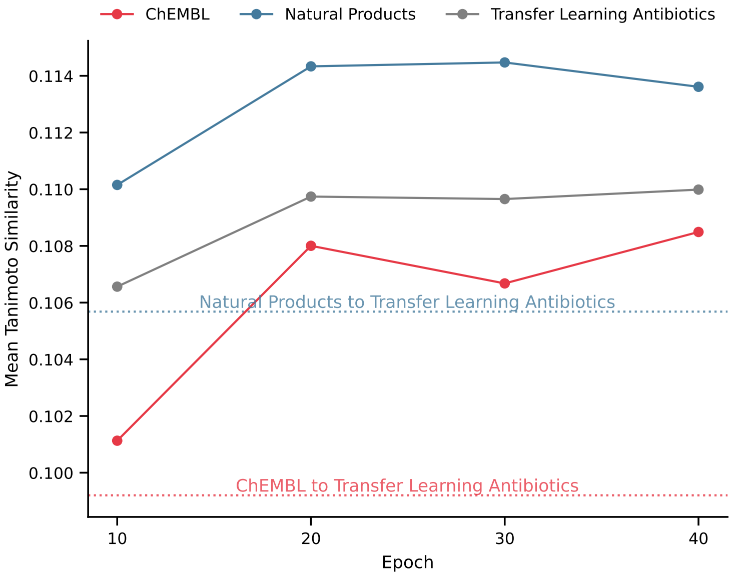

Figure S3: Mean Tanimoto similarity.

To assess the similarity between the generated molecules and the training data, we analyzed both Tanimoto similarity and Fréchet ChemNet distance (FCD). We compared all

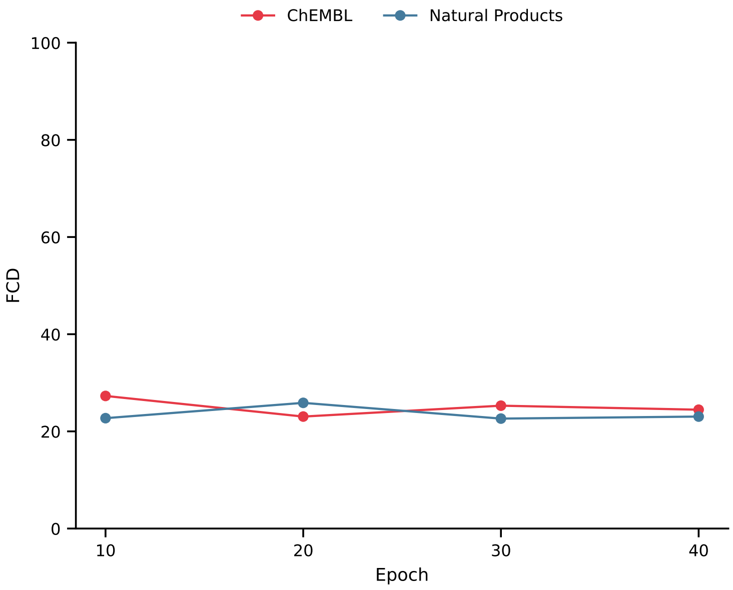

Figure S4: Frechet ChemNet distance.

2239 *de novo* designs to their respective training sets using Tanimoto similarity (Fig. S3). Overall, we observed a low initial similarity, which gradually increased during training. This aligns with our expectations, as the transfer learning sets are intended to shape the structural properties of the generated molecules. Additionally, FCD analysis indicates that while the generated molecules deviate from existing "real" compounds, they remain within a viable chemical space suitable for real-world applications (Fig. S4).[S3]

D8 Structural Comparison To further assess the novelty of **D8**, we compared its structure to the 131 scaffolds used for model fine-tuning using extended-connectivity fingerprint (ECFP)-based Tanimoto similarity (Fig. S5). The similarity values are consistently low ($s < 0.4$), indicating limited structural overlap with the training set. This analysis highlights that **D8** is not only structurally distinct from the fine-tuning scaffolds but also diverges from traditional nitrofuran-containing antibiotics, underscoring its chemical novelty.

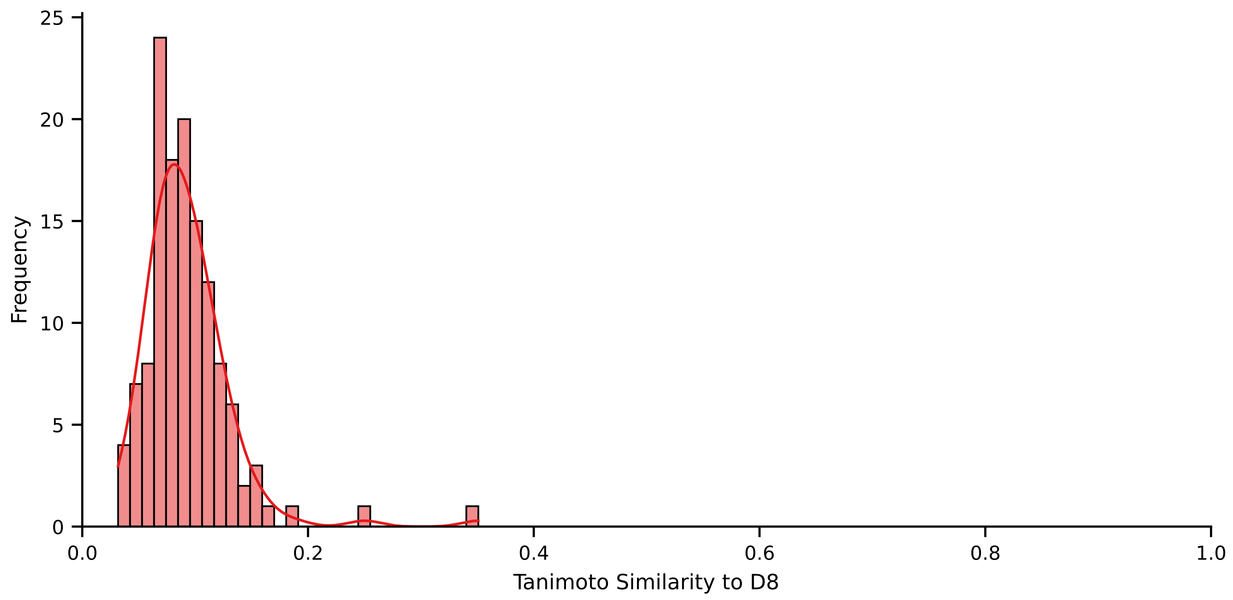

Figure S5: Tanimoto similarity distribution of **D8** compared to the 131 fine-tuning antibiotic scaffolds, computed using ECFP. The fingerprints show consistently low similarity values, indicating that **D8** occupies a distinct region of chemical space relative to the training set.

SAR Study

The 48 derivatives with the highest accumulation score were synthesized on a 0.1 mmol-scale in a robot-assisted parallelized synthesis panel, employing a *O*-(7-azabenzotriazol-1-yl)-1,1,3,3-tetramethyluronium hexafluorophosphate (HATU)-mediated amide coupling protocol in a deep well plate, using a mixture of acetonitrile (ACN) and dimethyl sulfoxide (DMSO) as the solvent and *N N*-diisopropylethylamine (DIPEA) as the base. After completion of the reaction, the reaction mixtures were diluted in water to precipitate the product. The precipitates were collected by centrifugation, washed with water and dried. The crude products obtained this way were tested by high-performance liquid chromatography–mass spectrometry (HPLC–MS) for the presence of the desired product and all successfully synthesized derivatives were tested for their activity in *S. aureus* USA300, *E. coli* K12 and *P. aeruginosa* PAO1 at fixed concentrations of 50, 100 and 200 µM (Tab. S1).

For 24 of the 45 crude products obtained after precipitation, the HPLC–MS analysis showed pure amide coupling products. 16 crude products contained impurities, and 5

contained no species identifiable by HPLC–MS. For 13 of the 16 impure products, the amine precursors had multiple unprotected reactive sites (e.g., diamines, hydrazines, amino alcohols), leading to bis-acylation or tetramethylguanidinylation. The remaining three had unidentifiable impurities.

Table S1: *(Table starts on the next page.)* Molecular structures, HPLC–MS results and antibiotic activity of the 45 derivatives of compound **8** isolated by precipitation from the reaction mixtures of the automated synthesis panel. For the different bacteria and concentrations, the bacterial growth (OD_{600} after 16 h) from three biological replicates is given in the table. To simplify interpretation, growth is also represented by circles in three colors: white for no growth ($OD_{600} < 0.1$), gray for reduced growth ($0.1 \leq OD_{600} \leq 0.3$), and black for full growth ($OD_{600} > 0.3$).

Regioisomer or other sideproduct possible during HATU coupling

TMG = tetramethylguanidinium

Compound	structure of main product	Exact Mass (calculated as [M+H]+)	measured HRMS	accuracy [ppm]	purity by HPLC-MS after precipitation	*Staphylococcus aureus* USA300									*Escherichia coli* K12									*Pseudomonas aeruginosa* PAO1								
						50 µM			100 µM			200 µM			50 µM			100 µM			200 µM			50 µM			100 µM			200 µM		
						1	2	3	1	2	3	1	2	3	1	2	3	1	2	3	1	2	3	1	2	3	1	2	3	1	2	3
Compound 8 (MAK-1-334)	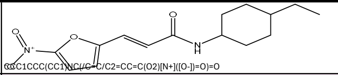 CCC1CCC(CC1)NC(/C=C/C2=CC=C(O2)[N+]([O-])=O)=O	293.14958	293.14937	0.73	pure (mixture of stereoisomers)	0.07	0.05	0.05	0.06	0.05	0.05	0.06	0.05	0.06	0.55	0.65	0.56	0.65	0.58	0.51	0.52	0.54	0.51	0.64	0.70	0.67	0.67	0.73	0.84	0.73	0.75	0.84
MAK-1-359	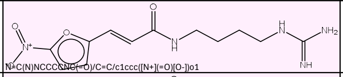 N=C(N)NCCCCNC(=O)/C=C/c1ccc([N+](=O)[O-])o1	296.13533	296.13509	0.81	pure	0.89	0.86	0.84	0.83	0.68	0.74	0.61	0.21	0.48	0.55	0.66	0.59	0.63	0.58	0.58	0.63	0.59	0.58	0.72	0.79	0.77	0.77	0.84	0.84	0.83	0.84	0.79
MAK-1-360	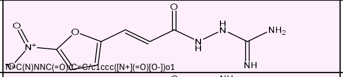 N=C(N)NNC(=O)/C=C/c1ccc([N+](=O)[O-])o1	240.07273	not found		no product found	0.86	0.86	0.82	0.81	0.68	0.76	0.62	0.55	0.57	0.56	0.64	0.54	0.62	0.59	0.54	0.59	0.56	0.54	0.72	0.78	0.75	0.77	0.82	0.71	0.77	0.79	0.76
MAK-1-361	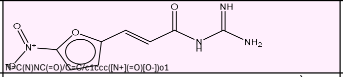 N=C(N)NC(=O)/C=C/c1ccc([N+](=O)[O-])o1	225.06183	225.06175	0.36	low	0.86	0.86	0.79	0.76	0.62	0.62	0.54	0.52	0.42	0.58	0.69	0.59	0.69	0.62	0.56	0.63	0.60	0.56	0.74	0.80	0.81	0.76	0.81	0.73	0.78	0.79	0.85
MAK-1-362	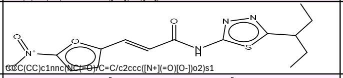 CCC(CC)c1nnc(NC(=O)/C=C/c2ccc([N+](=O)[O-])o2)s1	337.09650	337.09614	1.07	medium, main peak is product, one medium peak with 435.18033	0.79	0.84	0.86	0.69	0.70	0.86	0.74	0.67	0.76	0.62	0.69	0.58	0.70	0.61	0.55	0.62	0.57	0.58	0.75	0.79	0.78	0.80	0.82	0.87	0.81	0.78	0.94
MAK-1-363	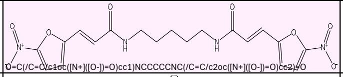 O=C(/C=C/c1oc([N+]([O-])=O)cc1)NCCCCCNC(/C=C/c2oc([N+]([O-])=O)cc2)=O	433.13539	433.13485	1.25	medium, two big & one small peak with product mass, one small peak with 366.21310 (TMG-sideproduct)	0.22	0.13	0.05	0.05	0.05	0.05	0.05	0.15	0.44	0.58	0.65	0.53	0.22	0.06	0.07	0.05	0.05	0.25	0.74	0.79	0.74	0.76	0.80	0.85	0.80	0.83	0.87
MAK-1-364	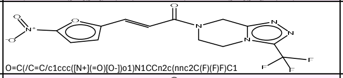 O=C(/C=C/c1ccc([N+](=O)[O-])o1)N1CCn2c(nnc2C(F)(F)F)C1	358.07576	358.07542	0.96	pure	0.25	0.29	0.22	0.05	0.05	0.05	0.05	0.05	0.05	0.60	0.68	0.58	0.57	0.42	0.38	0.10	0.07	0.11	0.72	0.77	0.73	0.75	0.79	0.83	0.76	0.78	0.91
MAK-1-365	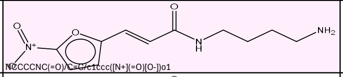 NCCCCNC(=O)/C=C/c1ccc([N+](=O)[O-])o1	254.11353	254.11339	0.56	very low, additional peaks with 352.19746 (TMG-sideproduct) and 419.11920 (bis-acylated product)	0.82	0.89	0.82	0.71	0.72	0.77	0.72	0.76	0.83	0.57	0.68	0.59	0.77	0.58	0.55	0.61	0.55	0.59	0.76	0.81	0.73	0.84	0.82	0.92	0.84	0.86	0.96
MAK-1-366	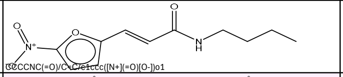 CCCCNC(=O)/C=C/c1ccc([N+](=O)[O-])o1	239.10263	239.10249	0.60	pure	0.47	0.55	0.57	0.18	0.18	0.16	0.05	0.05	0.05	0.56	0.67	0.56	0.43	0.24	0.26	0.05	0.05	0.05	0.71	0.77	0.69	0.75	0.77	0.83	0.76	0.77	0.89
MAK-1-367	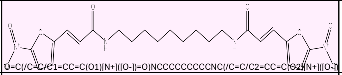 O=C(/C=C/C1=CC=C(O1)[N+]([O-])=O)NCCCCCCCCCNC(/C=C/C2=CC=C(O2)[N+]([O-]	489.19799	489.19721	1.59	high, big product peak, one big peak with 422.27557 (TMG-sideproduct)	0.76	0.86	0.83	0.76	0.71	0.79	0.81	0.76	0.69	0.53	0.59	0.52	0.59	0.53	0.49	0.56	0.52	0.52	0.71	0.74	0.73	0.73	0.76	0.89	0.76	0.76	0.89
MAK-1-368	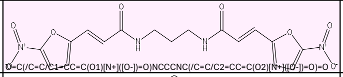 O=C(/C=C/C1=CC=C(O1)[N+]([O-])=O)NCCCNC(/C=C/C2=CC=C(O2)[N+]([O-])=O)=O	405.10409	405.10366	1.06	medium, one big and one medium peak with product mass, one small peak with 338.18187 (TMG-sideproduct)	0.22	0.06	0.05	0.19	0.05	0.05	0.05	0.05	0.05	0.55	0.62	0.46	0.26	0.13	0.09	0.05	0.05	0.05	0.69	0.72	0.71	0.70	0.75	0.91	0.71	0.74	0.81
MAK-1-358	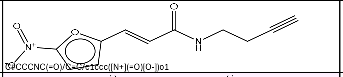 C#CCCNC(=O)/C=C/c1ccc([N+](=O)[O-])o1	235.07133	235.07117	0.69	pure	0.39	0.27	0.30	0.15	0.05	0.07	0.05	0.05	0.05	0.45	0.60	0.47	0.05	0.05	0.10	0.05	0.05	0.05	0.68	0.73	0.70	0.71	0.73	0.89	0.74	0.75	0.81
MAK-1-370	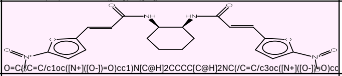 O=C(/C=C/c1oc([N+]([O-])=O)cc1)N[C@H]2CCCC[C@H]2NC(/C=C/c3oc([N+]([O-])=O)cc	445.13539	445.13467	1.62	very low, additional peaks with 280.12886 (mono-acylated product) and 378.21313 (TMG-sideproduct)	0.05	0.05	0.05	0.05	0.05	0.05	0.05	0.05	0.05	0.60	0.69	0.60	0.68	0.60	0.55	0.55	0.55	0.51	0.68	0.75	0.70	0.71	0.76	0.86	0.72	0.77	0.84
MAK-1-371	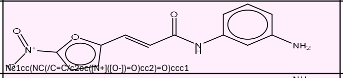 Nc1cc(NC(/C=C/c2oc([N+]([O-])=O)cc2)=O)ccc1	274.08223	274.08203	0.74	medium, small product peak, big peak with 372.16644 (TMG-sideproduct)	0.05	0.05	0.05	0.05	0.06	0.05	0.06	0.06	0.07	0.16	0.31	0.22	0.07	0.06	0.08	0.07	0.06	0.05	0.70	0.81	0.74	0.76	0.76	0.85	0.79	0.75	0.86
MAK-1-372	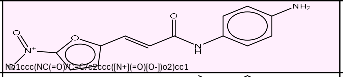 Nc1ccc(NC(=O)/C=C/c2ccc([N+](=O)[O-])o2)cc1	274.08223	274.08203	0.74	medium, small product peak, big peak with 372.16652(TMG-sideproduct)	0.06	0.06	0.06	0.10	0.11	0.08	0.11	0.09	0.09	0.07	0.28	0.21	0.09	0.08	0.08	0.11	0.09	0.10	0.71	0.78	0.71	0.75	0.74	0.87	0.71	0.74	0.85
MAK-1-373	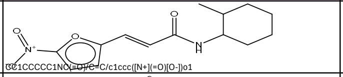 CC1CCCCC1NC(=O)/C=C/c1ccc([N+](=O)[O-])o1	279.13393	279.13379	0.51	pure, double peak (one big, one small) of two diastereomers	0.38	0.38	0.45	0.07	0.05	0.13	0.05	0.05	0.05	0.59	0.68	0.58	0.65	0.55	0.49	0.50	0.45	0.46	0.65	0.72	0.67	0.65	0.75	0.82	0.68	0.66	0.76
MAK-1-374	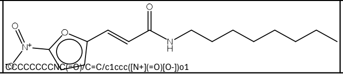 CCCCCCCCNC(=O)/C=C/c1ccc([N+](=O)[O-])o1	295.16523	295.16505	0.62	pure	0.05	0.06	0.07	0.05	0.06	0.05	0.06	0.06	0.06	0.46	0.58	0.50	0.52	0.55	0.42	0.22	0.34	0.33	0.71	0.73	0.69	0.69	0.76	0.83	0.78	0.76	0.88

MAK-1-376	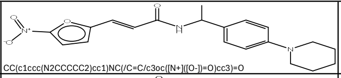 CC(c1ccc(N2CCCCC2)cc1)NC(/C=C/c3oc([N+]([O-])=O)cc3)=O	370.17613	370.17585	0.76	pure	0.19	0.15	0.14	0.05	0.05	0.06	0.06	0.06	0.06	0.57	0.66	0.56	0.66	0.60	0.53	0.57	0.53	0.52	0.66	0.71	0.67	0.66	0.70	0.84	0.70	0.72	0.85
MAK-1-377	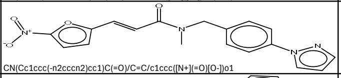 CN(Cc1ccc(-n2cccn2)cc1)C(=O)/C=C/c1ccc([N+](=O)[O-])o1	353.12443	353.12416	0.77	pure	0.22	0.18	0.18	0.05	0.05	0.05	0.06	0.05	0.05	0.59	0.66	0.56	0.58	0.52	0.45	0.36	0.41	0.36	0.63	0.72	0.66	0.69	0.68	0.78	0.67	0.65	0.82
MAK-1-379	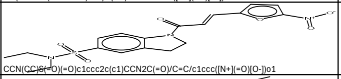 CCN(CC)S(=O)(=O)c1ccc2c(c1)CCN2C(=O)/C=C/c1ccc([N+](=O)[O-])o1	422.13803	not found		no product found	0.67	0.82	0.68	0.63	0.66	0.73	0.69	0.62	0.57	0.58	0.69	0.57	0.67	0.58	0.53	0.56	0.53	0.49	0.65	0.73	0.66	0.68	0.71	0.80	0.67	0.69	0.82
MAK-1-380	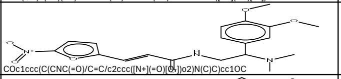 COc1ccc(C(CNC(=O)/C=C/c2ccc([N+](=O)[O-])o2)N(C)C)cc1OC	390.16596	390.16589	0.18	pure	0.05	0.19	0.22	0.05	0.05	0.05	0.05	0.05	0.05	0.59	0.68	0.57	0.66	0.59	0.51	0.50	0.52	0.51	0.64	0.71	0.66	0.69	0.70	0.79	0.67	0.62	0.81
MAK-1-381	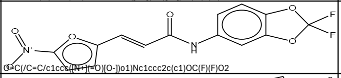 O=C(/C=C/c1ccc([N+](=O)[O-])o1)Nc1ccc2c(c1)OC(F)(F)O2	339.04232	339.04211	0.61	medium (probably), compound does not ionize well	0.05	0.05	0.05	0.06	0.06	0.08	0.10	0.10	0.10	0.53	0.63	0.54	0.63	0.56	0.48	0.49	0.47	0.46	0.67	0.71	0.68	0.69	0.75	0.84	0.73	0.70	0.85
MAK-1-383	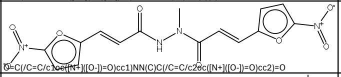 O=C(/C=C/c1oc([N+]([O-])=O)cc1)NN(C)C(/C=C/c2oc([N+]([O-])=O)cc2)=O	377.07279	377.07224	1.46	pure	0.18	0.06	0.19	0.87	0.05	0.05	0.05	0.05	0.05	0.60	0.68	0.56	0.65	0.50	0.52	0.20	0.23	0.17	0.68	0.70	0.69	0.66	0.66	0.87	0.72	0.71	0.84
MAK-1-384	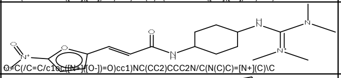 O=C(/C=C/c1oc([N+]([O-])=O)cc1)NC(CC2)CCC2N/C(N(C)C)=[N+](C)C	378.21358	378.21338	0.53	pure	0.75	0.82	0.83	0.35	0.67	0.79	0.63	0.58	0.70	0.57	0.66	0.54	0.68	0.55	0.55	0.57	0.55	0.54	0.71	0.70	0.70	0.70	0.73	0.85	0.72	0.74	0.87
MAK-1-385	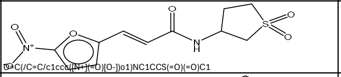 O=C(/C=C/c1ccc([N+](=O)[O-])o1)NC1CCS(=O)(=O)C1	301.04888	301.04852	1.21	pure	0.05	0.05	0.05	0.05	0.05	0.05	0.05	0.05	0.05	0.33	0.29	0.21	0.05	0.05	0.05	0.05	0.05	0.05	0.65	0.75	0.67	0.71	0.72	0.84	0.67	0.64	0.52
MAK-1-386	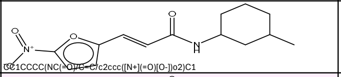 CC1CCCC(NC(=O)/C=C/c2ccc([N+](=O)[O-])o2)C1	279.13393	279.13377	0.58	pure	0.33	0.25	0.15	0.05	0.05	0.05	0.05	0.05	0.05	0.59	0.67	0.53	0.68	0.59	0.53	0.37	0.41	0.44	0.63	0.66	0.63	0.65	0.70	0.82	0.67	0.69	0.79
MAK-1-387	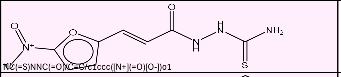 NC(=S)NNC(=O)/C=C/c1ccc([N+](=O)[O-])o1	257.03390	not found		no product found	0.16	0.06	0.07	0.07	0.07	0.06	0.09	0.08	0.08	0.43	0.56	0.41	0.06	0.06	0.07	0.07	0.07	0.07	0.65	0.71	0.67	0.70	0.76	0.86	0.72	0.71	0.78
MAK-1-388	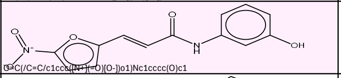 O=C(/C=C/c1ccc([N+](=O)[O-])o1)Nc1cccc(O)c1	275.06625	275.06601	0.86	high, medium product peak and big peak with 373.15047 (TMG-sideproduct)	0.05	0.05	0.05	0.05	0.06	0.05	0.10	0.11	0.09	0.05	0.05	0.05	0.05	0.05	0.05	0.09	0.09	0.07	0.62	0.73	0.65	0.66	0.72	0.81	0.67	0.65	0.70
MAK-1-389	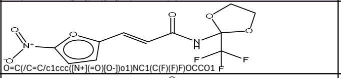 O=C(/C=C/c1ccc([N+](=O)[O-])o1)NC1(C(F)(F)F)OCCO1	324.05637	not found		no product found	0.70	0.77	0.66	0.44	0.45	0.53	0.17	0.19	0.17	0.55	0.63	0.49	0.50	0.44	0.36	0.09	0.05	0.21	0.67	0.71	0.66	0.67	0.67	0.84	0.68	0.74	0.86
MAK-1-390	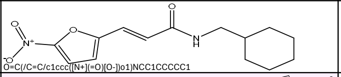 O=C(/C=C/c1ccc([N+](=O)[O-])o1)NCC1CCCCC1	279.13393	279.13379	0.51	pure	0.22	0.24	0.21	0.05	0.81	0.05	0.05	0.05	0.05	0.57	0.66	0.53	0.42	0.54	0.50	0.23	0.29	0.38	0.64	0.69	0.64	0.64	0.70	0.85	0.65	0.72	0.78
MAK-1-391	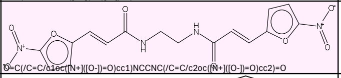 O=C(/C=C/c1oc([N+]([O-])=O)cc1)NCCNC(/C=C/c2oc([N+]([O-])=O)cc2)=O	391.08844	391.08794	1.28	high, big product peak and small peak with 324.16620 (TMG-sideproduct)	0.05	0.05	0.05	0.05	0.05	0.05	0.05	0.06	0.07	0.38	0.23	0.14	0.05	0.05	0.05	0.06	0.11	0.12	0.65	0.72	0.71	0.72	0.74	0.91	0.69	0.77	0.88
MAK-1-392	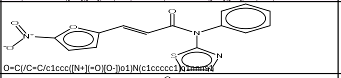 O=C(/C=C/c1ccc([N+](=O)[O-])o1)N(c1ccccc1)c1nnns1	345.05263	not found		no product found	0.44	0.57	0.45	0.45	0.36	0.37	0.30	0.17	0.23	0.55	0.65	0.54	0.66	0.55	0.54	0.49	0.54	0.56	0.65	0.71	0.70	0.70	0.75	0.85	0.70	0.73	0.89
MAK-1-393	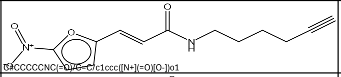 C#CCCCCNC(=O)/C=C/c1ccc([N+](=O)[O-])o1	263.10263	263.10248	0.58	pure	0.40	0.33	0.32	0.13	0.05	0.06	0.05	0.05	0.05	0.59	0.70	0.55	0.49	0.45	0.43	0.05	0.05	0.06	0.67	0.72	0.67	0.70	0.73	0.82	0.66	0.74	0.84
MAK-1-394	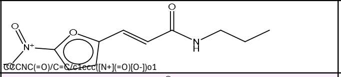 CCCNC(=O)/C=C/c1ccc([N+](=O)[O-])o1	225.08698	225.08681	0.77	pure	0.81	0.84	0.71	0.60	0.49	0.49	0.29	0.24	0.20	0.57	0.68	0.55	0.64	0.55	0.46	0.13	0.14	0.34	0.67	0.74	0.69	0.68	0.73	0.81	0.67	0.73	0.82
MAK-1-395	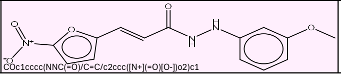 COc1cccc(NNC(=O)/C=C/c2ccc([N+](=O)[O-])o2)c1	304.09280	304.09251	0.94	medium, main peak is product, one small peak with 402.17656 (TMG-sideproduct) & one small peak with 469.09819 (bis-acylated product)	0.10	0.20	0.29	0.05	0.05	0.05	0.05	0.05	0.05	0.56	0.66	0.53	0.53	0.48	0.40	0.05	0.05	0.19	0.56	0.62	0.61	0.62	0.71	0.77	0.72	0.66	0.79

MAK-1-396	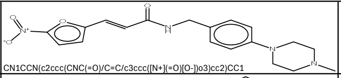 CN1CCN(c2ccc(CNC(=O)/C=C/c3ccc([N+](=O)[O-])o3)cc2)CC1	371.17138	371.17111	0.73	pure	0.05	0.05	0.05	0.05	0.05	0.05	0.05	0.05	0.05	0.35	0.55	0.48	0.10	0.09	0.16	0.05	0.05	0.05	0.62	0.74	0.66	0.66	0.73	0.84	0.68	0.74	0.84
MAK-1-397	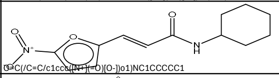 O=C(/C=C/c1ccc([N+](=O)[O-])o1)NC1CCCCC1	265.11828	265.11809	0.73	pure	0.33	0.26	0.44	0.23	0.05	0.06	0.05	0.05	0.05	0.57	0.69	0.56	0.69	0.55	0.51	0.25	0.20	0.30	0.66	0.73	0.70	0.72	0.75	0.85	0.68	0.71	0.85
MAK-1-398	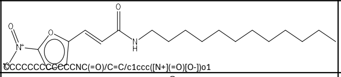 CCCCCCCCCCCCNC(=O)/C=C/c1ccc([N+](=O)[O-])o1	351.22783	351.22734	1.40	medium, medium product peak, one other medium peak with 284.30578	0.69	0.80	0.76	0.44	0.63	0.62	0.32	0.26	0.17	0.53	0.68	0.54	0.65	0.57	0.53	0.52	0.56	0.54	0.72	0.74	0.68	0.74	0.79	0.89	0.72	0.76	0.88
MAK-1-399	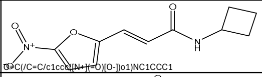 O=C(/C=C/c1ccc([N+](=O)[O-])o1)NC1CCC1	237.08698	237.08688	0.43	pure	0.36	0.38	0.29	0.19	0.07	0.05	0.05	0.05	0.05	0.51	0.62	0.53	0.28	0.16	0.21	0.05	0.05	0.05	0.76	0.84	0.77	0.75	0.78	0.63	0.77	0.77	0.81
MAK-1-400	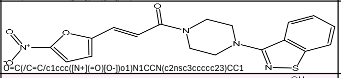 O=C(/C=C/c1ccc([N+](=O)[O-])o1)N1CCN(c2nsc3ccccc23)CC1	385.09650	385.09592	1.51	pure	0.30	0.44	0.50	0.37	0.50	0.49	0.50	0.45	0.54	0.54	0.62	0.53	0.68	0.55	0.59	0.63	0.65	0.64	0.76	0.81	0.80	0.79	0.84	0.63	0.77	0.85	0.93
MAK-1-401	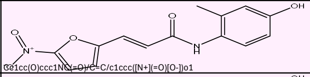 Cc1cc(O)ccc1NC(=O)/C=C/c1ccc([N+](=O)[O-])o1	289.08190	289.08170	0.68	high, big product peak and big peak with 387.16614 (TMG-sideproduct)	0.05	0.05	0.05	0.05	0.05	0.05	0.05	0.05	0.05	0.36	0.60	0.43	0.09	0.06	0.13	0.05	0.05	0.05	0.69	0.78	0.74	0.70	0.78	0.71	0.70	0.75	0.74
MAK-1-402	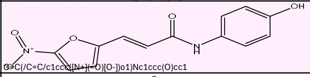 O=C(/C=C/c1ccc([N+](=O)[O-])o1)Nc1ccc(O)cc1	275.06625	275.06603	0.79	high, big product peak and two medium peaks with 373.15038 (TMG-sideproduct)	0.05	0.05	0.05	0.05	0.05	0.05	0.08	0.09	0.07	0.05	0.05	0.05	0.05	0.05	0.05	0.10	0.10	0.10	0.72	0.75	0.73	0.75	0.75	0.66	0.76	0.81	0.55
MAK-1-403	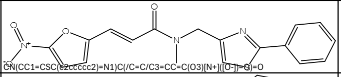 CN(CC1=CSC(c2ccccc2)=N1)C(/C=C/C3=CC=C(O3)[N+]([O-])=O)=O	370.08560	370.08520	1.09	pure	0.05	0.05	0.05	0.07	0.06	0.06	0.06	0.07	0.06	0.55	0.68	0.53	0.62	0.53	0.52	0.48	0.51	0.43	0.73	0.81	0.76	0.73	0.79	0.86	0.72	0.77	0.92
MAK-1-404	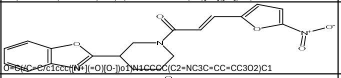 O=C(/C=C/c1ccc([N+](=O)[O-])o1)N1CCCC(C2=NC3C=CC=CC3O2)C1	368.12410	368.12369	1.10	pure	0.05	0.05	0.05	0.06	0.25	0.05	0.06	0.06	0.05	0.59	0.67	0.53	0.70	0.59	0.55	0.51	0.53	0.51	0.76	0.81	0.77	0.76	0.77	0.59	0.75	0.80	0.71
MAK-1-405	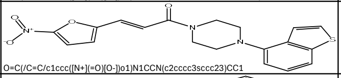 O=C(/C=C/c1ccc([N+](=O)[O-])o1)N1CCN(c2cccc3sccc23)CC1	384.10125	384.10075	1.31	pure	0.05	0.05	0.05	0.08	0.07	0.08	0.08	0.10	0.08	0.55	0.66	0.54	0.67	0.60	0.54	0.59	0.56	0.56	0.68	0.77	0.75	0.72	0.81	0.74	0.74	0.87	0.79
MAK-1-406	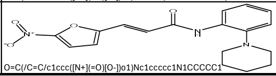 O=C(/C=C/c1ccc([N+](=O)[O-])o1)Nc1ccccc1N1CCCCC1	342.14483	342.14450	0.97	pure	0.71	0.83	0.70	0.66	0.62	0.58	0.48	0.46	0.78	0.56	0.67	0.55	0.71	0.61	0.54	0.70	0.67	0.65	0.73	0.81	0.73	0.74	0.86	0.98	0.76	0.81	1.06

Crude products that showed promising activity but were found to be impure by HPLC–MS were purified by preparative high-performance liquid chromatography (HPLC) prior to minimal inhibitory concentration (MIC) determination. In case of reactions where major amounts of side products were formed, the largest fraction obtained from preparative HPLC was assumed to be primarily responsible for the observed activity and thus selected for MIC determination. In all cases of diaminoalkane precursors, this was the bis-acylated product (**D2**, **D5**, **D6** and **D14**). For aromatic diamine and aminophenol precursors, tetramethylguanidinylation products were the most prevalent by-products, but they were never the dominant species. Nevertheless, the tetramethylguanidinylated version of **D7** (**D7-TMG**) was included in the MIC-assay to check whether it makes a relevant contribution to the activity.

Table S2: MICs of all purified and retested derivatives of the amide series.

**Compound**		**MICs** [µM]	
ID	Structure	*E. coli* K12	*S. aureus* USA300
8	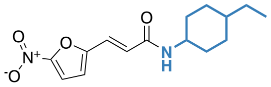	>200[a]	6.25
D1	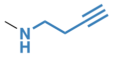	40.0	100[a]
D2	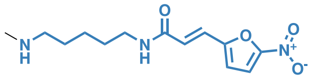	50.0	12.5
D3	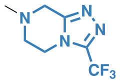	200	100[a]
D4	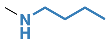	200	200[a]

Continued on next page

Compound (continued)		MICs [µM]	
ID	Structure	*E. coli*	*S. aureus*
D5	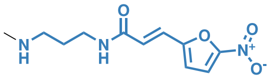	50.0	12.5
D6	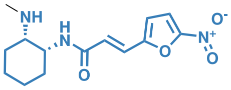	>200[b]	3.13
D7	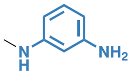	7.81	0.98
D7-TMG	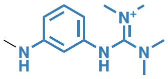	50.0	25.0
D8	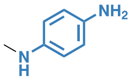	1.56	0.78
D9	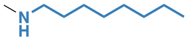	>200[a]	3.13
D10	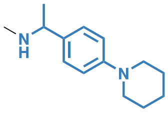	>200[a]	12.5
D11	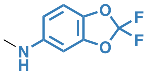	>200[b]	3.13
D12	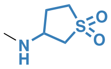	25.0	6.25
D13	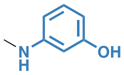	6.25	0.78
D14	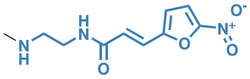	25.0	3.13
D15	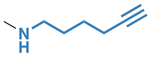	200	100[a]
D16	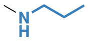	200	>200[a]

Continued on next page

Compound (continued)		MICs [µM]	
ID	Structure	*E. coli*	*S. aureus*
D17	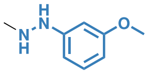	100	100[b]
D18	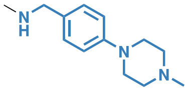	50.0	3.13
D19	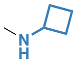	100	100[a]
D20	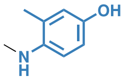	50.0	3.13
D21	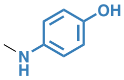	6.25	1.56
D22	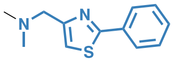	>200[a]	3.13
D23	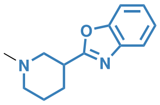	>200[a]	25.0
D24	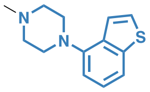	>200[a]	3.13

ESKAPEE Panel

Table S3: MICs of **D8** in type strains and clinical isolates of ESKAPEE pathogens.

**Organism**	**Strain**	**MIC** [µM]
Enterococcus faecium	DSM 20477	50
Staphylococcus aureus	USA300	0.78
Klebsiella pneumoniae	DSM 30104	3.1
Acinetobacter baumannii	DSM 30007	>200
Pseudomonas aeruginosa	PAO1	>200
Enterobacter cloacae	DSM 30054	100
Escherichia coli	K12	1.6
Escherichia coli	CFT073	6.3

Resistance Generation Assay by Serial Passage

Table S4: Resistance development of *E. coli* K12 during serial passaging in the presence of 0.25- to 4-fold MIC concentrations of **D8** and Nitrofurantoin (NFA) in 12-well plate format. The highest concentrations tested were 200 µM (128-fold MIC) for **D8** and 800 µM (32-fold MIC) for NFA, which represent the respective solubility limits. The table contains all individual data from $n = 3$ independent experiments. *The MICs on day 7, as well as the reference-MICs ("Day 0"), were performed in a different assay format (standard MIC procedure with serial dilution in 96-well plate).

Day	MIC of D8 [µM]			MIC of NFA [µM]		
	Replicate A	Replicate B	Replicate C	Replicate A	Replicate B	Replicate C
0*		1.6			50	
1	3.1	3.1	6.3	100	100	100
2	6.3	6.3	12.5	100	100	100
3	12.5	12.5	25	200	200	200
4	12.5	25	50	400	400	400
5	25	100	50	400	400	400
6	50	200	200	400	400	800
7*	>200	>200	>200	200	200	>800

Table S5: MICs of **D8** and NFA in *E. coli* K12 serially passaged with **D8**, NFA and DMSO for 6 days.

Passaged with	Replicate	MIC of D8 [µM]	MIC of NFA [µM]
**D8**	1	>200	800
	2	>200	100
	3	>200	200
NFA	1	12.5	200
	2	6.3	200
	3	>200	>800
DMSO	1	1.6	50
	2	3.1	50
	3	1.6	50

Toxicity Study (MTT assay)

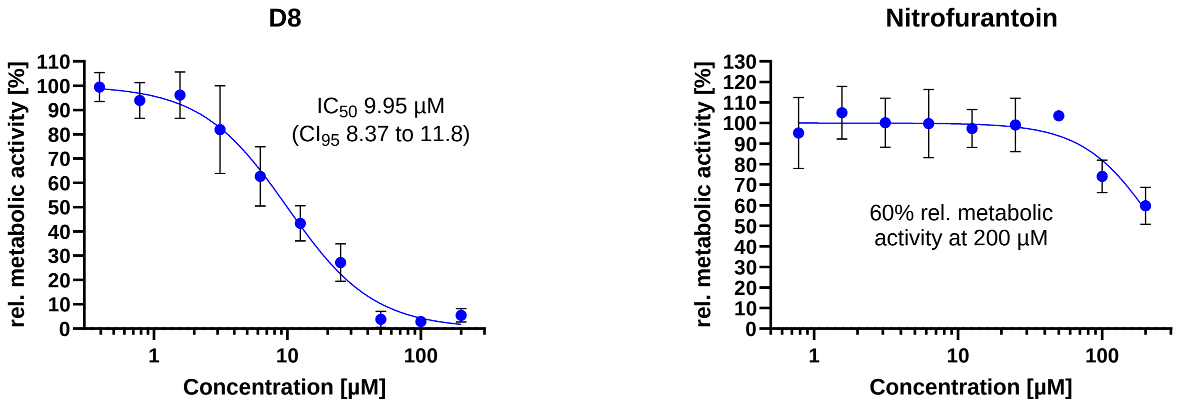

Figure S6: Relative metabolic activity of HEK 293 cells determined through an MTT assay after 24 h treatment with **D8** and NFA at concentrations up to 200 µM. Data represent mean ± std. of $n = 3$ biologically independent replicates, performed in technical triplicates each. IC_{50} values (concentration at which 50 % viability is reached) were determined using GraphPad Prism v10.0.1. Absorbance at 570 nm (formazan) and 630 nm (background) was measured and the background was subtracted, the absorption values of technical replicates were averaged and the resulting values were normalized to the upper and lower asymptote of a nonlinear fit of the data ([Inhibitor] vs. response – variable slope (four parameters)) for each compound tested, followed by re-fitting a nonlinear regression to the normalized data ([Inhibitor] vs. normalized response – variable slope) according to a protocol by Krebs *et al.*.[S4]

Cyclic Voltammetry Measurements

Cyclic voltammetry (CV) data were measured to compare the reduction peak potentials of the nitro groups of the active drug **D8**, its inactive derivative **D27** and nitrofurantoin, observed as the first reduction peak of these compounds. While compound **D8**, in which the molecule is fully conjugated through a central Michael system shows the reduction of its nitro group at a mild potential of −0.42 V, its inactive congener compound **D27** with interrupted π-conjugation has the nitro group reduction at more extreme potential of −0.68 V. The reduction potential of nitrofurantoin (−0.58 V) lies in between, which is in line with the antibiotic activities of the three compounds. This correlation emphasizes the dependence of the antibiotic activity on electron transfer at the nitro groups and the need for a reduction potential tailored to that of the bacterial nitroreductases. Further comparison to CV measurements of compounds **D25** and **D26**, which do not possess nitro groups, show their

first reductions at drastically more negative reduction peak potentials of −1.44 V and −1.28 V vs. Ag/AgCl, respectively; thus, further corroborating the first reductions of compounds **D8** and **D27** are nitro group centered.

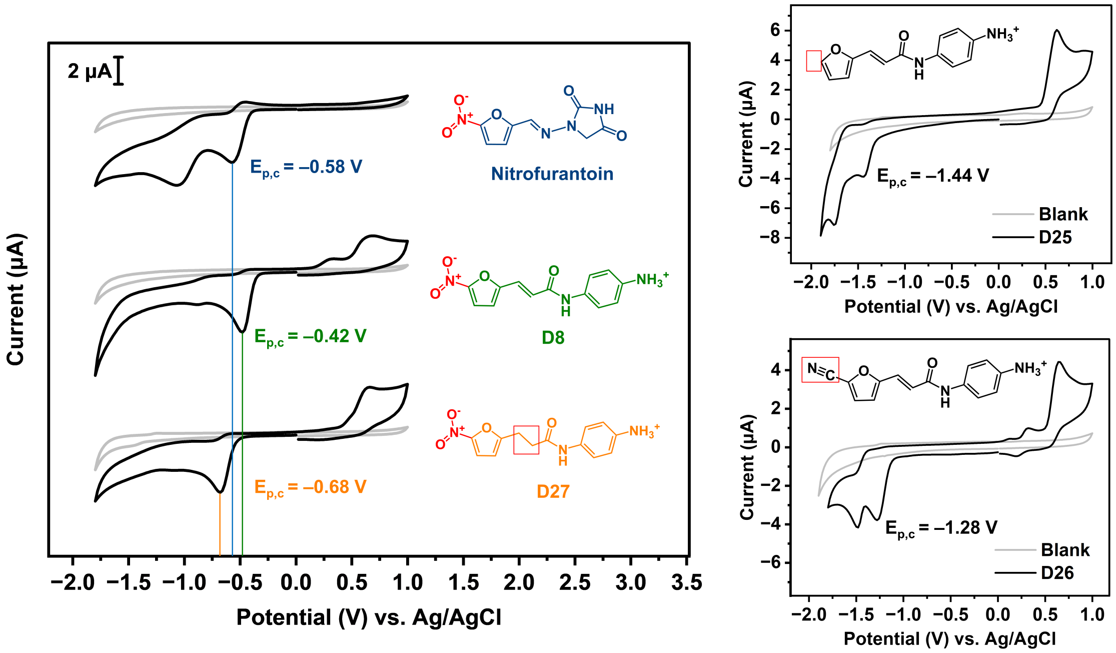

Figure S7: CV data of nitrofurantoin, **D8** and **D27**, as well as **D25** and **D26**, measured in 0.08 M aqueous Na_2SO_4 with 20 % DMF at a scan rate of 0.2 V/s, using a 1 mm GC disc, a Pt rod, and a leakless Ag/AgCl (3.4 M KCl) as working, counter, and reference electrodes, respectively. Data of all samples is presented together with the solvent backgrounds recorded before each measurement (grey traces in all figures). Modifications from the active derivative **D8** are highlighted in red boxes for each control sample.

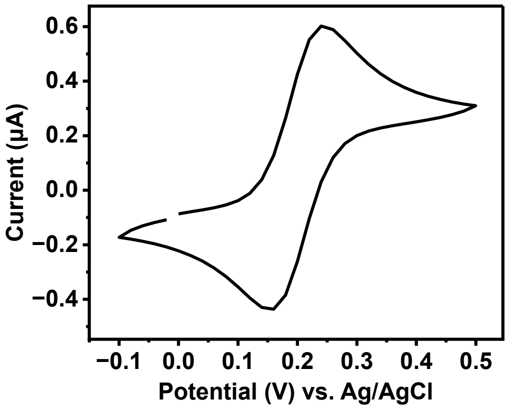

Figure S8: CV analysis of ferrocene for external calibration of the leakless Ag/AgCl (3.4 M KCl) reference electrode under the experimental conditions used herein (0.08 M aqueous Na_2SO_4 with 20 % DMF). The half-wave potential of the Fc^+/Fc couple was measured at +0.20 V vs. Ag/AgCl.

Methods

Structure Generation and Curation

Generation

We retrieved all antibiotic scaffolds from PubChem searching for "antibiotic" (23.01.2024) yielding 2239 entries.[S5] As this set of antibiotics is biased towards well-researched classes of antibiotics, where many derivatives are known, the dataset needs to be refined. This was done to avoid new *de novo* compounds being biased towards one well-researched scaffold. Therefore, we selected 131 scaffolds that were distant from each other in terms of Tanimoto similarity, resulting in a set of compounds each representing distinct structural classes. We applied the "virtual libraries" (Commit `694824c`) CLM by Moret et al. to generate new scaffolds.[S6] Molecules across all epochs were pooled and used for further analysis.

Curation

Retrosynthetic Accessibility Thakkar et al. introduced a computer-aided synthesis planning method capable of classifying whether a synthetic route can be identified by AiZynthFinder.[S7,S8] We used an ensemble prediction of `RAScorerNN` and `RAScorerXGB` to filter out difficult to synthesise compounds.

Weighted Accumulation Prediction To achieve best possible accumulation prediction into Gram-negative cells we applied two ML models. The first is an AutoML model trained on accumulation data generated by Richter et al.[S2] AutoGluon[S9] was selected for its ability to achieve optimal prediction performance through its self-assessment process. Molecules were represented as ECFPs through RDKit.[S10] Here we chose PR AUC as evaluation metric. Secondly, TwinBooster[S1] a text-based zero-shot molecular property prediction method was applied. The text prompt can be found above. Finally, we calculated the weighted

accumulation prediction $\widehat{y_a}$ using the formula

$$\widehat{y_a} = s \cdot \hat{y}_{\text{eNTRy}} + (1 - s) \cdot \hat{y}_{\text{TwinBooster}},$$

where s is the coefficient reflecting the Tanimoto similarity to the eNTRy training data, $\hat{y}_{\text{eNTRy}}$ is the eNTRy prediction of the AutoML model, and $\hat{y}_{\text{TwinBooster}}$ is the prediction from the zero-shot prediction model.

SAR We switched from zero-shot predictions to only structural and physicochemical features because the SAR derivatives have a high structural similarity. The ensemble mean was used for derivative prediction and ranking. We used the `MordredFingerprint` and `PhysiochemicalPropertiesFingerprint` as physiochemical features implemented by `scikit-fingerprints`. One AutoML model per feature type was trained according to the procedure described above.

Visualization Uniform manifold approximation and projections (UMAPs) where generated based on ECFPs. If contour maps are shown, we used SciPy `griddata` to generate them.

Chemistry

General Considerations

All reactions for which an argon atmosphere is stated in the experimental procedure were performed using standard *Schlenk* technique and Argon 4.6 from Westfalen AG, unless otherwise noted. Glassware was equipped with magnetic stirring and dried *in vacuo* using a heat gun. To transfer dry solvents or reagents, syringes were used, which were purged three times with argon prior to use. Room temperature (RT) is defined as 20-25 °C. All performed reactions were monitored by HPLC–MS, nuclear magnetic resonance (NMR) spectroscopy or analytic thin-layer chromatography (TLC) to examine the reaction progress. Unless otherwise indicated, all chemicals were obtained from commercial sources and were used without any further purification. After purification by flash column chromatography (FCC), products were concentrated using a rotary evaporator. After purification by preparative HPLC, products were concentrated using a lyophilizer.

Solvents For setting up reactions, dry organic solvents stored under inert gas from commercial sources (Sigma-Aldrich Co. LLC, Thermo Fisher Scientific Inc., VWR International LLC) were used. For extractions and purification purposes, acetone, dichloromethane (DCM), diethyl ether (Et_2O), ethyl acetate (EtOAc), *n*-pentane (Pent), *n*-hexane (Hex) and methanol (MeOH) were used in technical grade from commercial sources. For analytical and preparative HPLC, ACN and trifluoroacetic acid (TFA) of HPLC-grade purity (Fisher Scientific Inc.), as well as double distilled water (ddH_2O) was used. ddH_2O was prepared from deionized water using a SARTORIUS Arium® Pro ultrapure water system.

Reagents All chemicals were purchased from commercial sources (Sigma-Aldrich Co. LLC, BLD Pharmatech Ltd., Thermo Fisher Scientific Inc., Merck KGaA, ABCR GmbH & Co.KG, TCI Europe GmbH) and were used without further purification unless otherwise stated.

Potassium azodicarboxylate was prepared in-house from azodicarbonamide by basic

hydrolysis in a 40 % aqueous KOH solution at 0 °C for 3 h. After filtration, washing of the solid with cold MeOH and drying, the reagent was stored at −20 °C for up to 2 months.[S11]

Chromatography Thin layer chromatography (TLC) was conducted using Merck silica gel 60 F254 plates. To visualize the spots of the different products, the TLC plates were exposed to UV light (wavelength $\lambda = 254$ nm) and stained with $KMnO_4$-solution (1.5 g $KMnO_4$, 10 g K_2CO_3 and 0.125 g NaOH in 200 mL water), followed by heating with a heat gun if necessary.

Flash column chromatography was performed with silica gel from Merck KGaA (SiO_2, 60 Å, 40-63 µm) as stationary phase and forced flow of the eluent, using the gradient stated in the experimental procedure.

Automated flash column chromatography was performed on a flash column chromatography system (BÜCHI Reveleris X2) by employing silica gel from Merck KGaA (SiO_2, 60 Å, 40-63 µm) as stationary phase and forced flow of the eluent, using the gradient stated in the experimental procedure.

Preparative HPLCs was performed using a WATERS 2545 quaternary gradient module equipped with a fraction collector. The separation was performed on a YMC Triart C18 column (250 × 10 mm, 5 µm) with mobile phase A (0.1 % TFA in ddH_2O) and mobile phase B (0.1 % TFA in ACN). The specific gradient used is defined in each procedure.

Analytics

NMR Spectroscopy NMR spectra were recorded on BRUKER AV-HD400 and AV-HD500 spectrometers as solutions in $CDCl_3$ and DMSO-d_6 at 300 K. The signals refer to the residual proton signals of the deuterated solvent:

- $CDCl_3$: $\delta(^1H) = 7.26$ ppm, $\delta(^{13}C) = 77.16$ ppm
- DMSO-d_6: $\delta(^1H) = 2.50$ ppm, $\delta(^{13}C) = 39.52$ ppm

All chemical shifts are reported as δ-values in parts per million (ppm). All coupling constants are reported in Hertz (Hz). For the characterization of the observed signal

multiplicities the following abbreviations were used: s (singulet), d (doublet), t (triplet), q (quartet), quint (quintet), q (quartet), dd (doublet of doublet), dt (doublet of triplet), td (triplet of doublet), tt (triplet of triplet) and m (multiplet). The NMR spectra were processed using MestReNova v.12.0.2-20910 (Mestrelab Research S.L.).

High-Performance Liquid Chromatography–Mass Spectrometry (HPLC–MS) Low-Resolution Mass Spectra (LRMS) with Electrospray Ionization (ESI) were recorded on an MSQ™ Plus coupled to a DIONEX Ultimate 3000 HPLC system (Thermo Fisher Scientific Inc.). Electrospray Ionization High-Resolution Mass Spectrometry (ESI-HRMS) was performed on an LTQ FT Ultra™ or a Q Exactive™ Plus mass spectrometer (both Thermo Fisher Scientific Inc.), coupled to a DIONEX Ultimate 3000 HPLC system (Thermo Fisher Scientific Inc.). MS data was evaluated with THERMO Xcalibur 2.1 (Thermo Fisher Scientific Inc.).

Gas Chromatography–Mass Spectrometry (GC–MS) Low-Resolution Mass Spectra (LRMS) with Electron Ionization (EI) were recorded on a GC–MS system by Agilent, consisting of an Agilent GC 7890B gas chromatograph and single quadrupole mass detector MS 5977A, equipped with a 7693A automatic liquid sampler with G4513A autoinjector. A HP-5MS UI column (length: 30m, internal diameter: 0.25mm, film: 0.25µm) was used for separation with a gradient from 60 to 300 °C. MS data was aquired and evaluated using Agilent MassHunter B.07.03.2129.

Synthesis Procedures

Compound 1

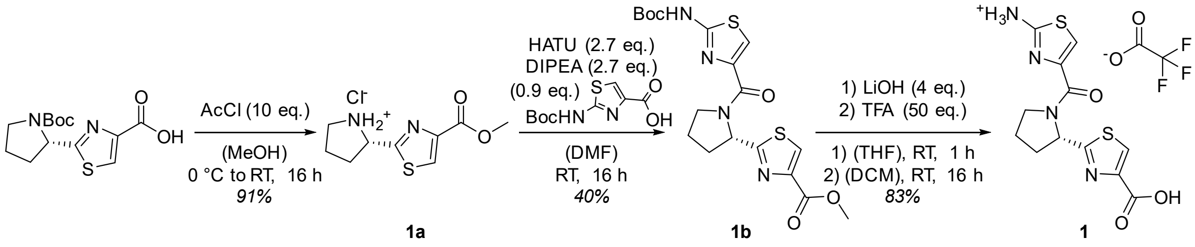

Figure S9: Synthesis overview of compound **1**.

Methyl (*S*)-2-(pyrrolidin-2-yl)thiazole-4-carboxylate (1a): To a solution of (*S*)-2-(1-(*tert*-butoxycarbonyl)pyrrolidin-2-yl)thiazole-4-carboxylic acid (20.0 mg, 66.4 µmol, 1.0 eq.) in methanol (0.5 mL) was added acetyl chloride (40.1 µL, 664 µmol, 10.0 eq.) dropwise at 0 °C. The reaction mixture was stirred overnight at RT. After completion, the solvent was removed *in vacuo* to give the hydrochloride salt of **1a** (15 mg, 88 %) as colorless crystals.

Mass spectrometry (ESI-LRMS, positive): m/z: $[M+H]^+$ calcd. for $C_9H_{13}N_2O_2S^+$: 213.07; found: 213.21.

Methyl (*S*)-2-(1-(2-((tert-butoxycarbonyl)amino)thiazole-4-carbonyl)pyrrolidin-2-yl)thiazole-4-carboxylate (1b): To a solution of **1a** (15.0 mg, 60.5 µmol, 1.1 eq.), 2-Boc-aminothiazole-4-carboxylic acid (13.7 mg, 55.0 µmol, 1.0 eq.), and HATU (62.7 mg, 165 µmol, 3.0 eq.) in *N N*-dimethylformamide (DMF) (0.5 mL) was added DIPEA (28.7 µL, 165 µmol, 3.0 eq.) dropwise. The reaction mixture was stirred at RT overnight. The mixture was diluted with EtOAc and a saturated aqueous $NaHCO_3$-solution was added. The phases were separated, and the aqueous layer was extracted with EtOAc (3×). The combined organic layers were washed with 5 % aq. LiCl (5×), brine, dried over $MgSO_4$, and concentrated *in vacuo*. The crude product was purified by preparative HPLC (30–65 % ACN in water with 0.1 %(v/v) TFA), and lyophilization afforded the TFA salt of **1b** (12 mg, 40 %) as a white solid.

^{1}H-NMR (400 MHz, $CDCl_3$): δ [ppm] = 8.07 (s, 0.4H), 8.01 (s, 0.6H), 7.74 (s, 0.6H), 7.65 (s, 0.4H), 6.45 (t, J = 4.7 Hz, 0.6H), 5.67 (t, J = 6.2 Hz, 0.4H), 4.26–3.91 (m, 0.4H), 3.95 (s, 1.8H), 3.93 (s, 1.2H), 3.96–3.78 (m, 0.6H), 2.48–2.32 (m, 2H), 2.19–1.95 (m, 0.8H), 2.06–1.78 (m, 1.2H), 1.55 (s, 3.6H), 1.50 (s, 5.4H). ***Note:*** *The product consists of two rotamers in a ratio of 60:40 at RT. The tabulated NMR data reflects this through the reported integral values.*

^{13}C-NMR (126 MHz, $CDCl_3$): δ [ppm] = 177.85, 173.82, 162.61, 162.02, 161.98, 161.68, 161.01, 160.70, 158.84, 158.45, 152.25, 147.06, 146.55, 144.99, 144.54, 129.00, 127.77, 127.19, 127.06, 121.05, 119.89, 83.34, 82.96, 77.41, 77.16, 76.91, 61.42, 59.85, 52.64, 52.57, 49.95, 47.99, 34.95, 31.85, 30.29, 29.83, 28.26, 28.24, 25.28, 21.20. ***Note:*** *The two rotamers cause doubling of signals for most ^{13}C nuclei.*

Mass spectrometry (ESI-HRMS, positive): m/z: $[M+H]^+$ calcd. for $C_{18}H_{23}N_4O_5S_2^+$: 439.11044; found: 439.10983. $[M+Na]^+$ calcd. for $C_{18}H_{22}N_4NaO_5S_2^+$: 461.09238; found: 461.09168.

(*S*)-2-(1-(2-Aminothiazole-4-carbonyl)pyrrolidin-2-yl)thiazole-4-carboxylic acid, 2,2,2-trifluoroacetate salt (1): To a solution of **1b** (12.0 mg, 21.7 µmol, 1.0 eq.) in tetrahydrofuran (THF) (0.2 mL) was added lithium hydroxide (2.0 mg, 84 µmol, 3.9 eq.) dissolved in 0.1 mL water. The reaction mixture was stirred at RT until HPLC–MS showed complete conversion to the carboxylic acid (1 h). Subsequently, 100 µL of 1 M HCl was added to the reaction mixture and thoroughly mixed. The phases were separated, and the aqueous phase was extracted with DCM (3×). The combined organic phases were washed with brine, dried over $MgSO_4$, and concentrated to approximately 0.2 mL. To this solution, trifluoroacetic acid (124 mg, 1.09 mmol, 50 eq.) was added. The mixture was stirred at RT overnight. The solvent mixture (DCM and TFA) was then evaporated, and the crude product was purified by preparative HPLC (2–40 % ACN in water with 0.1 %(v/v) TFA). Lyophilization of the product fractions afforded the TFA salt of **1** (12 mg, 83 %) as a white solid.

^{1}**H-NMR** (500 MHz, MeOD): δ [ppm] = 8.28 (s, 1H), 7.50 (s, 0.75H), 7.14 (s, 0.25H), 6.15 (s, 0.25H), 5.56 (s, 0.75H), 4.04 (d, J = 80.3 Hz, 1.5H), 3.83 (d, J = 36.0 Hz, 0.5H), 2.57–2.24 (m, 0.5H), 2.47–2.17 (m, 1.5H), 2.24–2.03 (m, 1.5H), 2.10–1.95 (m, 0.5H). ***Note:*** *The product consists of two rotamers in a ratio of 75:25 at RT. The tabulated NMR data reflects this through the reported integral values.*

^{13}**C-NMR** (126 MHz, MeOD): δ [ppm] = 176.36, 174.70, 170.76, 170.37, 163.95, 162.27, 161.92, 161.63, 160.32, 148.69, 148.12, 137.86, 129.17, 129.07, 118.70, 117.17, 116.39, 114.91, 114.82, 114.49, 114.09, 62.13, 61.34, 50.74, 49.63, 49.51, 49.34, 49.17, 49.00, 48.83, 48.66, 48.49, 35.63, 33.24, 25.99, 22.39. ***Note:*** *The two rotamers cause doubling of signals for most* ^{13}C *nuclei.*

Mass spectrometry (ESI-HRMS, positive): m/z: $[M+H]^+$ calcd. for $C_{12}H_{13}N_4O_3S_2^+$ 325.04236; found: 325.04228. (ESI-HRMS, negative): m/z: $[M-H]^-$ calcd. for $C_{12}H_{11}N_4O_3S_2^-$ 323.02781; found: 323.02714.

Compound 2

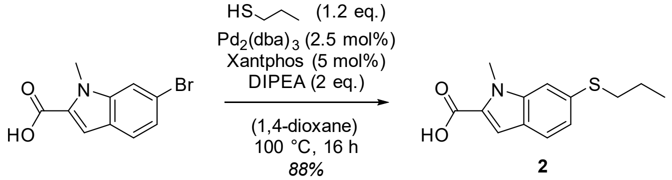

Figure S10: Synthesis overview of compound **2**.

1-Methyl-6-(propylthio)-1*H*-indole-2-carboxylic acid (2): 6-Bromo-1-methyl-1*H*-indole-2-carboxylic acid (130 mg, 0.50 mmol, 1.0 eq.) and DIPEA (174 µL, 1.00 mmol, 2.0 eq.) were dissolved in dry 1,4-dioxane (2.00 mL). The flask was evacuated until the solvent started boiling and then backfilled with argon (3 cycles). Subsequently, 1-propanethiol (54.4 µL, 0.601 mmol, 1.2 eq.), Xantphos (15 mg, 0.025 mmol, 0.05 eq.), and $Pd_2(dba)_3$ (11.8 mg, 0.0125 mmol, 0.025 eq.) were added, and the mixture was degassed twice more. The reaction was heated to

reflux (100 °C) overnight. The mixture was allowed to reach ambient temperature, filtered, and concentrated *in vacuo*. The crude product was purified by FCC (DCM/MeOH = 99:1 → 90:10), yielding **2** (110 mg, 88 %) as a yellow solid. A small batch for analytics and biological testing was additionally purified by preparative HPLC (30–75 % ACN in water with 0.1 %(v/v) TFA).

TLC (DCM/MeOH = 92:8): R_f = 0.5 (UV, $KMnO_4$)

^{1}H-NMR (500 MHz, $CDCl_3$): δ [ppm] = 7.59 (d, J = 8.5 Hz, 1H), 7.40 (s, 1H), 7.36 (d, J = 1.5 Hz, 1H), 7.16 (dd, J = 8.4 Hz, 1.5 Hz, 1H), 4.11–4.01 (m, 3H), 2.98 (t, J = 7.3 Hz, 2H), 1.77–1.64 (m, 2H), 1.05 (t, J = 7.3 Hz, 3H).

^{13}C-NMR (101 MHz, $CDCl_3$): δ [ppm] = 140.7, 134.6, 127.0, 124.4, 123.1, 123.1, 112.5, 111.0, 77.5, 77.2, 76.8, 36.7, 31.9, 22.7, 13.6.

Mass spectrometry (ESI-HRMS, negative): m/z: $[M-H]^-$ calcd. for $C_{13}H_{14}NO_2S^-$: 248.07507; found: 248.07431.

Compound 3

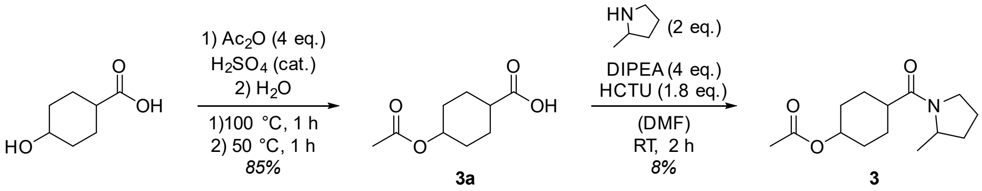

Figure S11: Synthesis overview of compound **3**.

4-Acetoxycyclohexane-1-carboxylic acid (3a): To 4-hydroxycyclohexanecarboxylic acid (433 mg, 3.00 mmol, 1.0 eq.) was added acetic anhydride (1.13 mL, 12.0 mmol, 4.0 eq.), followed by the addition of 4 drops of conc. sulfuric acid. The reaction mixture was heated to 100 °C and stirred for 1 h. The brown oil was then concentrated *in vacuo*. Water (20 mL) was added, and the suspension was stirred for 1 h at 50 °C. A sat. aq. $NaHCO_3$-solution (75 mL) was added until a basic pH was reached. The solution was washed with EtOAc (40 mL). The

aq. phase was acidified with conc. HCl-solution to around pH = 2 and then extracted with EtOAc (3 × 40 mL). The combined organic phases of the acidic extraction were dried over $MgSO_4$, and the solvent removed *via* rotary evaporation. The reaction yielded crude **3a**, which was used for the next reaction without further purification.

Mass spectrometry (ESI-LRMS, negative): m/z: $[M\text{-}H]^-$ calcd. for $C_9H_{13}O_4^-$: 185.08; found: 185.03.

4-(2-Methylpyrrolidine-1-carbonyl)cyclohexyl acetate (3): Crude **115a** (559 mg, 3.00 mmol, 1.0 eq.) was dissolved in DMF (20 mL). DIPEA (2.09 mL, 12.0 mmol, 4.0 eq.) and HCTU (2.23 g, 5.40 mmol, 1.8 eq.) were added and the solution was stirred for 15 min at RT. Subsequently, 2-methylpyrrolidine (0.58 mL, 6.00 mmol, 2.0 eq.) was added and the solution was stirred for 2 h at RT. The reaction was then quenched with an aq. 2 M HCl-solution (20 mL) and extracted with EtOAc (3 × 20 mL). The combined organic phases were washed with a sat. aq. $NaHCO_3$-solution, brine, a 5 wt % aq. LiCl-solution (3 × 20 mL), brine, and dried over $MgSO_4$. After removing the solvent *in vacuo*, the crude product was purified *via* FCC (Pent/EtOAc = 80:20 → 70:30 → 60:40 → 50:50 → 20:80). The reaction yielded **3** (60.0 mg, 8 %) as yellow crystals.

TLC (Pent/EtOAc = 80:20): R_f = 0.09 ($KMnO_4$)

^{1}H-NMR (400 MHz, $CDCl_3$): δ [ppm] = 4.14–3.91 (m, 1H), 3.57–3.38 (m, 2H), 3.31–3.22 (m, 1H), 2.47–2.29 (m, 1H), 2.05–1.95 (m, 3H), 1.90 (tt, J = 12.0 Hz, 6.3 Hz, 3H), 1.84–1.67 (m, 3H), 1.66–1.53 (m, 3H), 1.52–1.31 (m, 3H), 1.15–1.00 (m, 3H).

^{13}C-NMR (101 MHz, DMSO-d_6): δ [ppm] = 172.7, 169.8, 72.1, 68.6, 51.9, 46.0, 31.3, 28.6, 23.6, 22.8, 21.1, 19.4.

Mass spectrometry (ESI-HRMS, positive): m/z: $[M+H]^+$ calcd. for $C_{14}H_{24}NO_3^+$: 254.17507; found: 254.17517.

Compound 4

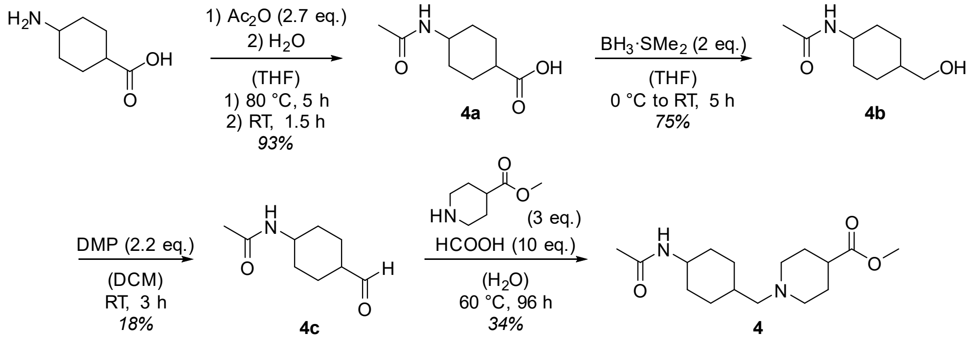

Figure S12: Synthesis overview of compound **4**.

4-Acetamidocyclohexane-1-carboxylic acid (4a): To a suspension of 4-aminocyclohexanecarboxylic acid (1.06 g, 7.00 mmol, 1.0 eq.) in THF (20 mL) was added acetic anhydride (1.79 mL, 18.9 mmol, 2.7 eq.). The mixture was stirred for 5 h at 75 °C. After complete consumption of the starting material, the solution was allowed to reach RT and the solvent was removed *in vacuo*. To hydrolyze the resulting carboxylic anhydride, water (20 mL) was added, and the suspension was stirred for 1.5 h at RT. The solution was then lyophilized to give **4a** (1.20 g, 93 %) as a colorless solid.

^{1}H-NMR (500 MHz, $CDCl_3$): δ [ppm] = 3.92 (m, 1H), 2.53 (td, J = 6.7 Hz, 3.4 Hz, 1H), 1.98 (s, 3H), 1.93–1.82 (m, 1H), 1.79–1.64 (m, 4H), 1.61–1.48 (m, 3H).

***N*-(4-(Hydroxymethyl)cyclohexyl)acetamide (4b):** A solution of **4a** (556 mg, 3.00 mmol, 1.0 eq.) in THF (10 mL) under an argon atmosphere was cooled to 0 °C and $BH_3{\cdot}SMe_2$ (0.60 mL, 6.00 mmol, 2.0 eq.) was added. The reaction mixture was stirred for 2 h at 0 °C. The reaction was allowed to reach RT and stirred for another 3 h. After a reaction control (mass spectrometry) revealed remaining **4a**, more $BH_3{\cdot}SMe_2$ (0.30 mL, 3.00 mmol, 1.0 eq.) was added and the reaction was stirred for 1 h. The reaction was then quenched with a sat. aq. Na_2CO_3-solution (20 mL). The organic phase was separated, and the aq. phase was extracted with EtOAc (2 × 20 mL) and Et_2O (20 mL). The combined organic phases

were dried over Na_2SO_4, and the solvent removed *in vacuo*. The reaction yielded **4b** (384 mg, 75 %) as a colorless oil.

^{1}H-NMR (500 MHz, $CDCl_3$): δ [ppm] = 3.53–3.49 (m, 1H), 3.47–3.45 (m, 1H), 3.44–3.38 (m, 1H), 1.95 (s, 3H), 1.85–1.78 (m, 1H), 1.70–1.53 (m, 4H), 1.35–1.19 (m, 2H), 1.16–1.03 (m, 2H).

***N*-(4-Formylcyclohexyl)acetamide (4c):** **4b** (217 mg, 1.27 mmol, 1.0 eq.) was dissolved in DCM (10 mL) under an argon atmosphere and cooled to 0 °C. To the suspension was added Dess–Martin periodinane (700 mg, 1.10 mmol, 1.1 eq.) and it was stirred for 10 min at 0 °C and then 3 h at RT. A 10 wt % aq. $Na_2S_2O_3$-solution (10 mL) and a sat. aq. $NaHCO_3$-solution (5 mL) were added, and the organic phase was quickly extracted with DCM (1 × 15 mL). The combined organic phases were washed with brine, dried over Na_2SO_4 and concentrated *in vacuo*. The crude product was purified *via* preparative HPLC (2–50 % ACN in water with 0.1 %(v/v) TFA). The combined fractions of the pure product were lyophilized to give **4c** (44.9 mg, 18 %) as a colorless oil.

^{1}H-NMR (500 MHz, $CDCl_3$): δ [ppm] = 9.67 (s, 1H), 3.91–3.80 (m, 1H), 2.41 (quint, J = 5.0 Hz, 1H), 2.05–1.97 (m, 1H), 1.95 (s, 3H), 1.85–1.57 (m, 4H), 1.47–1.06 (m, 3H).

Methyl 1-((4-acetamidocyclohexyl)methyl)piperidine-4-carboxylate (4): To a solution of **4c** (29.9 mg, 0.18 mmol, 1.0 eq.) in water (2 mL) was added formic acid (0.03 mL, 0.90 mmol, 5.0 eq.). To this was added methyl 4-piperidinecarboxylate (0.04 mL, 0.27 mmol, 1.5 eq.) and the solution was stirred at 60 °C overnight. Additional amounts of formic acid (0.03 mL, 0.90 mmol, 5.0 eq.) and methyl 4-piperidinecarboxylate (0.04 mL, 0.27 mmol, 1.5 eq.) were added and the solution was stirred at 60 °C over 96 h. The solvent was removed *in vacuo* and the crude product was purified *via* preparative HPLC (2–25 % ACN in water with 0.1 %(v/v) TFA). The combined fractions of the pure product were lyophilized to give the TFA salt of **4** (25.4 mg, 34 %) as a colorless solid.

^{1}H-NMR (500 MHz, DMSO-d_6): δ [ppm] = 3.65 (d, J = 15.3 Hz, 3H), 3.57–3.30 (m,

3H), 3.02–2.82 (m, 4H), 2.62 (tt, J = 12.4 Hz, 3.8 Hz, 1H), 2.11–1.99 (m, 2H), 1.89–1.77 (m, 5H), 1.76 (s, 3H), 1.59–1.31 (m, 2H), 1.21–1.08 (m, 2H), 1.07–0.94 (m, 2H).

^{13}C-NMR (101 MHz, DMSO-d$_6$): δ [ppm] = 173.4, 168.2, 61.9, 51.7, 51.6, 47.2, 37.7, 31.4, 31.4, 29.1, 25.1, 22.7.

Mass spectrometry (ESI-HRMS, positive): m/z: $[M+H]^+$ calcd. for $C_{16}H_{29}N_2O_3^+$: 297.21727; found: 297.21668.

Compound 5

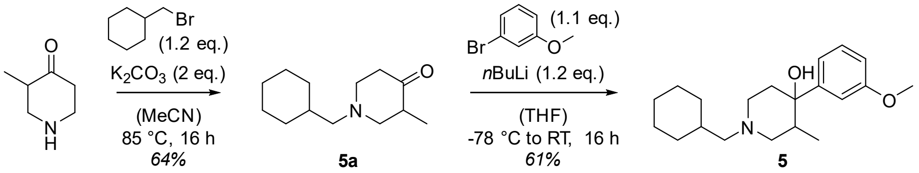

Figure S13: Synthesis overview of compound **5**.

1-(Cyclohexylmethyl)-3-methylpiperidin-4-one (5a): (Bromomethyl)cyclohexane (532 mg, 3.00 mmol, 1.0 eq.) was dissolved in ACN (15 mL) and K_2CO_3 (829 mg, 6.00 mmol, 2.0 eq.) and 3-methylpiperidin-4-one (420 mg, 3.60 mmol, 1.2 eq.) were added. The reaction mixture was heated to reflux (85 °C) overnight. After completion, the mixture was filtered and the solvent was removed *in vacuo*. The crude product was purified by FCC (Pent/EtOAc = 10:0 → 9:1 → 8:2), yielding **5a** (403 mg, 64 %) as a colorless solid.

TLC (Pent/EtOAc = 8:2): R_f = 0.57 ($KMnO_4$)

^{1}H-NMR (400 MHz, $CDCl_3$): δ [ppm] = 3.10–2.97 (m, 2H), 2.70–2.47 (m, 2H), 2.40–2.27 (m, 2H), 2.25–2.14 (m, 2H), 2.03 (t, J = 11.1 Hz, 1H), 1.85–1.76 (m, 2H), 1.76–1.64 (m, 3H), 1.54–1.40 (m, 1H), 1.30–1.10 (m, 3H), 1.00 (d, J = 6.6 Hz, 3H), 0.95–0.82 (m, 2H).

^{13}C-NMR (101 MHz, $CDCl_3$): δ [ppm] = 211.7, 77.5, 77.2, 76.8, 64.3, 61.7, 54.6, 44.4, 41.1, 36.0, 32.0, 31.9, 26.9, 26.3, 12.1.

Mass spectrometry (ESI-HRMS, positive): m/z: $[M+H]^+$ calcd. for $C_{13}H_{24}NO^+$:

210.18524; found: 210.18520.

1-(Cyclohexylmethyl)-4-(3-methoxyphenyl)-3-methylpiperidin-4-ol (5): To a solution of 1-bromo-3-methoxybenzene (103 mg, 0.55 mmol, 1.1 eq.) in THF (6 mL) at −78 °C under an argon atmosphere was added *n*-BuLi (240 µL, 0.60 mmol, 1.2 eq., 2.5 M in hexanes) dropwise over 10 min. The resulting slurry was stirred at −78 °C for 1 h. To the mixture was then added a solution of **5a** (105 mg, 0.50 mmol, 1.0 eq.) in THF (1 mL). The reaction mixture was stirred at −78 °C for 2 h and then warmed to RT and stirred for an additional 13 h. The mixture was poured over ice-water, and the aqueous phase was extracted with EtOAc. The combined organic phases were dried over $MgSO_4$ and concentrated *in vacuo*. The crude residue was purified by FCC (DCM/MeOH = 95:5) on silica, which was pre-treated with 2 % NEt_3 in DCM and washed with 2 column volumes of DCM prior to loading of the compound. The reaction yielded **5** (131 mg, 61 %) as a solid. As the NMR spectrum showed slight impurities, a small batch for biological testing was additionally purified by preparative HPLC (20–60 % ACN in water with 0.1 %(v/v) TFA), affording the compound as a TFA salt.

TLC (DCM/MeOH = 9:1): R_f = 0.42 ($KMnO_4$)

^{1}H-NMR (free amine, 400 MHz, $CDCl_3$): δ [ppm] = 7.25 (t, J = 7.9 Hz, 1H), 7.07–7.02 (m, 2H), 6.77 (dd, J = 8.1 Hz, 2.5 Hz, 1H), 3.81 (s, 3H), 2.81–2.71 (m, 1H), 2.71–2.64 (m, 1H), 2.36–1.99 (m, 6H), 1.87–1.77 (m, 3H), 1.77–1.60 (m, 6H), 1.60–1.45 (m, 1H), 1.32–1.10 (m, 4H), 1.04–0.74 (m, 3H), 0.62 (d, J = 6.8 Hz, 3H).

^{13}C-NMR (free amine, 101 MHz, $CDCl_3$): δ [ppm] = 159.75, 149.41, 129.27, 129.21, 117.32, 111.87, 111.02, 77.48, 77.16, 76.84, 74.30, 66.06, 57.58, 55.35, 50.06, 40.77, 39.27, 35.41, 32.27, 32.23, 26.97, 26.36, 12.50.

^{1}H-NMR (TFA salt, 500 MHz, $CDCl_3$): δ [ppm] = 10.63 (s, 1H), 7.29 (t, J = 7.9 Hz, 1H), 6.97–6.91 (m, 2H), 6.83 (dd, J = 8.2 Hz, 2.5 Hz, 1H), 4.98 (s, 1H), 3.83–3.78 (m, 3H), 3.59–3.49 (m, 1H), 3.45–3.36 (m, 1H), 3.23–3.12 (m, 1H), 2.96–2.85 (m, 3H), 2.78–2.69 (m,

1H), 2.68–2.57 (m, 1H), 1.91–1.73 (m, 6H), 1.68 (dt, J = 12.5 Hz, 3.6 Hz, 1H), 1.34–1.11 (m, 3H), 1.11–0.98 (m, 2H), 0.68 (d, J = 6.8 Hz, 3H).

Mass spectrometry (ESI-HRMS, positive): m/z: $[M+H]^+$ calcd. for $C_{20}H_{32}NO_2^+$: 318.24276; found: 318.24256.

Compound 6

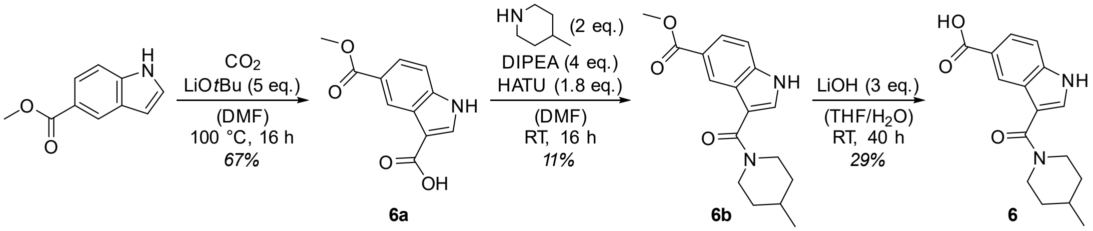

Figure S14: Synthesis overview of compound **6**.

5-(Methoxycarbonyl)-1*H*-indole-3-carboxylic acid (6a): A two-necked flask with a condenser and a septum was charged with lithium *tert*-butoxide (4.80 g, 60.0 mmol, 5.0 eq.) and methyl-5-indolecarboxylate (2.10 g, 12.0 mmol, 1.0 eq.). The flask was then evacuated. A Schlenk flask was filled with dry ice and equipped with a hose attached to a cannula. The cannula was inserted into the septum and a continuous flow of CO_2 gas was achieved using a bleed needle on top of the condenser. DMF (100 mL) was added to dissolve the reactants, and the reaction mixture was vigorously stirred at 100 °C for 4 h. The suspension was cooled to RT, quenched with an aq. 2 M HCl-solution (100 mL) and extracted with EtOAc (3 × 200 mL). The organic phases were washed with water, brine and dried over Na_2SO_4. After concentration *in vacuo*, the crude product was purified *via* FCC (Pent/acetone = 60:40). The solvent was removed and **6a** (1.76 g, 67 %) was obtained as a yellow solid.

TLC (Pent/acetone = 60:40): R_f = 0.24 (UV)

^{1}H-NMR (500 MHz, DMSO-d_6): δ [ppm] = 12.22 (s, 1H), 12.15 (s, 1H), 8.71 (d, J = 1.7 Hz, 1H), 8.13 (d, J = 2.9 Hz, 1H), 7.81 (dd, J = 8.6 Hz, 1.8 Hz, 1H), 7.55 (dd, J = 8.6 Hz, 0.7 Hz, 1H), 3.87 (s, 3H).

Methyl 3-(4-methylpiperidine-1-carbonyl)-1*H*-indole-5-carboxylate (6b): To a solution of **6a** (658 mg, 3.00 mmol, 1.0 eq.) in DMF (20 mL) was added DIPEA (2.09 mL, 12.0 mmol, 4.0 eq.) and HATU (2.01 g, 5.40 mmol, 1.8 eq.). The mixture was stirred for 15 min at RT. Subsequently, 4-methylpiperidine (0.72 mL, 6.00 mmol, 2.0 eq.) was added and the mixture was stirred overnight at RT. It was then quenched with an aq. 1 M HCl-solution and extracted with EtOAc (3 × 50 mL). The combined organic phases were washed with sat. aq. $NaHCO_3$-solution (50 mL), a 5 wt % aq. LiCl-solution (2 × 30 mL) and brine and dried over $MgSO_4$. The solvent was removed *in vacuo* and the crude product was purified *via* FCC (Pent/acetone = 60:40). The reaction yielded **6b** (98.0 mg, 11 %) as a pale-yellow oil.

TLC (Pent/acetone = 60:40): $R_f = 0.64$ (UV)

Mass spectrometry (EI-LRMS, positive): m/z: $[M]^+$ calcd. for $C_{17}H_{21}N_2O_3^+$: 301,15; found: 301.18.

3-(4-methylpiperidine-1-carbonyl)-1H-indole-5-carboxylic acid (6): To a solution of **6b** (98 mg, 0.33 mmol, 1.0 eq.) in 10 mL THF/H_2O (v/v = 1:1) was added LiOH·H_2O (42 mg, 0.99 mmol, 3.0 eq.). The mixture was stirred for 40 h at RT. The mixture was acidified to pH = 2 with an aq. 2 M HCl-solution. The aqueous phase was then extracted with EtOAc (3 × 20 mL), and the combined organic phases were washed with brine (20 mL), dried over $MgSO_4$ and concentrated *in vacuo* to give **6** (11 mg, 4 %) as a pale-yellow solid.

^{1}H-NMR (500 MHz, DMSO-d_6): δ [ppm] = 11.84 (s, 1H), 11.43 (s, 1H), 8.34 (d, J = 1.6 Hz, 1H), 7.76 (dd, J = 8.4 Hz, 2.0 Hz, 2H), 7.49 (d, J = 8.6 Hz, 1H), 3.01–2.89 (m, 4H), 1.25–1.22 (m, 3H), 1.09 (d, J = 12.6 Hz, 2H), 0.93 (d, J = 6.1 Hz, 3H).

^{13}C-NMR (101 MHz, DMSO-d_6): δ [ppm] = 168.2, 164.7, 145.7, 138.1, 129.9, 125.8, 123.0, 122.8, 111.7, 111.3, 38.3, 30.6, 29.2, 21.7.

Mass spectrometry (ESI-HRMS, positive): m/z: $[M+H]^+$ calcd. for $C_{16}H_{19}N_2O_3^+$: 287.13902; found: 287.13846.

Compound 7

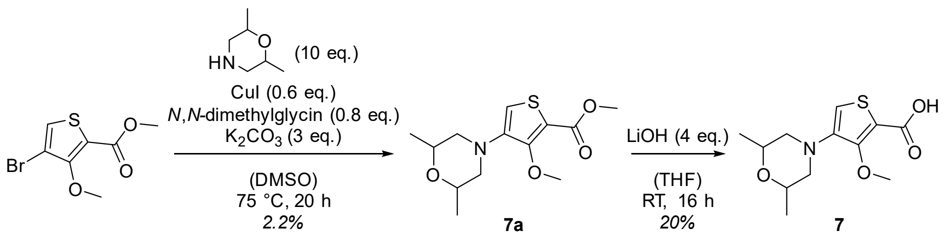

Figure S15: Synthesis overview of compound **7**.

Methyl 4-(2,6-dimethylmorpholino)-3-methoxythiophene-2-carboxylate (7a): A mixture of 4-bromo-3-methoxythiophene-2-carboxylic acid methyl ester (412 mg, 1.56 mmol, 1.0 eq.), 2,6-dimethylmorpholine (*cis*- and *trans*-mixture, 1.82 g, 15.3 mmol, 9.8 eq.), copper(I) iodide (178 mg, 0.94 mmol, 0.6 eq.), *N*,*N*-dimethylglycine hydrochloride (174 mg, 1.25 mmol, 0.8 eq.) and potassium carbonate (647 mg, 4.68 mmol, 3.0 eq.) in abs. dimethyl sulfoxide (3.1 mL) under argon atmosphere was stirred at 75 °C overnight. After completion, the mixture was cooled to RT, extracted with EtOAc (2×), washed with saturated aqueous NH_4Cl-solution (3×) and brine (2×). The organic layer was dried over $MgSO_4$ and concentrated *in vacuo*. The crude residue was purified by automated FCC (gradient elution: 0–30 % EtOAc in Hex) to afford **7a** (10.0 mg, 2.2 %).

TLC (Pent/EtOAc = 9:1): $R_f = 0.33$ (UV)

Mass spectrometry (EI-LRMS, positive): m/z: $[M]^+$ calcd. for $C_{13}H_{19}NO_4S^+$: 285.10; found: 285.1. (ESI-HRMS, positive): m/z: $[M+H]^+$ calcd. for $C_{13}H_{20}NO_4S^+$: 286.11076; found: 286.11075.

4-(2,6-Dimethylmorpholino)-3-methoxythiophene-2-carboxylic acid (7): To a solution of **7a** (9.8 mg, 34.3 µmol, 1.0 eq.) in THF (0.2 mL) was added lithium hydroxide (3.1 mg, 128 µmol, 3.7 eq.) dissolved in 0.1 mL water. The reaction mixture was stirred at RT until HPLC–MS confirmed full conversion to the corresponding carboxylic acid. To the reaction mixture, 100 µL of 1 M HCl was added and the mixture was thoroughly mixed. The

phases were separated, and the aqueous layer was extracted with DCM (3×). The combined organic layers were washed with brine, dried over $MgSO_4$, and concentrated *in vacuo*. The crude product was purified by preparative HPLC (20–80 % ACN in water with 0.1 %(v/v) TFA), affording the TFA salt of **7** (2.6 mg, 20 %) as a beige solid after lyophilization.

^{1}H-NMR (500 MHz, CD_3CN): δ [ppm] = 6.72 (s, 1H), 3.96 (s, 3H), 3.81–3.74 (m, 2H), 3.33 (dt, J = 10.5 Hz, 1.9 Hz, 2H), 2.23 (dd, J = 11.7 Hz, 10.2 Hz, 3H), 1.14 (d, J = 6.3 Hz, 6H).

^{13}C-NMR (101 MHz, CD_3CN): δ [ppm] = 162.21, 155.33, 145.79, 118.26, 117.48, 111.60, 72.30, 61.11, 56.89, 19.19, 1.89, 1.68, 1.47, 1.27, 1.06, 0.85, 0.65.

Mass spectrometry (ESI-HRMS, positive): m/z: $[M+H]^+$ calcd. for $C_{12}H_{18}NO_4S^+$: 272.09511; found: 272.09504. (ESI-HRMS, negative): m/z: $[M-H]^-$ calcd. for $C_{12}H_{16}NO_4S^-$: 270.08055; found: 270.08003.

Compound 8

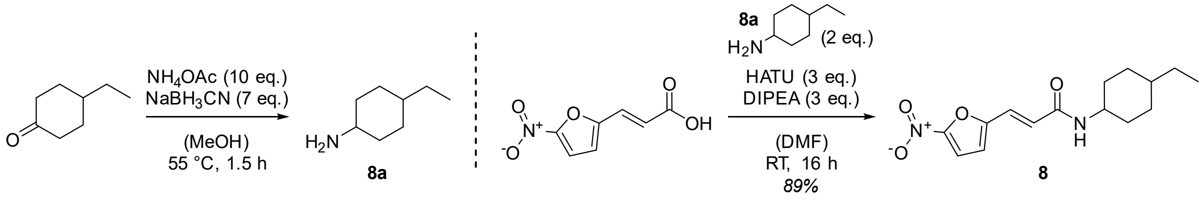

Figure S16: Synthesis overview of compound **8**.

4-Ethylcyclohexan-1-amine (8a): A mixture of 4-ethylcyclohexanone (808 mg, 6.40 mmol, 1.0 eq.), ammonium acetate (4933 mg, 64.0 mmol, 10.0 eq.) and sodium cyanoborohydride (2.82 g, 44.8 mmol, 7.0 eq.) in methanol (100 mL) was heated to 55 °C for 1.5 h. After cooling to RT, the mixture was acidified to pH 2 using 6 M HCl and subsequently basified using 2 M NaOH. The mixture was extracted with DCM (2×), and the combined organic phases were dried and concentrated *in vacuo* to yield a *cis-* and *trans-*mixture of **8a** as a colorless slurry. The crude product was directly used in the next reaction.

Mass spectrometry (ESI-LRMS, positive): m/z: $[M+ACN+H]^+$ calcd. for $C_{10}H_{21}N_2^+$: 169.17; found: 169.12.

Mass spectrometry (EI-LRMS, positive): m/z: $[M]^+$ calcd. for $C_8H_{17}N^+$: 127.14; found: 127.2.

***(E)*-*N*-(4-Ethylcyclohexyl)-3-(5-nitrofuran-2-yl)acrylamide (8):** To a solution of 3-(5-nitro-2-furyl)acrylic acid (189 mg, 1.00 mmol, 1.0 eq.), **8a** (318 mg, 2.00 mmol, 2.0 eq., 80 % purity assumed), and HATU (1141 mg, 3.00 mmol, 3.0 eq.) in DMF (10 mL) was added DIPEA (388 mg, 3.00 mmol, 3.0 eq., 0.523 mL) dropwise. The reaction mixture was stirred at RT for 16 h.

The mixture was diluted with EtOAc and a saturated aqueous $NaHCO_3$ solution was added and thoroughly mixed. The layers were separated, and the aqueous phase was extracted with EtOAc (3×). The combined organic layers were washed with 5 % aqueous LiCl (5×) and brine, and dried over $MgSO_4$. The crude product was purified by automated FCC (gradient elution: 10–50 % EtOAc in Hex), yielding a *cis*- and *trans*-mixture of **8** (260 mg, 89 %) as beige crystals.

TLC (Pent/EtOAc = 50:50): $R_f = 0.60$ ($KMnO_4$)

Mass spectrometry (ESI-HRMS, positive): m/z: $[M+H]^+$ calcd. for $C_8H_{18}N^+$: 128.14338; found: 128.14358.

^{1}H-NMR (400 MHz, $CDCl_3$): δ [ppm] = 7.37 (dd, J = 15.3 Hz, 2.9 Hz, 1H), 7.32 (dd, J = 3.8 Hz, 2.9 Hz, 1H), 6.67 (t, J = 3.1 Hz, 1H), 6.66 (dd, J = 21.7 Hz, 15.8 Hz, 1H), 5.99 (d, J = 7.6 Hz, 0.35H), 5.75 (d, J = 8.3 Hz, 0.65H), 4.20–4.12 (m, 0.35H), 3.91–3.74 (m, 0.65H), 2.08–1.98 (m, 1.3H), 1.84–1.76 (m, 1.3H), 1.76–1.57 (m, 2.1H), 1.32–1.26 (m, 1.05H), 1.25–1.21 (m, 1.3H), 1.21–1.08 (m, 2.65H), 1.08–0.97 (m, 1.3H), 0.92–0.83 (m, 3H). ***Note:*** *The product consists of a cis- and trans-mixture in a ratio of 65:35, the tabulated NMR data reflects this through the reported integral values. We did not determine which isomer is more abundant.*

^{13}C-NMR (101 MHz, $CDCl_3$): δ [ppm] = 163.39, 163.36, 153.39, 153.38, 152.05, 125.89, 125.83, 125.55, 125.51, 114.82, 114.77, 113.40, 113.36, 77.48, 77.16, 76.84, 49.49, 46.29, 38.69, 37.64, 33.19, 31.45, 29.56, 29.51, 28.42, 27.70, 11.70, 11.68. ***Note:*** *The two isomers cause doubling of signals for most ^{13}C nuclei.*

Mass spectrometry (ESI-HRMS, positive): m/z: $[M+H]^+$ calcd. for $C_{15}H_{21}N_2O_4^+$: 293.14958; found: 293.14937.

Compound 9

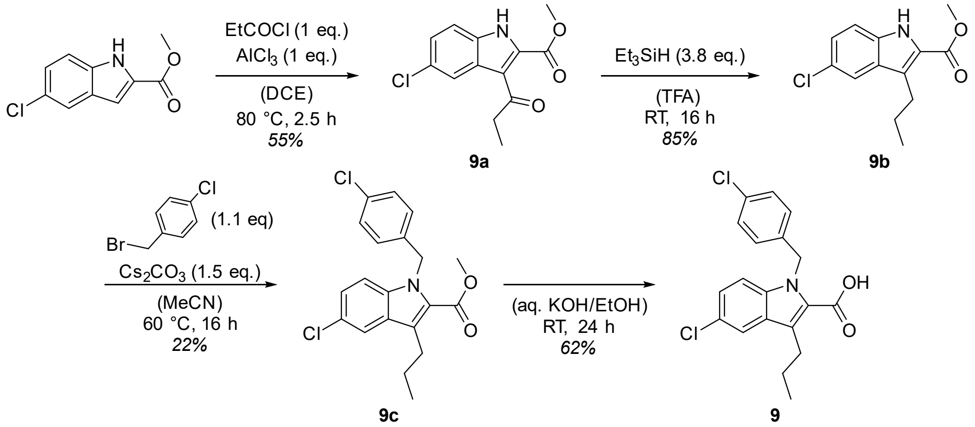

Figure S17: Synthesis overview of compound **9**.

Methyl-5-chloro-3-propionyl-1*H*-indole-2-carboxylate (9a): In a flask under argon atmosphere, methyl-5-chloro-1*H*-indole-2-carboxylate (1.07 g, 5.00 mmol, 1.0 eq.) was added to a mixture of propionyl chloride (0.45 mL, 5.00 mmol, 1.0 eq.) and $AlCl_3$ (677 mg, 5.00 mmol, 1.0 eq.) in abs. 1,2-dichloroethane (15 mL). The reaction mixture was stirred for 2.5 h at 80 °C and then allowed to reach RT. Ice-cold water (10 mL) and 3 M HCl-solution was added. The reaction mixture was extracted with DCM (3 × 20 mL). The combined organic phases were washed with brine, dried over Na_2SO_4 and the solvent removed *in vacuo*. The crude product was purified *via* FCC (Pent/EtOAc = 50:50) and after removing the solvent, **9a** (734 mg, 55 %) was obtained as a pale-yellow solid.

TLC (Pent/EtOAc = 50:50): $R_f = 0.76$ (UV)

^{1}H-NMR (500 MHz, $CDCl_3$): δ [ppm] = 7.99 (dt, J = 1.8 Hz, 0.7 Hz, 1H), 7.38–7.32 (m, 2H), 3.99 (s, 3H), 3.08 (q, J = 7.3 Hz, 2H), 1.24 (t, J = 7.3 Hz, 3H).

Methyl 5-chloro-3-propyl-1*H*-indole-2-carboxylate (9b) **9a** (106 mg, 0.40 mmol, 1.0 eq.), triethylsilane (0.26 mL, 1.60 mmol, 4.0 eq.) and TFA (1 mL) were added to a flask under an argon atmosphere and stirred overnight at RT. The mixture was then quenched with sat. aq. Na_2CO_3-solution and extracted with EtOAc (3 × 10 mL). The combined organic phases were washed with a sat. aq. $NaHCO_3$-solution, brine and dried over Na_2SO_4. After removing the solvent, the crude product was triturated in pentane. The supernatant was discarded and **9b** (86.7 mg, 85 %) was obtained as a colorless solid.

^{1}H-NMR (500 MHz, $CDCl_3$): δ [ppm] = 7.64 (dd, J = 1.8 Hz, 0.9 Hz, 1H), 7.32–7.23 (m, 2H), 3.95 (s, 3H), 3.06–3.00 (m, 2H), 1.74–1.63 (m, 2H), 0.97 (t, J = 7.3 Hz, 3H).

Methyl 5-chloro-1-(4-chlorobenzyl)-3-propyl-1*H*-indole-2-carboxylate (9c): **9b** (85.6 mg, 0.34 mmol, 1.0 eq.) was dissolved in abs. ACN (3 mL) and Cs_2CO_3 (166 mg, 0.51 mmol, 1.5 eq.) was added. The reaction mixture was stirred for 15 min at RT. Subsequently, 1-(bromomethyl)-4-chlorobenzene (78.4 mg, 0.37 mmol, 1.1 eq.) was added and the reaction mixture was stirred overnight at 60 °C. It was concentrated *in vacuo* and the residue dissolved in DCM (15 mL), which was then washed with water (2 × 10 mL). The organic phase was dried over Na_2SO_4 and concentrated *in vacuo*. The reaction yielded **9c** (28.5 mg, 22 %) as a pale-yellow oil, which crystallized after a few hours.

^{1}H-NMR (500 MHz, $CDCl_3$): δ [ppm] = 7.67 (d, J = 2.0 Hz, 1H), 7.31 (m, 1H), 7.25–7.18 (m, 3H), 6.93–6.88 (m, 2H), 5.69 (s, 2H), 3.87 (s, 3H), 3.05–2.99 (m, 2H), 1.73–1.62 (m, 2H), 0.98 (t, J = 7.4 Hz, 3H).

5-Chloro-1-(4-chlorobenzyl)-3-propyl-1*H*-indole-2-carboxylic acid (9): To a solution of **9c** (26.3 mg, 0.07 mmol, 1.0 eq.) in abs. EtOH (3 mL), was added 1 M aq. KOH-

solution (1 mL). The reaction mixture was stirred for 24 h at RT. After complete reaction, the EtOH was removed *via* rotary evaporation and the remaining aq. phase was acidified to pH = 3 with 1 M aq. HCl-solution. The precipitate was filtered. The crude product was purified *via* FCC (Pent/EtOAc = 90:10 → 50:50). After removing the solvent *in vacuo*, **9** was obtained as a colorless solid (15.6 mg, 62 %).

TLC (Pent/EtOAc = 90:10): R_f = 0.15 (UV)

^{1}H-NMR (500 MHz, $CDCl_3$): δ [ppm] = 7.70 (dd, J = 2.0 Hz, 0.6 Hz, 1H), 7.29–7.27 (m, 1H), 7.25–7.18 (m, 3H), 6.95–6.88 (m, 2H), 5.72 (s, 2H), 3.11–3.05 (m, 2H), 1.76–1.65 (m, 2H), 0.99 (t, J = 7.4 Hz, 3H).

^{13}C-NMR (101 MHz, DMSO-d_6): δ [ppm] = 137.8, 136.3, 131.6, 128.5, 128.1, 127.6, 125.2, 124.8, 124.3, 119.8, 112.3, 46.9, 26.4, 24.0, 13.9.

Mass spectrometry (ESI-HRMS, negative): m/z: $[M-H]^-$ calcd. for $C_{19}H_{16}Cl_2NO_2^-$: 360.05636; found: 360.05605.

Compound 10

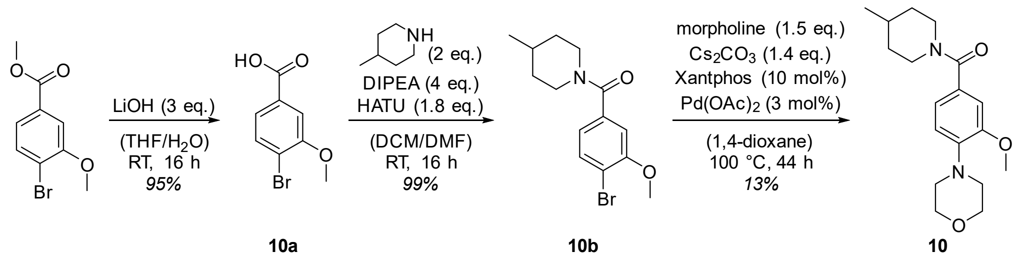

Figure S18: Synthesis overview of compound **10**.

4-Bromo-3-methoxybenzoic acid (10a): Methyl-4-bromo-3-methoxybenzoate (3.06 g, 12.5 mmol, 1.0 eq.) was dissolved in THF (40 mL) and water (40 mL). To this, LiOH (900 mg, 37.5 mmol, 3.0 eq.) was added and the solution was stirred overnight at RT. After complete reaction, the mixture was acidified to pH = 2 with a 2 M aq. HCl-solution (100 mL). The suspension was then extracted with EtOAc (2 × 100 mL). After removing the solvent *in*

vacuo, the reaction gave **10a** (2.74 g, 95 %) as colorless needle-like crystals.

Mass spectrometry (ESI-LRMS, negative): m/z: $[M-H]^-$ calcd. for $C_8H_6BrO_3^-$: 228.95; found: 229.05.

4-Bromo-3-methoxyphenyl)(4-methylpiperidin-1-yl)methanone (10b): 10a (231 mg, 1.00 mmol, 1.0 eq.) was dissolved in DCM (25 mL) and DMF (15 mL). To the solution was added DIPEA (0.70 mL, 4.00 mmol, 4.0 eq.) and HATU (684 mg, 1.80 mmol, 1.8 eq.), which was then stirred for 15 min at RT. Subsequently, 4-methylpiperidine (0.24 mL, 2.00 mmol, 2.0 eq.) was added and the solution stirred overnight at RT. After complete reaction, the solution was diluted with EtOAc and washed with a 5 wt % aq. LiCl-solution. After removing the solvent *in vacuo*, the crude product was purified *via* FCC (Pent/EtOAc = 50:50), which gave **10b** (309 mg, 99 %) as a pale orange oil.

TLC (Pent/EtOAc = 50:50): R_f = 0.67 (UV)

^{1}H-NMR (500 MHz, $CDCl_3$): δ [ppm] = 7.54 (d, J = 8.0 Hz, 1H), 6.95 (d, J = 1.8 Hz, 1H), 6.82 (dd, J = 7.9 Hz, 1.8 Hz, 1H), 4.65–4.62 (m, 1H), 3.91 (s, 4H), 3.71–3.68 (m, 1H), 3.00–2.96 (m, 1H), 2.82–2.74 (m, 1H), 1.76 (s, 1H), 1.65–1.56 (m, 1H), 1.22–1.21 (m, 1H), 1.11–1.08 (m, 1H), 0.97 (d, J = 6.4 Hz, 3H).

(3-Methoxy-4-morpholinophenyl)(4-methylpiperidin-1-yl)methanone (10): 10b (156 mg, 0.50 mmol, 1.0 eq.), $Pd(OAc)_2$ (3.37 mg, 0.02 mmol, 0.03 eq.), Xantphos (26.9 mg, 0.05 mmol, 0.09 eq.) and Cs_2CO_3 (228 mg, 0.70 mmol, 1.4 eq.) were added to a flask under argon atmosphere. The solids were dissolved in 1,4-dioxane (5 mL) and morpholine (0.07 mL, 0.75 mmol, 1.5 eq.) was added. The reaction mixture was stirred at 100 °C for 44 h. The reaction was quenched with a sat. aq. NH_4Cl-solution (15 mL), extracted with EtOAc (3 × 15 mL), washed with brine and dried over Na_2SO_4. After removing the solvent *in vacuo*, the crude product was purified *via* FCC (Pent/EtOAc = 30:70). The reaction yielded **10** (21.4 mg, 13 %) as a beige solid.

TLC (Pent/EtOAc = 30:70): R_f = 0.37 (UV)

^{1}**H-NMR** (500 MHz, $CDCl_3$): δ [ppm] = 6.99–6.92 (m, 2H), 6.87 (d, J = 8.5 Hz, 1H), 3.88 (d, J = 4.5 Hz, 7H), 3.12–3.06 (m, 4H), 2.97–2.78 (m, 4H), 1.51–1.44 (m, 2H), 1.42–1.35 (m, 1H), 1.21–1.07 (m, 2H), 0.97 (d, J = 6.4 Hz, 3H).

^{13}**C-NMR** (101 MHz, $CDCl_3$): δ [ppm] = 170.4, 152.1, 142.4, 131.9, 120.0, 117.4, 110.8, 67.2, 60.5, 55.7, 51.1, 31.4, 21.9, 14.3.

Mass spectrometry (ESI-HRMS, positive): m/z: $[M+H]^+$ calcd. for $C_{18}H_{27}N_2O_3^+$: 319.20162; found: 319.20133.

Compound 11

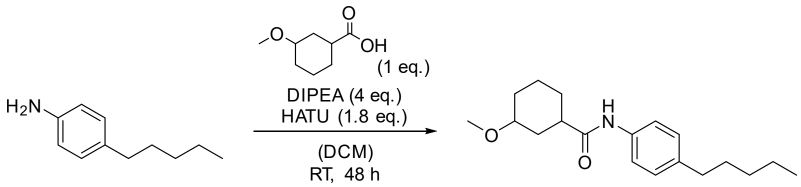

Figure S19: Synthesis overview of compound **11**.

3-Methoxy-*N*-(4-pentylphenyl)cyclohexane-1-carboxamide (11): 3-Methoxy-cyclohexanecarboxylic acid (163 mg, 1.00 mmol, 1.0 eq.) was dissolved in abs. DCM (15 mL) and DIPEA (0.70 mL, 4.00 mmol, 4.0 eq.) and HATU (684 mg, 1.80 mmol, 1.8 eq.) were added. After 15 min of stirring, 4-pentylaniline (0.36 mL, 2.00 mmol, 2.0 eq.) was added and the mixture stirred for 48 h at RT. The reaction was then quenched with 2 M aq. HCl-solution and extracted with DCM. The combined organic phases were washed with a sat. aq. $NaHCO_3$-solution and brine. After removing the solvent *in vacuo*, the crude product was purified *via* FCC (Pent/EtOAc = 80:20). The reaction gave **11** (123 mg, 41 %) as colorless crystals.

TLC (Pent/EtOAc = 80:20): R_f = 0.38 (UV)

^{1}**H-NMR** (500 MHz, $CDCl_3$): δ [ppm] = 7.43–7.39 (m, 2H), 7.15–7.09 (m, 2H), 3.38 (s, 3H), 3.22 (tt, J = 10.2 Hz, 3.8 Hz, 1H), 2.55 (t, J = 7.7 Hz, 2H), 2.29 (m, 2H), 1.90 (m, J =

12.5 Hz, 6.2 Hz, 3.6 Hz, 2H), 1.60–1.54 (m, 2H), 1.53–1.45 (m, 2H), 1.41–1.28 (m, 6H), 0.93 (t, J = 7.4 Hz, 1H), 0.88 (t, J = 6.9 Hz, 4H).

^{13}C-NMR (101 MHz, $CDCl_3$): δ [ppm] = 173.0, 139.2, 135.7, 129.0, 120.0, 78.6, 55.9, 45.0, 35.5, 34.7, 31.5, 31.4, 31.3, 29.2, 23.3, 22.7, 14.2.

Mass spectrometry (ESI-HRMS, positive): m/z: $[M+H]^+$ calcd. for $C_{19}H_{30}NO_2^+$: 304.22711; found: 304.22623.

Compound D8

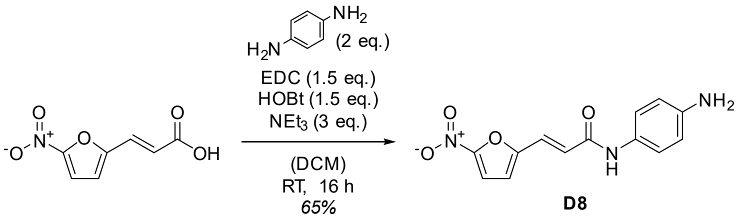

Figure S20: Synthesis overview of compound **D8**.

(*E*)-*N*-(4-Aminophenyl)-3-(5-nitrofuran-2-yl)acrylamide (D8): 3-(5-Nitro-2-furyl) acrylic acid (378 mg, 2.00 mmol, 1.0 eq.), *p*-phenylenediamine (433 mg, 4.00 mmol, 2.0 eq.), 1-hydroxybenzotriazole hydrate (HOBt, 405 mg, 3.00 mmol, 1.5 eq.) and 1-ethyl-3-(3-dimethylaminopropyl)carbodiimide hydrochloride (EDC·HCl, 575 mg, 3.00 mmol, 1.5 eq.) were dissolved in abs. DCM (20 mL). Triethylamine (842 µL, 6.00 mmol, 3.0 eq.) was added and the reaction mixture was stirred at RT overnight. The reaction was diluted with additional DCM (20 mL) and washed with saturated aqueous $NaHCO_3$ (2 × 30 mL) and brine. The organic layer was dried over anhydrous $MgSO_4$ and concentrated *in vacuo* to give **D8** (357 mg, 65 %) as a dark red solid. A small portion (20 mg) of the crude material was purified by preparative HPLC (30–98 % ACN in water with 0.1 % (v/v) TFA) to give the TFA salt as an orange solid.

^{1}H-NMR (400 MHz, DMSO-d_6): δ [ppm] = 10.44 (s, 1H), 8.50 (s, 3H), 7.76 (d, J = 3.8 Hz, 1H), 7.70–7.61 (m, 2H), 7.46 (d, J = 15.6 Hz, 1H), 7.18 (d, J = 3.9 Hz, 1H), 7.10–7.02

(m, 2H), 6.97 (d, J = 15.6 Hz, 1H).

^{13}C-NMR (101 MHz, DMSO-d$_6$): δ [ppm] = 161.85, 153.12, 151.62, 134.92, 132.55, 125.81, 125.33, 120.31, 120.15, 116.52, 114.69, 40.15, 39.94, 39.73, 39.52, 39.31, 39.10, 38.89.

Mass spectrometry (ESI-HRMS, positive): m/z: $[M+H]^+$ calcd. for $C_{13}H_{12}N_3O_4^+$: 274.08223; found: 274.08211.

Compound D25

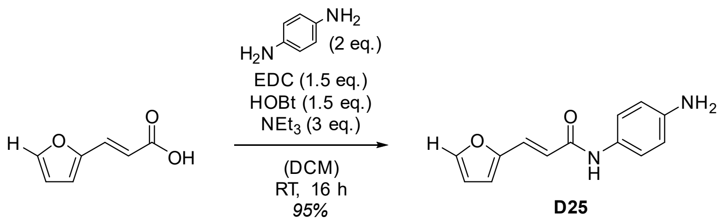

Figure S21: Synthesis overview of compound **D25**.

***(E)*-*N*-(4-Aminophenyl)-3-(furan-2-yl)acrylamide (D25):** To a solution of (*E*)-3-(furan-2-yl)acrylic acid (69.1 mg, 0.50 mmol, 1.0 eq.) in abs. DCM (5.0 mL) were added *p*-phenylenediamine (108 mg, 1.00 mmol, 2.0 eq.), HOBT (101.3 mg, 0.75 mmol, 1.5 eq.), and EDC·HCl (143.8 mg, 0.75 mmol, 1.5 eq.). Triethylamine (211 µL, 1.50 mmol, 3.0 eq.) was added, and the reaction mixture was stirred at RT overnight. DCM (5.0 mL) was added and the reaction mixture was washed with saturated aqueous $NaHCO_3$ (2 × 5.0 mL) and brine, then dried over anhydrous $MgSO_4$. The mixture was filtered and concentrated *in vacuo* to give the crude product (108 mg, 95 %). A small portion was purified by preparative HPLC (30–98 % ACN in water with 0.1 % TFA) to yield the TFA salt of **D25** as a yellow solid.

^{1}H-NMR (400 MHz, DMSO-d$_6$): δ [ppm] = 10.28 (s, 1H), 8.88 (s, 3H), 7.81 (d, J = 1.8 Hz, 1H), 7.77–7.60 (m, 2H), 7.38 (d, J = 15.4 Hz, 1H), 7.25–7.06 (m, 2H), 6.85 (d, J = 3.4 Hz, 1H), 6.63 (d, J = 15.4 Hz, 1H), 6.62 (dd, J = 3.3 Hz, 1.8 Hz, 1H).

^{13}C-NMR (101 MHz, DMSO-d$_6$): δ [ppm] = 163.34, 150.87, 145.13, 136.83, 130.35, 127.30, 121.53, 120.20, 119.22, 114.54, 112.52, 40.15, 39.94, 39.73, 39.52, 39.31, 39.10, 38.89.

Mass spectrometry (ESI-HRMS, positive): m/z: $[M+H]^+$ calcd. for $C_{13}H_{13}N_2O_2^+$: 229.09715; found: 229.09705.

Compound D26

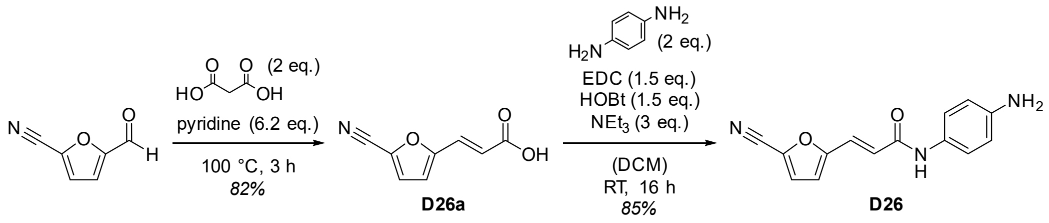

Figure S22: Synthesis overview of compound **D26**.

(*E*)-3-(5-Cyanofuran-2-yl)acrylic acid (D26a): A mixture of 5-formylfuran-2-carbonitrile (127 mg, 1.00 mmol, 1.0 eq.) and malonic acid (208 mg, 2.00 mmol, 2.0 eq.) in pyridine (500 µL) was stirred at 100 °C for 3 h. After the cessation of gas evolution, the mixture was cooled to RT. The residue was diluted with 10 mL of 2 M HCl and extracted with EtOAc (3 × 10 mL). The combined organic layers were washed with 2 M HCl, water, and brine, dried over $MgSO_4$, filtered, and concentrated *in vacuo* to yield compound **D26a** (133 mg, 82 %) as a colourless solid.

^{1}H-NMR (500 MHz, DMSO-d$_6$): δ [ppm] = 12.64 (s, 1H), 7.69 (d, J = 3.7 Hz, 1H), 7.43 (d, J = 16.0 Hz, 1H), 7.16 (d, J = 3.8 Hz, 1H), 6.43 (d, J = 16.0 Hz, 1H).

^{13}C-NMR (101 MHz, DMSO-d$_6$): δ [ppm] = 166.54, 154.84, 129.02, 125.66, 125.29, 121.62, 115.09, 111.53, 40.15, 39.94, 39.73, 39.52, 39.31, 39.10, 38.89.

(*E*)-*N*-(4-Aminophenyl)-3-(5-cyanofuran-2-yl)acrylamide (D26): To a solution of **D26a** (122 mg, 0.75 mmol, 1.0 eq.) in abs. DCM (7.5 mL) was added *p*-phenylenediamine (162 mg, 1.50 mmol, 2.0 eq.), HOBt (152 mg, 1.13 mmol, 1.5 eq.) and EDC·HCl (216 mg, 1.13 mmol, 1.5 eq.). Triethylamine (230 mg, 2.25 mmol, 3.0 eq., 316 µL) was added, and

the reaction mixture was stirred at RT overnight. DCM (20 mL) was added and the mixture was washed with saturated aqueous $NaHCO_3$ (30 mL × 2) and brine, then dried over anhydrous $MgSO_4$, filtered, and concentrated *in vacuo*. The crude product was purified by preparative HPLC (30–98 % ACN with 0.1 % TFA), yielding the TFA salt of **D26** as a yellow solid.

^{1}H-NMR (300 MHz, DMSO-d_6): δ [ppm] = 10.45 (s, 1H), 8.91 (s, 2H), 7.70 (d, J = 3.6 Hz, 2H), 7.69 (d, J = 9.3 Hz, 3H), 7.44 (d, J = 15.7 Hz, 1H), 7.20–7.11 (m, 2H), 7.09 (d, J = 3.8 Hz, 1H), 6.86 (d, J = 15.7 Hz, 1H).

^{13}C-NMR (101 MHz, DMSO-d_6): δ [ppm] = 162.24, 155.42, 135.79, 131.92, 125.46, 125.33, 125.21, 124.62, 120.94, 120.33, 114.62, 111.61, 40.15, 39.94, 39.73, 39.52, 39.31, 39.10, 38.89.

Mass spectrometry (ESI-HRMS, positive): m/z: $[M+H]^+$ calcd. for $C_{14}H_{12}N_3O_2^+$: 254.09240; found: 254.09222.

Compound D27

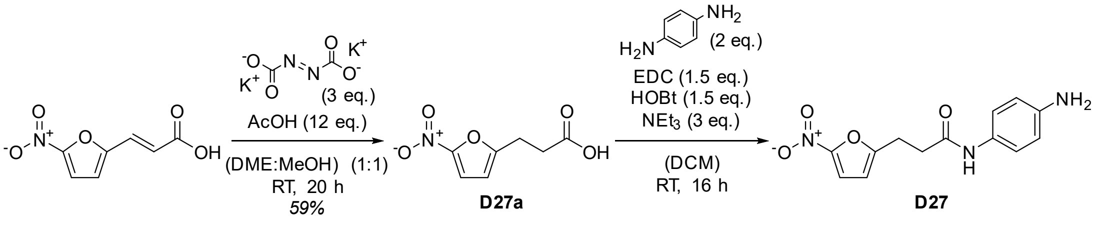

Figure S23: Synthesis overview of compound **D27**.

3-(5-Nitrofuran-2-yl)propanoic acid (D27a): To a solution of 3-(5-nitro-2-furyl)acrylic acid (189 mg, 1.00 mmol, 1.0 eq.) in a mixture of DME (5.0 mL) and MeOH (4.2 mL), freshly prepared potassium azodicarboxylate (589 mg, 3.00 mmol, 3.0 eq.)[S11] was added over 15 min. The glacial AcOH was taken up in DME:MeOH (1:1, 1.0 mL) and slowly added dropwise over 10 h at RT. The reaction mixture was stirred for an additional 10 h before the solvents were removed *in vacuo*. The residue was partitioned between EtOAc and 2 M HCl. After

confirming pH < 2, the aqueous layer was re-extracted with EtOAc. The combined organic phases of the acidic extraction were washed with 2 M HCl and brine, dried over $MgSO_4$, and concentrated *in vacuo* to afford **D27a** (110 mg, 59 %) as a yellow solid.

^{1}H-NMR (400 MHz, $CDCl_3$): δ [ppm] = 7.24 (d, J = 3.6 Hz, 1H), 6.34 (d, J = 3.6 Hz, 1H), 3.08 (t, J = 7.3 Hz, 2H), 2.81 (t, J = 7.3 Hz, 2H).

^{13}C-NMR (101 MHz, $CDCl_3$): δ [ppm] = 177.24, 157.98, 151.74, 112.86, 110.08, 77.48, 77.16, 76.84, 31.53, 23.55, 20.81, 1.15.

Mass spectrometry (ESI-HRMS, negative): m/z: $[M\text{-}H]^-$ calcd. for $C_7H_6NO_5^-$: 184.02515; found: 184.02520.

***N*-(4-Aminophenyl)-3-(5-nitrofuran-2-yl)propanamide (D27):** To a solution of **D27a** (37.0 mg, 0.20 mmol, 1.0 eq.) in abs. DCM (2.0 mL) were added *p*-phenylenediamine (43.3 mg, 0.40 mmol, 2.0 eq.), HOBT (40.5 mg, 0.30 mmol, 1.5 eq.), and EDC·HCl (57.5 mg, 0.30 mmol, 1.5 eq.). Triethylamine (84.2 µL, 0.60 mmol, 3.0 eq.) was added, and the reaction mixture was stirred at RT overnight. DCM (20.0 mL) was added and the mixture was washed with saturated aqueous $NaHCO_3$ (2 × 30.0 mL) and brine. The organic layer was dried over anhydrous $MgSO_4$, filtered, and concentrated *in vacuo*. The crude product was purified by preparative HPLC (30–98 % ACN in water with 0.1 % TFA) to yield the TFA salt of **D27** as a beige solid.

^{1}H-NMR (400 MHz, DMSO-d_6): δ [ppm] = 10.04 (s, 1H), 7.63 (d, J = 3.7 Hz, 1H), 7.56–7.51 (m, 2H), 7.06 (d, J = 8.7 Hz, 2H), 6.62 (d, J = 3.7 Hz, 1H), 3.07 (t, J = 7.3 Hz, 2H), 2.74 (t, J = 7.4 Hz, 2H).

Mass spectrometry (ESI-HRMS, positive): m/z: $[M+H]^+$ calcd. for $C_{13}H_{14}N_3O_4^+$: 276.09788; found: 276.09778.

Automated Synthesis of SAR Derivatives

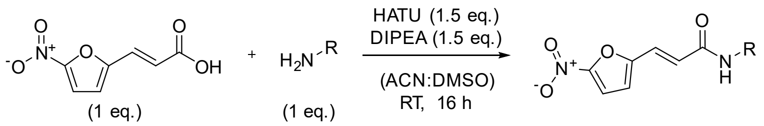

Figure S24: Synthesis overview of the automated synthesis panel.

A 0.5 M solution of 3-(5-nitro-2-furyl)acrylic acid was prepared in a 3:1 mixture of ACN and DMSO (Solution A). A 0.75 M solution of HATU was prepared in a 3:1 mixture of ACN and DMSO (Solution B). Of 48 amine precursors, 0.10 mmol were weighed into 1.5 mL microcentrifuge tubes and dissolved in 50 µL ACN to give 2 M solutions.

A Hamilton Microlab STARlet robot was programmed to transfer all of every amine stock from the microcentrifuge tubes into wells of a 96-well deep-well plate, followed by 200 µL of Solution A and 200 µL of Solution B. Lastly, the stepping function was used to add 26 µL of DIPEA to every well (see protocol attached below).

For amine precursors that came as salts, another 26 µL (0.15 mmol, 1.5 eq.) of DIPEA per equivalent of acid were added manually to the respective well, before the plate was sealed and shaken in a thermocycler with 200 rpm at RT overnight.

After completion, the reaction mixtures were transferred into 15 mL centrifuge tubes with 4 mL water and centrifuged at 10 000 ×g for 15 min. The supernatant was decanted into fresh 15 mL centrifuge tubes. The remaining residue was resuspended in 2.5 mL water by vortexing, centrifuged again, and the supernatant was combined with the first decant. The pelleted precipitates were dried in a desiccator with orange gel under vacuum overnight. 20 mM stocks in DMSO were prepared from all dried precipitates, assuming the molar mass of the pure amide coupling products.

2 µL of the DMSO stocks were diluted with 98 µL of a 1:1 ACN/water-mixture in a 96-well plate and analyzed by HPLC–MS.

	Method
1	Initialize (Single Step) on ML_STAR<br>Always initialize: Off<br>3 return value(s) .
2	Comment<br><Add Amines from first 32 Eppis into DWB>
3	Sequence: Set Current Position<br>current position of sequence 'ML_STAR.SMP_CAR_32_EPIL_A00_0001' = '1'
4	Sequence: Set End Position<br>end position of sequence 'ML_STAR.SMP_CAR_32_EPIL_A00_0001' = '32'
5	Sequence: Set Current Position<br>current position of sequence 'ML_STAR.Cos_96_DW_1mL_0001' = '1'
6	Sequence: Set End Position<br>end position of sequence 'ML_STAR.Cos_96_DW_1mL_0001' = '48'
7	Loop<br>'4' times<br>'loopCounter2' used as loop counter variable
8	1000µl Channel Aspirate on ML_STAR<br>Sequence: ML_STAR.SMP_CAR_32_EPIL_A00_0001, Volume [µl]: 200<br>0 return value(s) .
9	1000µl Channel Dispense on ML_STAR<br>Sequence: ML_STAR.Cos_96_DW_1mL_0001, Volume [µl]: Remaining volume inclusive blowout air<br>0 return value(s) .
10	End Loop
11	Sequence: Set Current Position<br>current position of sequence 'ML_STAR.Cos_96_DW_1mL_0001' = '33'
12	Comment<br><Add Amines from Eppis 33-48 into DWB>
13	Sequence: Set End Position<br>end position of sequence 'ML_STAR.Cos_96_DW_1mL_0001' = '48'
14	Sequence: Set Current Position<br>current position of sequence 'ML_STAR.SMP_CAR_32_EPIL_A00_0002' = '1'
15	Sequence: Set End Position<br>end position of sequence 'ML_STAR.SMP_CAR_32_EPIL_A00_0002' = '16'
16	Loop<br>'2' times<br>'loopCounter2' used as loop counter variable
17	1000µl Channel Aspirate on ML_STAR<br>Sequence: ML_STAR.SMP_CAR_32_EPIL_A00_0002, Volume [µl]: 200<br>0 return value(s) .
18	1000µl Channel Dispense on ML_STAR<br>Sequence: ML_STAR.Cos_96_DW_1mL_0001, Volume [µl]: Remaining volume inclusive blowout air<br>0 return value(s) .
19	End Loop
20	Comment<br><Add 100 µL Carboxylic acid from basin 1 into DWB>
21	Sequence: Set Current Position<br>current position of sequence 'ML_STAR.Cos_96_DW_1mL_0001' = '1'
22	Sequence: Set End Position<br>end position of sequence 'ML_STAR.Cos_96_DW_1mL_0001' = '48'
23	Loop<br>'6' times<br>'loopCounter3' used as loop counter variable
24	1000µl Channel Aspirate on ML_STAR<br>Sequence: ML_STAR.rgt_cont_50ml_a00_0001, Volume [µl]: 200<br>0 return value(s) .
25	1000µl Channel Dispense on ML_STAR<br>Sequence: ML_STAR.Cos_96_DW_1mL_0001, Volume [µl]: Remaining volume inclusive blowout air<br>0 return value(s) .
26	End Loop
27	1000µl Channel Tip Eject from Microlab® STAR Smart Steps<br>Instrument short name 'ML_STAR'.

	Method
28	Comment<br><Add 200 µL HATU from basin 2 into DWB>
29	Sequence: Set Current Position<br>current position of sequence 'ML_STAR.Cos_96_DW_1mL_0001' = '1'
30	Sequence: Set End Position<br>end position of sequence 'ML_STAR.Cos_96_DW_1mL_0001' = '48'
31	Loop<br>'6' times<br>'loopCounter3' used as loop counter variable
32	1000µl Channel Aspirate on ML_STAR<br>Sequence: ML_STAR.rgt_cont_50ml_a00_0002, Volume [µl]: 200<br>0 return value(s) .
33	1000µl Channel Dispense on ML_STAR<br>Sequence: ML_STAR.Cos_96_DW_1mL_0001, Volume [µl]: Remaining volume inclusive blowout air<br>0 return value(s) .
34	End Loop
35	1000µl Channel Tip Eject from Microlab® STAR Smart Steps<br>Instrument short name 'ML_STAR'.
36	Sequence: Set Current Position<br>current position of sequence 'ML_STAR.Cos_96_DW_1mL_0001' = '1'
37	Sequence: Set End Position<br>end position of sequence 'ML_STAR.Cos_96_DW_1mL_0001' = '48'
38	Sequence: Set Current Position<br>current position of sequence 'ML_STAR.SMP_CAR_32_EPIL_A00_0003' = '1'
39	Sequence: Set End Position<br>end position of sequence 'ML_STAR.SMP_CAR_32_EPIL_A00_0003' = '8'
40	1000µl Channel Pipette - Aliquot from Microlab® STAR Smart Steps<br>Instrument short name 'ML_STAR', Aliquot procedure, Volume per well [µl]: '26'.<br>Aspirate sequence: 'ML_STAR.SMP_CAR_32_EPIL_A00_0003', Dispense sequence:<br>'ML_STAR.Cos_96_DW_1mL_0001 (controlling sequence)'.
41	1000µl Channel Tip Eject from Microlab® STAR Smart Steps<br>Instrument short name 'ML_STAR'.
42	

Biological Evaluation

Chemicals

Table S6: List of chemical reagents and their suppliers.

**Chemical**	**Acronym**	**Supplier**
Agar-Agar	Agar	Merck Millipore
Ampicillin sodium salt		Carl Roth
Dimethyl sulfoxide	DMSO	Sigma Aldrich
Disodium hydrogen phosphate	Na_2HPO_4	Carl Roth
Ethanol	EtOH	Carl Roth
L-Glutathione, reduced form	L-glutathione (GSH)	Sigma Aldrich
Glycerol		Carl Roth
Hydrochloric acid	HCl	Fisher
Nitrofurantoin	NFA	Sigma Aldrich
Nitrofurazone	NFZ	Sigma Aldrich
Potassium chloride	KCl	Carl Roth
Potassium dihydrogen phosphate	KH_2PO_4	VWR
(+)-Sodium L-ascorbate	VitC	Alfa Aesar
Sodium bicarbonate	$NaHCO_3$	Fisher
Sodium chloride	NaCl	VWR
Sodium hydroxide	NaOH	Fisher
Tetracycline hydrochloride		Carl Roth
Ultrapure Water	ddH_2O	

Media and Buffers

Table S7: Composition of buffers and media used in the study.

Buffer/Medium	Composition
PBS	10 mM Na_2HPO_4<br>1.8 mM KH_2PO_4<br>140 mM NaCl<br>2.7 mM KCl<br>in ddH_2O, pH = 7.4
B-medium (Carl Roth)	0.5 % (w/v) yeast extract<br>1.0 % (w/v) peptone<br>0.5 % (w/v) NaCl<br>0.1 % (w/v) K_2HPO_4<br>in ddH_2O, pH = 7.5
LB-medium (Carl Roth)	0.5 % (w/v) yeast extract<br>1.0 % (w/v) peptone<br>0.5 % (w/v) NaCl<br>in ddH_2O, pH = 7.5
BHI-medium (Carl Roth)	0.75 % (w/v) brain infusion<br>1.0 % (w/v) heart infusion<br>1.0 % (w/v) peptone<br>0.5 % (w/v) NaCl<br>0.25 % (w/v) Na_2HPO_4<br>0.2 % (w/v) glucose<br>in ddH_2O, pH = 7.4
LB-agar (using LB-medium from Carl Roth)	0.5 % (w/v) yeast extract<br>1.0 % (w/v) peptone<br>0.5 % (w/v) NaCl<br>1.5 % (w/v) Agar<br>in ddH_2O, pH = 7.5

Bacterial Strains

Bacteria were stored at $-80\,°C$ as cryostocks in a 1:1 mixture of culture medium and glycerol. Aliquots were shock frozen in liquid N_2 and only thawed once.

Table S8: Bacterial strains and their corresponding culture media.

**Organism**	**Strain**	**Culture Medium**
Acinetobacter baumannii	DSM 30007	BHI
Enterobacter cloacae	DSM 30054	BHI
Enterococcus faecium	DSM 20477	LB
Escherichia coli	K12	LB
Escherichia coli	CFT073	LB
Klebsiella pneumoniae	DSM 30104	BHI
Pseudomonas aeruginosa	PAO1	LB
Staphylococcus aureus	USA300	B
Staphylococcus aureus	NE118	B + Erythromycin
Staphylococcus aureus	NE441	B + Erythromycin
Staphylococcus aureus	NE662	B + Erythromycin
Staphylococcus aureus	NE1550	B + Erythromycin

Assays

Minimal Inhibitory Concentration (MIC) Determination 5 mL of medium in a culture tube were inoculated at a 1:100 ratio with an overnight culture of the desired bacteria. The culture tube was then incubated until it reached at least 0.5 McFarland units.

2 µL of a DMSO stock at 100 times the highest test concentration were added to wells B–D and E–G in the first column of a 96-well plate (two different conditions in technical triplicates per plate). A sterile-filtered aqueous 500 mM $NaHCO_3$ solution was diluted 1:20 in culture medium. 100 µL of this medium were added to each well in rows A and H to serve as a sterile control. Additionally, 50 µL of medium were dispensed into columns 2–12 of rows

B–G. 98 µL of medium were added to the stocks in wells B–G of column 1, resulting in a final volume of 100 µL with twice the highest test concentration of the compound.

The diluted compounds in the first column were thoroughly mixed by pipetting up and down. From the first column, 50 µL were transferred to the second column and mixed, followed by sequential transfers of 50 µL from one column to the next until column 10 was reached. 50 µL from column 10 were transferred to column 12, which served as the sterile control for the dilution process. Column 11 served as the untreated growth control.

If necessary, the bacterial suspension was diluted to 0.5 McFarland units with fresh medium. The suspension was then diluted 1:100 in fresh medium and 50 µL of this suspension were then added to all wells except the sterile controls.

The plate was incubated for 16 h at 37 °C with shaking at 200 rpm. Optical density (OD_{600}) values were measured using a plate reader (Tecan Infinite® 200 Pro M Nano). The lowest concentration where no bacterial growth was observed ($OD_{600} \leq 0.1$) represents the MIC.

High-Throughput MIC Determination at Fixed Concentrations 60 mL of medium in a 250 mL culture flask were inoculated at a 1:100 ratio with an overnight culture. The culture was incubated until it reached at least 0.5 McFarland units.

One 96-well plate was used to test one fixed concentration of all compounds. Experiments were performed in biological triplicates (inoculated with separate day cultures), with one technical replicate of each compound per plate. Three compound concentrations (50, 100 and 200 µM) were tested in three bacterial strains (*S. aureus* USA300, *E. coli* K12, *P. aeruginosa* PAO1), resulting in 27 plates.

20 mM stocks of the compounds in DMSO were serially diluted to 10 and 5 mM with DMSO in a 96-well PCR plate. 1 µL of the 100x DMSO stocks was added to wells B3–G10 of a 96-well plate. 1 µL of DMSO was added to wells B–G of columns 2 and 11 as a vehicle control. 100 µL of medium were dispensed into each well of rows A and H, as well as columns

1 and 12, to serve as sterile controls and evaporation protection.

The bacterial suspension was diluted to 0.25 McFarland units with fresh medium and supplemented with 25 mM $NaHCO_3$. 99 µL of the bacterial suspension were added to all inner wells (B2–G11). Notably, no additional dilution step was performed before addition of the bacterial suspension to the plate, resulting in a bacterial load 100-fold higher than usual. This was done to increase the robustness of the screening and decrease the rate of false positives at the high compound concentrations and possibly low purity tested.[S12]

The plate was incubated for 16 h at 37 °C with shaking at 200 rpm. OD_{600} values were measured using a plate reader (Tecan Infinite® 200 Pro M Nano).

MICs in Presence of a Nitroreductase-Inhibitor The procedure for regular MIC determination described above was used. The 100-fold diluted bacterial suspension was supplemented with 100, 200 or 400 µM dicoumarol (DC) using a 10 mM stock of DC in DMSO before addition of this suspension to the plate.

To be able to better distinguish small differences in MIC, the MIC was not simply read out as the smallest concentration at which no growth was observed, but the MIC_{90} was determined. For this, the measured OD_{600} values after 16 h were plotted against the different concentrations of the dilution series and a nonlinear regression ([Agonist] vs. response – find ECanything) was fitted using GraphPad Prism v10.0.1. From this fit, the EC_{10} was calculated, which represents the MIC_{90}.

MICs in Transposon Mutants of *S. aureus* The procedure for regular MIC determination described above was used. While the overnight cultures and day cultures of the mutant strains from the Nebraska Transposon Mutant Library (NTML) were grown in medium supplemented with 10 µg/mL of erythromycin to select for the mutants carrying the correct genetic insert, the medium used for the plates was not supplemented to exclude synergistic effects between erythromycin and the compounds tested.

To be able to better distinguish small differences in MIC, the MIC was not simply read

out as the smallest concentration at which no growth was observed, but the MIC_{90} was determined. For this, the measured OD_{600} values after 16 h were plotted against the different concentrations of the dilution series and a nonlinear regression ([Agonist] vs. response – find ECanything) was fitted using GraphPad Prism v10.0.1. From this fit, the EC_{10} was calculated, which represents the MIC_{90}.

Time-Kill-Assay in Presence of Different ROS-Scavengers 25 mL of LB medium containing 12.5 mM $NaHCO_3$ were inoculated 1:200 with an overnight culture of *E. coli* in a 250 mL baffled flask. The culture was grown to an OD_{600} of $0.25 - 0.30$.

- A 1 M stock solution of the reduced form of L-L-glutathione (GSH) was prepared in 1 M NaOH (1 equivalent of base to deprotonate half of the carboxylic acids in GSH) and sterile-filtered.
- A 1 M stock solution of (+)-sodium L-ascorbate (vitamin C (VitC)) was prepared in ddH_2O and sterile-filtered.

495 µL of bacterial culture were added to the first column and 470 µL to the other wells of a sterile 24-well plate according to the plate layout (Tab. S9). 25 µL of the respective 20x antioxidant stocks were added.

Table S9: Plate layout.

D8	+ GSH	+ VitC
NFZ	+ GSH	+ VitC
DMSO	+ GSH	+ VitC

The plate was incubated for 10 min at 37 °C with shaking at 200 rpm.

5 µL of a 100x antibiotic stock or 5 µL DMSO were added to each well. The plate was mixed thoroughly, the samples for the starting point were taken and the plate was incubated for 6 h. Samples were taken at 2, 4 and 6 h according to the procedure below.

180 µL of cold, sterile PBS were added to each well of a 96-well plate. 20 µL of bacterial culture were added to the first row and a serial dilution was performed in the 96-well plate from top to bottom at a 1:10 ratio, transferring 20 µL into 180 µL at each step.

A multichannel pipette was used to transfer 10 µL from three rows containing the estimated range of sensible dilutions and place them on one half of the centerline of an agar plate. The process was repeated with another replicate/sample for the other half of the agar plate. The plate was tilted back and forth to achieve a uniform strip of bacterial suspension on the agar surface.

At the end of the day, the plates were placed in an incubator at 37 °C and incubated for 12 h without shaking. The next day, colonies were counted, and the results were statistically analyzed using Microsoft Excel and GraphPad Prism v10.0.1.

Resistance Development Assay by Serial Passage The resistance development assay by serial passage was conducted based on previously published procedures by Hübner *et al.* and Silverman *et al.*, with minor modifications.[S13,S14]

An overnight culture of *E. coli* K12 in LB medium was adjusted to 0.25 McFarland units with fresh medium. The suspension was then diluted 1:100 in a 12-well plate (total volume of 500 µL per well) into LB medium supplemented with 12.5 mM $NaHCO_3$, and containing various concentrations of **D8** or nitrofurantoin (0.25, 0.5, 1, 2 and 4 × MIC, 1% DMSO final concentration), as well as 1% DMSO as growth control.

Bacteria were incubated at 37 °C and 200 rpm and passaged every 20–24 h. The culture from the highest concentration allowing visible growth was adjusted to 0.25 McFarland units with fresh medium and subsequently diluted 1:100 into LB medium suppplemented with $NaHCO_3$ and the corresponding concentrations of the antibiotic as above. If a shift in MIC was observed, the compound concentrations were adjusted accordingly for the next passage, as far as the solubility permitted.

This serial passaging was repeated for six consecutive days and carried out in three

independent biological replicates. As a negative control, bacteria were passaged daily in LB medium containing 1% DMSO without any antimicrobial agent. For this, the growth control culture was adjusted daily to 0.25 McFarland units and diluted 1:100 into LB medium supplemented with 12.5 mM $NaHCO_3$ and 1% DMSO.

After six passages, a full MIC assay was performed according to the standard procedure described above, determining the MIC of both **D8** and nitrofurantoin in each biological replicate of the serial passage of **D8**, nitrofurantoin and DMSO.

Cytotoxicity Determination *via* Metabolic Activity Measurement (MTT Assay)
Cytotoxicity was assessed in human embryonic kidney 293 cells (HEK 293) in 96-well plates (Transparent Nunc 96-well flat bottom, cell culture treated, *Thermo Fisher Scientific*) coated with poly-L-lysine. To coat the plates, a 0.01 % aqueous solution of poly-L-lysine was used (50 µL per well). The solution was evenly distributed by tilting the plate, followed by a 10 min incubation at room temperature. The coating solution was then aspirated, the wells were rinsed with 200 µL PBS and then left to dry without lid for 15 min.

The cells were cultured in a T175 flask in high glucose Dulbecco's Modified Eagle's Medium (DMEM), supplemented with 10 % FBS and 2 mM L-glutamine. After removing the medium, cells were washed with PBS and detached by incubating with 2 mL Accutase® (*Sigma-Aldrich*) for 5 min at 37 °C, followed by tapping the flask. 8 mL of fresh medium were added to wash the cells from the flask wall. The cell suspension was gently mixed and transferred to a 15 mL Falcon tube, centrifuged at 500 rpm for 5 min, the supernatant removed, and the pellet resuspended in 10 mL fresh medium.

The concentration of viable cells was determined using trypan blue stain, a Neubauer counting chamber and a stereomicroscope. HEK 293 cells were seeded at a density of 4000 cells per well in 100 µL medium on the poly-L-lysine-coated 96-well plates. To prevent edge effects, wells in rows A and H and columns 1 and 12 were filled with 200 µL medium or PBS. The cells were incubated overnight at 37 °C and 5 % CO_2.

For compound treatment, a dilution series of the compound was prepared from a DMSO stock in DMEM without FBS, ensuring a final DMSO concentration of 1 %. Medium was carefully removed from the wells without disturbing the cell layer, and 100 µL of the compound dilutions or DMSO control were added to each well. Plates were incubated for 24 h at 37 °C and 5 % CO_2.

After incubation, 20 µL of 3-(4,5-dimethyl-2-thiazolyl)-2,5-diphenyl-2*H*-tetrazolium bromide solution (MTT, 5 mg mL^{-1} in PBS) were added to each well using a stepper pipette. The plate was incubated in the dark for 1 h at 37 °C and 5 % CO_2. Following incubation, the medium was carefully removed, and formazan crystals were solubilized in 200 µL DMSO per well. Plates were placed on a rocker at 300 rpm for 10 min.

Absorbance at 570 nm (formazan) and 630 nm (background) was measured using a plate reader (Tecan Infinite® 200 Pro M Nano). The assay was performed in $n = 3$ biologically independent replicates with three technical replicates each. IC_{50} values (concentration at which 50 % viability is reached) were determined using GraphPad Prism v10.0.1. The background was subtracted, the absorption values of technical replicates were averaged and the resulting values were normalized to the upper and lower asymptote of a nonlinear fit of the data ([Inhibitor] vs. response – variable slope (four parameters)) for each compound tested, followed by re-fitting a nonlinear regression to the normalized data ([Inhibitor] vs. normalized response – variable slope) according to a protocol by Krebs *et al.*.[S4]

Cyclic Voltammetry Measurements Cyclic voltammetry experiments were conducted in 20 mL septum-sealed screw-cap vials under an argon atmosphere, using a Palmsens4 potentiostat. The electrochemical setup consisted of a 1 mm diameter glassy carbon (GC) disk working electrode, a 2 mm platinum rod as the counter electrode, and a leakless Ag/AgCl reference electrode (3.4 M KCl, EDAQ, model ET-072). The electrolyte for each measurement was freshly prepared by mixing 4 mL of a 0.1 M aqueous Na_2SO_4 solution with 1 mL of DMF to aid solubility of the organic compounds, affording a 0.08 M Na_2SO_4 electrolyte solution. All

measurements were recorded at a scan rate of 0.2 V/s and the data is reported vs. Ag/AgCl (3.4 M). For external calibration of the reference electrode in this electrolyte, additionally a cyclic voltammogram of the ferrocenium / ferrocene couple was measured, showing a half-wave potential of 0.20 V/s vs. the leakless Ag/AgCl reference electrode.

Mass Spectrometry

Full Proteome Analysis The procedure was adapted from Schum *et al.*[S15] Overnight cultures of *E. coli* K-12 were diluted to an initial $OD_{600} = 0.05$ in 5.5 mL LB medium and shaken for 2 h at 37 °C and 200 rpm. Optical densities were then recorded (1:1 dilution, BioPhotometer model 6131, *Eppendorf*). Upon reaching $OD_{600} \approx 0.5$, the cultures were treated with either DMSO or one of the following antibiotics: nitrofurantoin (250 µM), nitrofurazone (500 µM), **D8** (125 µM), tetracycline (6.25 µM) or ampicillin (31.25 µM). Cells were cultivated for a further 1 h at 37 °C and 200 rpm, after which all cultures were diluted with PBS to match the lowest OD_{600}.

1 mL of each normalised suspension was pelleted (6000 g, 5 min, 4 °C) and washed once with ice-cold PBS. Pellets were resuspended in 150 µL PBS containing 0.5 % SDS and 1 % Triton X-100. Cells were disrupted by a 10 s sonication pulse at 30 % intensity (Sonopuls HD 2070, *Bandelin*) followed by bead beating in bead-mill tubes (0.1 mm zirconia beads) for three cycles of 30 s at 6500 rpm with 30 s cooling intervals inbetween (Precellys 24 Homogenizer, *Bertin Technologies*). Lysates were centrifuged at 10 000 g for 10 min, transferred to new microcentrifuge tubes, and centrifuged at 21 000 g for 30 min (RT). The final supernatants were transferred to new LoBind tubes (*Eppendorf*) and the protein concentrations were quantified by BCA assay (Roti Quant, *Carl Roth*).

Equal amounts of protein (50 µg in 80 µL) were transferred to a V-bottom 96-well polypropylene plate (Greiner, cat. 651201). For reduction/alkylation, 3 µL of a 1:2 mixture of tris(2-carboxyethyl)phosphine (TCEP) and iodoacetamide (IAA) (each 500 mM) were added, incubated for 15 min at 950 rpm (RT), and excess IAA was quenched with 2 µL 500 mM DTT.

A 1:1 mix of hydrophobic and hydrophilic carboxylate-coated magnetic beads (*Cytiva*, cat. 65152105050250 and 45152105050250), washed three times with H_2O, was added (10 µL per well) followed by 150 µL EtOH to precipitate proteins.

All further liquid-handling steps were executed on a Hamilton Microlab Prep robot. After 5 min of shaking (500 rpm, RT), beads were captured on a 96-ring magnet (Magnum FLX, *Alpaqua*), and supernatants were withdrawn slowly at $20\,\mu L\,s^{-1}$ to prevent bead loss. Beads were washed three times with 180 µL 80 % EtOH and once with 180 µL ACN, with off-magnet shaking between wash steps (1 min, 800 rpm, RT).

Proteins were digested overnight at 37 °C in 100 µL 50 mM TEAB containing 1 µL sequencing-grade trypsin (trypsin:protein 1:100, $0.5\,\mu g\,\mu L^{-1}$; *Promega*) while shaking at 800 rpm under a heated lid with a tightly sealed plate. Peptides were then eluted with 50 µL 3 % formic acid, desalted on two-disk styrenedivinylbenzene-reverse phase sulfonate (SDB-RPS) StageTips (Empore, *3M*), which were equilibrated with 150 µL wash buffer 1 (1 % TFA in isopropanol).[S16] Samples were loaded (10 min, 500 g), washed with buffer 1 (30 min, 800 g) and buffer 2 (0.2 % TFA in H_2O; 30 min, 800 g). Peptides were eluted using 50 µL elution buffer (1 % NH_3, 80 % ACN) by centrifugation (5 min at 300 g and then 800 g) and dried in a centrifugal evaporator (Concentrator Plus, *Eppendorf*). Dried peptides were reconstituted in 1 % formic acid and 3 µL were injected on a *Bruker* timsTOF Pro operated in DIA mode. All conditions were examined in four independent biological replicates.

LC-MS/MS Measurements timsTOF Pro The procedure was adapted from Schum et al.[S15] Peptide separation and mass spectrometry were performed using an UltiMate 3000 nano-HPLC system (*Dionex*) coupled to a timsTOF Pro instrument (*Bruker*) via a CaptiveSpray nano-electrospray ion source and a Sonation column oven. Samples were first loaded onto a trap column (Acclaim PepMap 100 C18, 75 µm ID × 2 cm, 3 µm particles, *Thermo Fisher Scientific*) and washed for 7 min using solvent A (0.1 % formic acid in water) at a flow rate of $5\,\mu L\,min^{-1}$.

The peptides were then eluted onto a separation column (Aurora C18, 25 cm × 75 µm, 1.7 µm particle size, *IonOpticks*) and separated with a gradient of solvent B (0.1 % formic acid in ACN) at a constant flow of 400 nL min^{-1}. The elution profile consisted of: 5–28 % B over 28 min, 28–40 % B over the next 6 min, and finally a high organic wash at 95 % B for 6 min before re-equilibration (5 % B for 10 min).

The mass spectrometer was operated in dia-PASEF mode. Ion mobility separation was conducted using a dual TIMS analyzer with equal accumulation and ramp times of 100 ms, covering a $1/K_0$ ion mobility range from 0.60 V s cm^{-2} to 1.60 V s cm^{-2} for MS1 scans. Fragmentation was carried out over an m/z range of 400–1201 using an ion mobility window of 0.60 V s cm^{-2} to 1.43 V s cm^{-2}.

Each dia-PASEF scan included two ion mobility isolation windows with 26 m/z width. The full mass range was covered using 32 windows with 1 m/z overlaps, yielding 16 dia-PASEF scans per MS1 cycle and an overall cycle time of approximately 1.80 s (see Tab. S10). Collision energy was decreased linearly from 59 eV at $1/K_0$ = 1.3 V s cm^{-2} down to 20 eV at $1/K_0$ = 0.85 V s cm^{-2}. TIMS elution voltage calibration was performed using three reference ions (m/z 622, 922, and 1222) from the Agilent ESI-L Tuning Mix, which were spiked directly into the CaptiveSpray inlet filter to obtain accurate reduced ion mobility coefficients ($1/K_0$).

Table S10: DIA-PASEF scan windows including ion mobility range ($1/K_0$) and scan width (m/z).

**MS Type**	**Scan**	**Start IM** [$1/K_0$]	**End IM** [$1/K_0$]	**Start Mass** [m/z]	**End Mass** [m/z]
MS1	0	0.60	1.60	100	1700
dia-PASEF	1	0.90	1.20	800	826
dia-PASEF	1	0.60	0.90	400	426
dia-PASEF	2	0.92	1.22	825	851
dia-PASEF	2	0.62	0.92	425	451
dia-PASEF	3	0.93	1.23	850	876
dia-PASEF	3	0.63	0.93	450	476
dia-PASEF	4	0.95	1.25	875	901
dia-PASEF	4	0.65	0.95	475	501
dia-PASEF	5	0.96	1.26	900	926
dia-PASEF	5	0.66	0.96	500	526
dia-PASEF	6	0.98	1.28	925	951

MS Type	Scan	Start IM [1/K$_0$]	End IM [1/K$_0$]	Start Mass [m/z]	End Mass [m/z]
dia-PASEF	6	0.68	0.98	525	551
dia-PASEF	7	0.99	1.29	950	976
dia-PASEF	7	0.69	0.99	550	576
dia-PASEF	8	1.01	1.31	975	1001
dia-PASEF	8	0.71	1.01	575	601
dia-PASEF	9	1.02	1.32	1000	1026
dia-PASEF	9	0.72	1.02	600	626
dia-PASEF	10	1.04	1.34	1025	1051
dia-PASEF	10	0.74	1.04	625	651
dia-PASEF	11	1.06	1.36	1050	1076
dia-PASEF	11	0.76	1.06	650	676
dia-PASEF	12	1.07	1.37	1075	1101
dia-PASEF	12	0.77	1.07	675	701
dia-PASEF	13	1.09	1.39	1100	1126
dia-PASEF	13	0.79	1.09	700	726
dia-PASEF	14	1.10	1.40	1125	1151
dia-PASEF	14	0.80	1.10	725	751
dia-PASEF	15	1.12	1.42	1150	1176
dia-PASEF	15	0.82	1.12	750	776
dia-PASEF	16	1.13	1.43	1175	1201
dia-PASEF	16	0.83	1.13	775	801

Data Analysis of timsTOF Pro Measurements The MS data was first processed using DIA-NN[S17] in library-free mode. For library generation, the UniProt reference proteome for *E. coli* K12 (proteome ID :UP000000625, taxon ID: 8333, downloaded on 2024/07/16) was used. The DIA-NN configuration file was uploaded on the repositories referenced in the data availability section.

The resulting LFQ intensities were then processed using Perseus software[S18] (version 2.1.3.0). LFQ intensities were $\log_2$-transformed, and samples were grouped according to treatment groups. Then, protein groups were filtered to retain those with at least 3 valid values in one group. For statistical analysis, two-sample Student's t-tests with permutation-based multiple testing correction (FDR = 0.05) were used, always comparing the antibiotic-treated group with the corresponding DMSO control group. Resulting tables were exported, and analyzed using Python (Pandas version 2.2.3). All Student's t-test difference values with

correspondent FDR-values < 0.05 were set to 0. Then, the correlation between the Student's t-test difference vectors were calculated using Pandas Pearson correlation implementation. The mapping of the antibiotics to their controls is shown in Tab. S11.

Table S11: Mapping between antibiotic-treated samples and their matched DMSO controls.

**Antibiotic treatment**	**Sample ID**	**Control ID**
Nitrofurantoin	31	DF
Nitrofurazone	58	DK
**D8**	65	DL
Tetracycline	15	DH
Ampicillin	Amp	DI

NMR Spectra

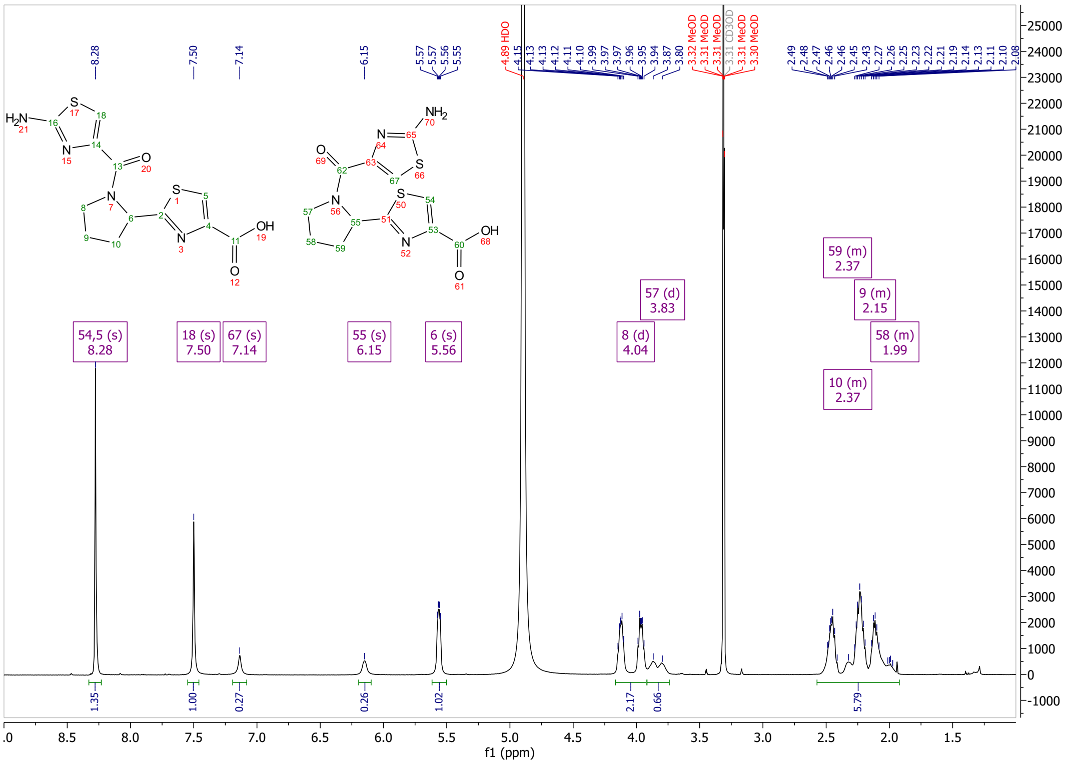

^{1}H-NMR spectrum of **1**.

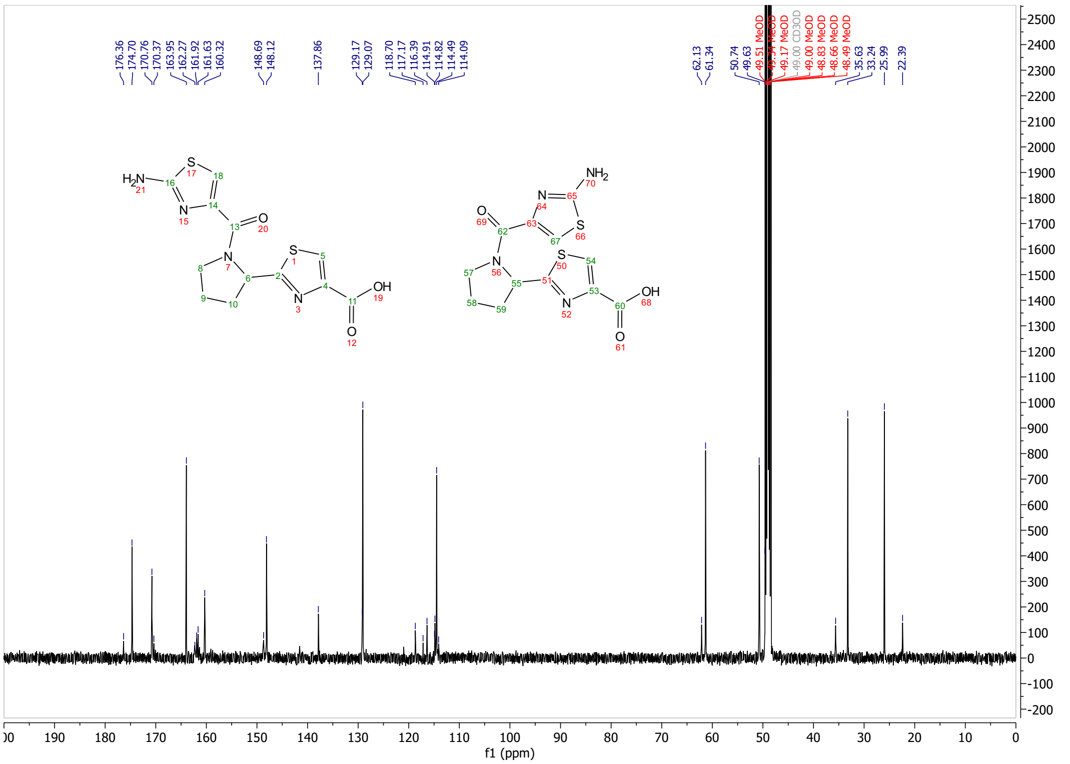

^{13}C-NMR spectrum of **1**.

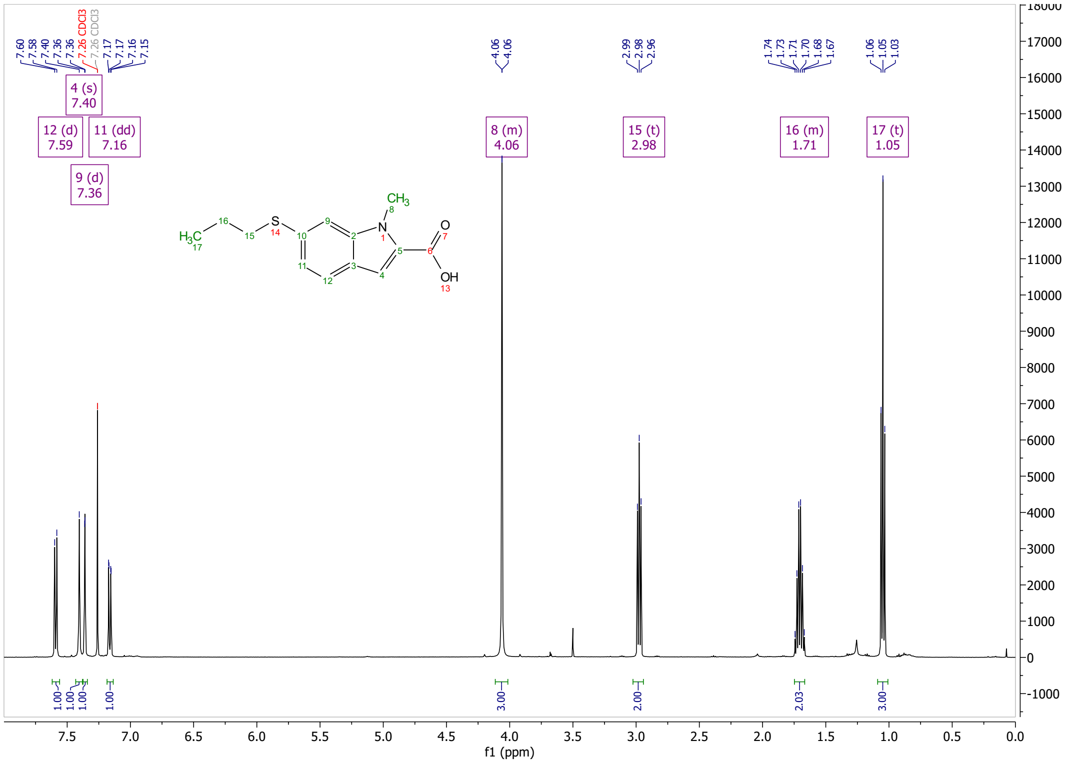

^{1}H-NMR spectrum of **2**.

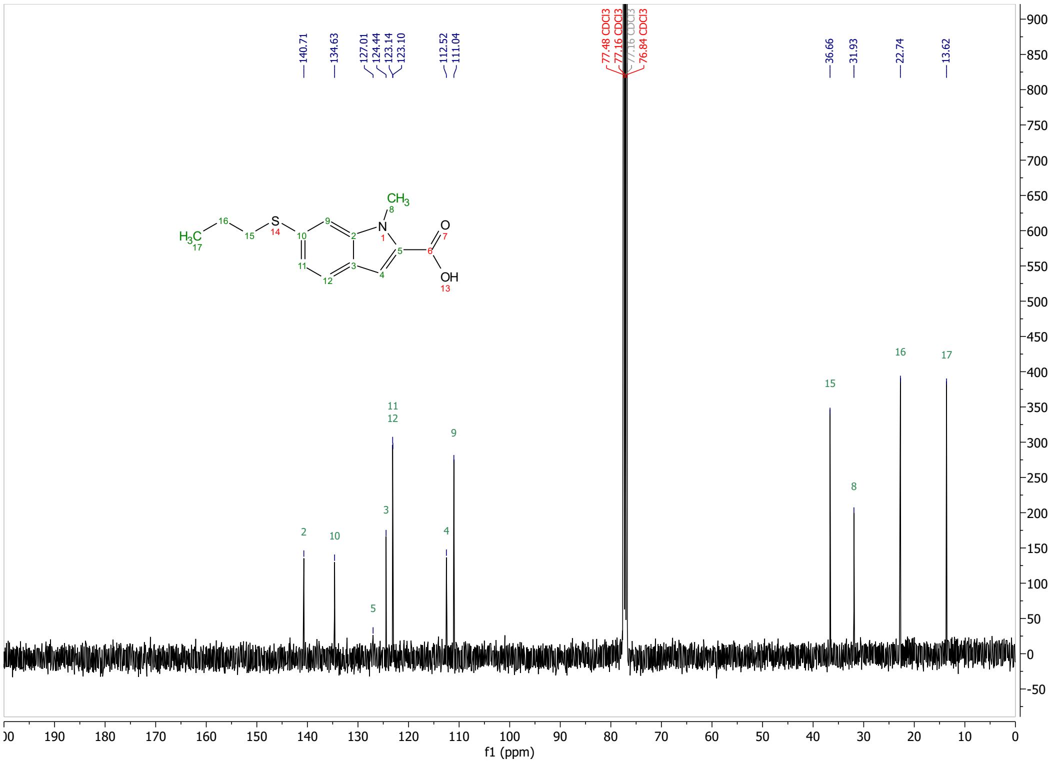

^{13}C-NMR spectrum of **2**.

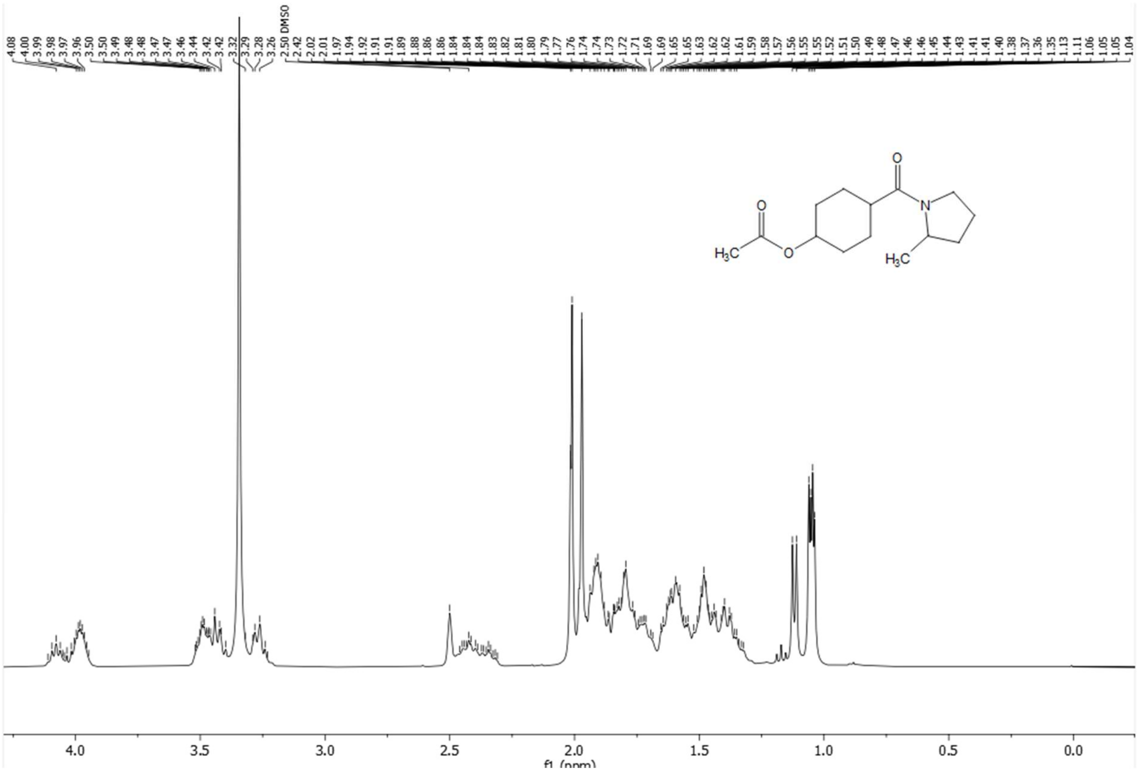

^{1}H-NMR spectrum of **3**.

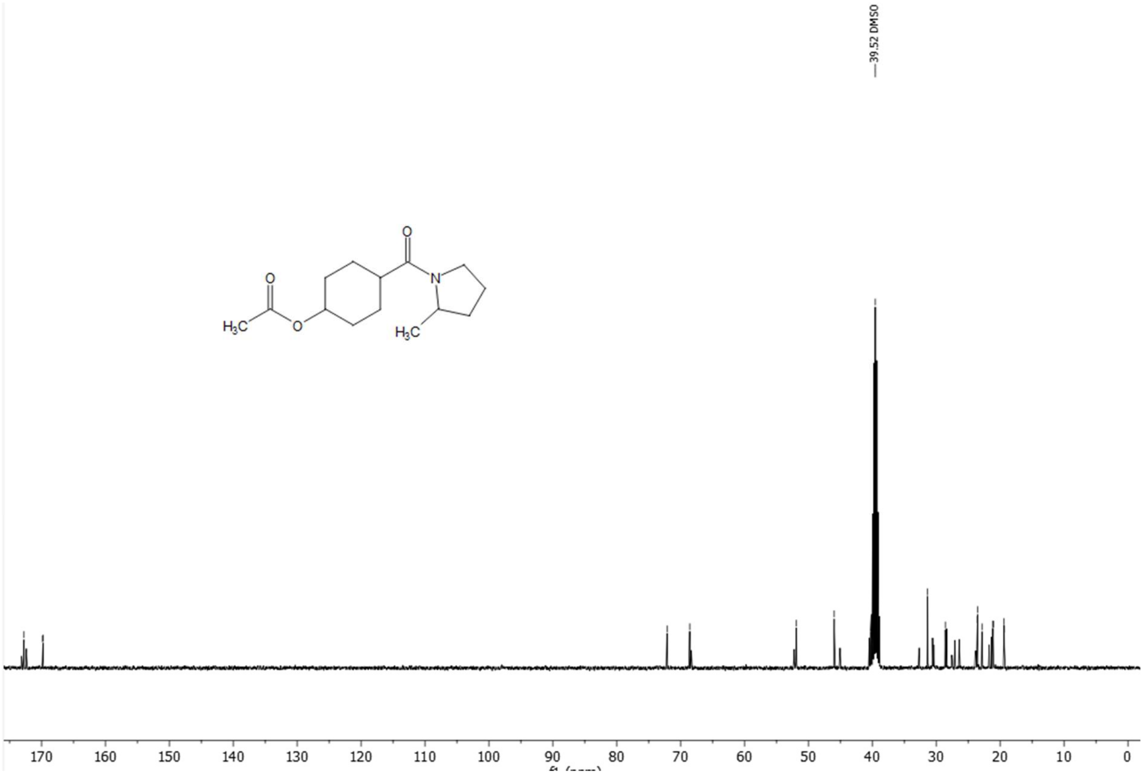

^{13}C-NMR spectrum of **3**.

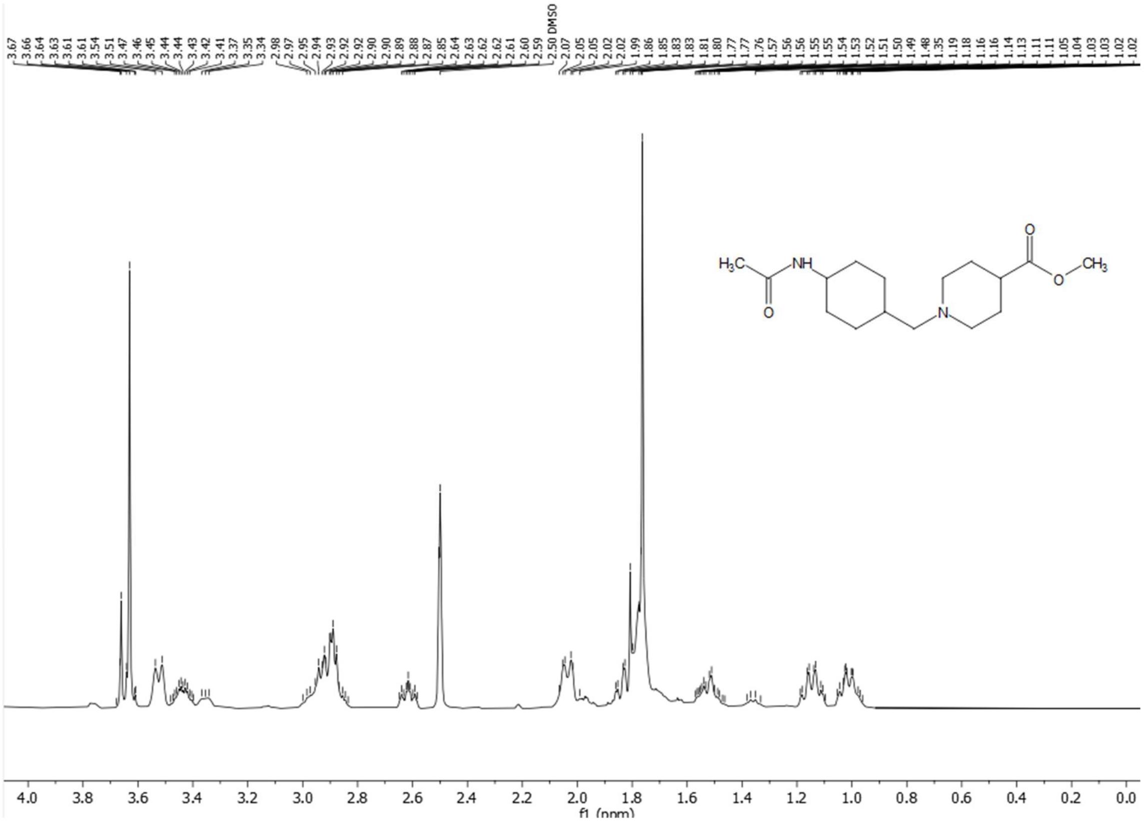

^{1}H-NMR spectrum of **4**.

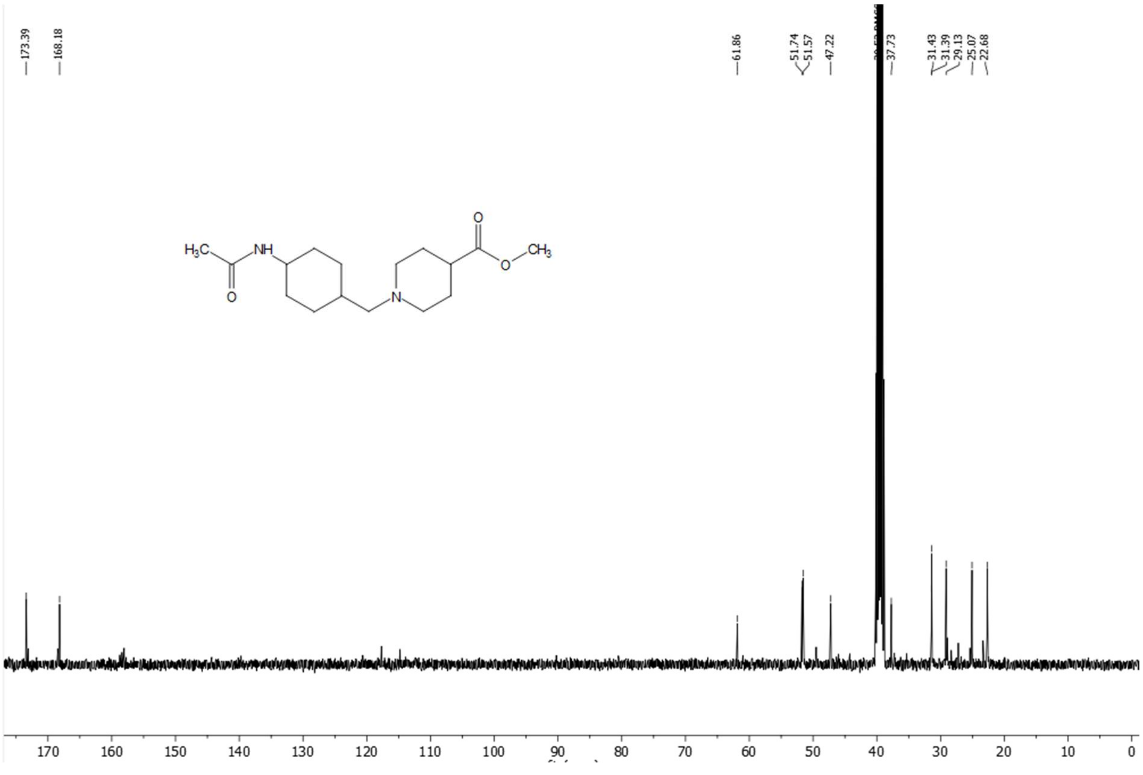

^{13}C-NMR spectrum of **4**.

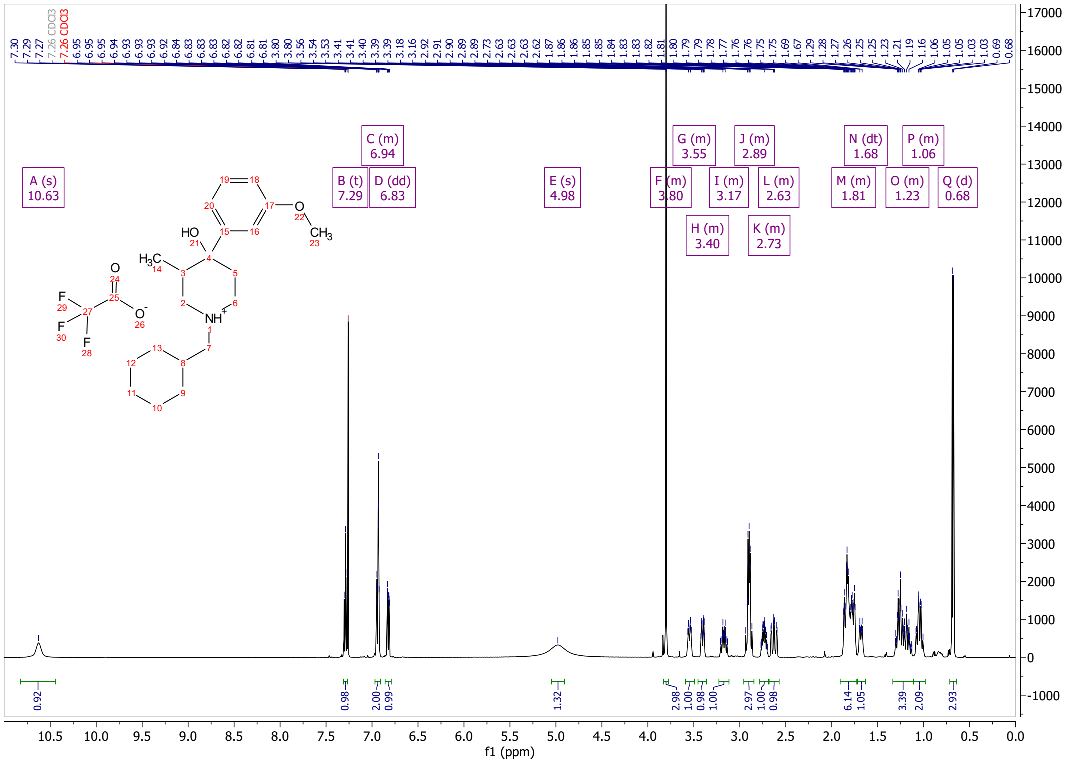

^{1}H-NMR spectrum of **5**.

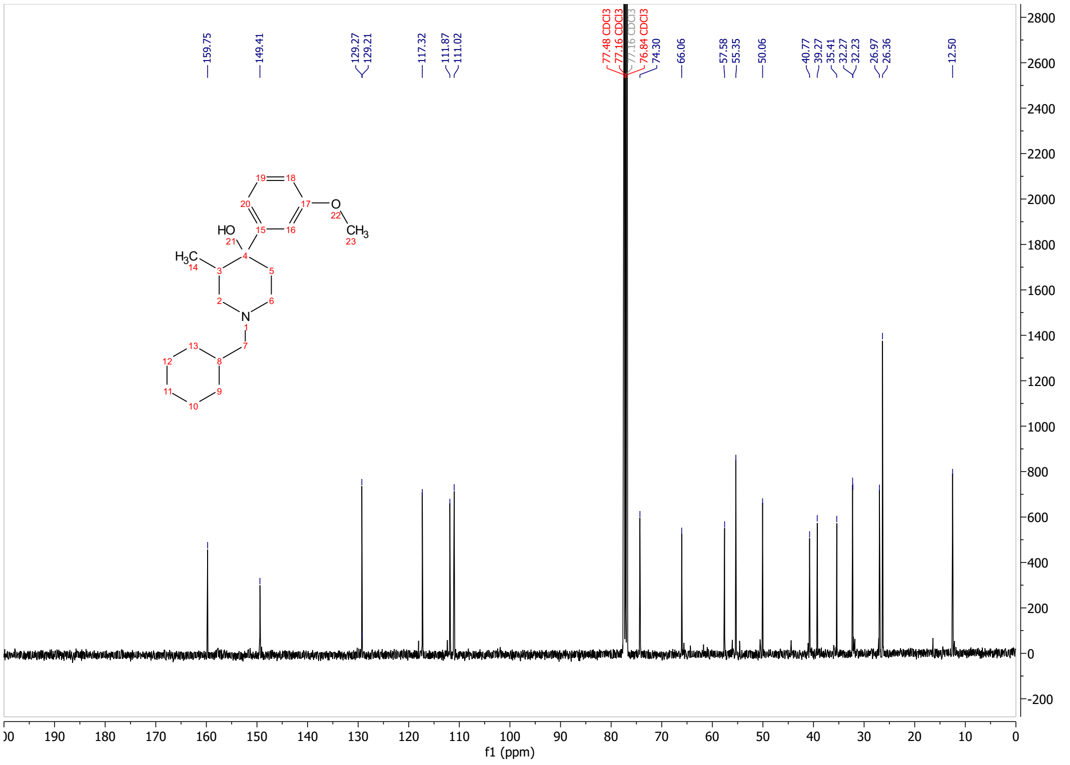

^{13}C-NMR spectrum of **5**.

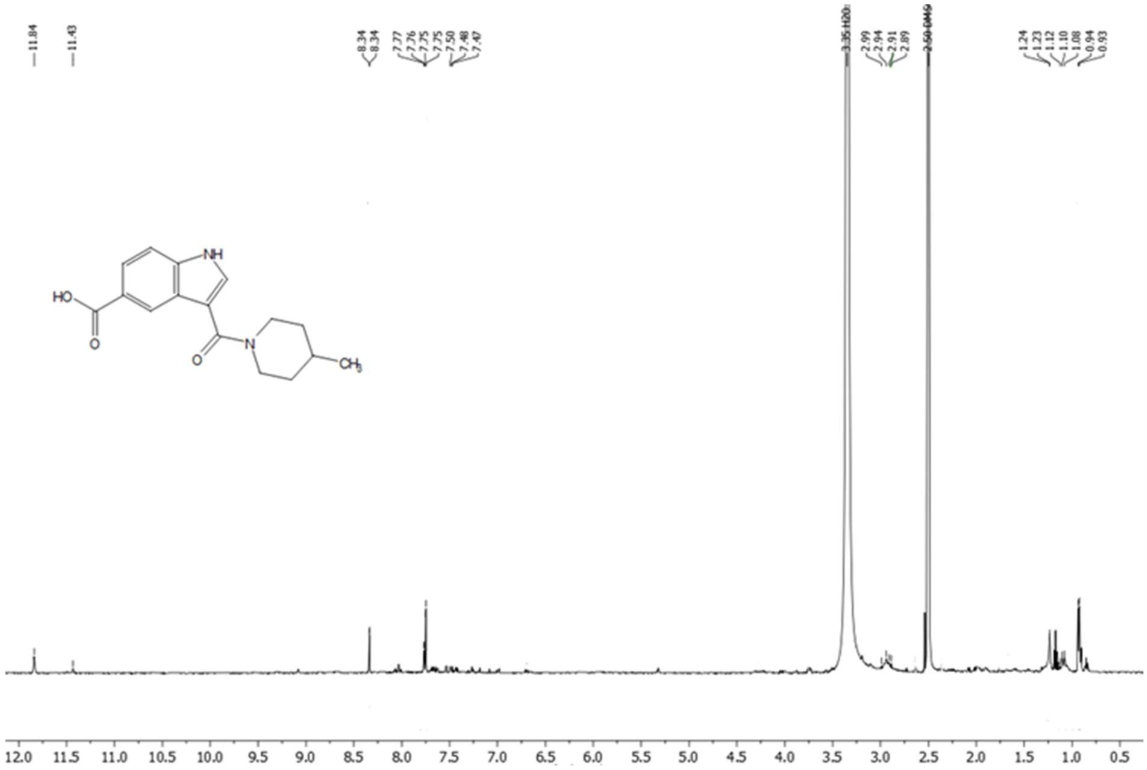

^{1}H-NMR spectrum of **6**.

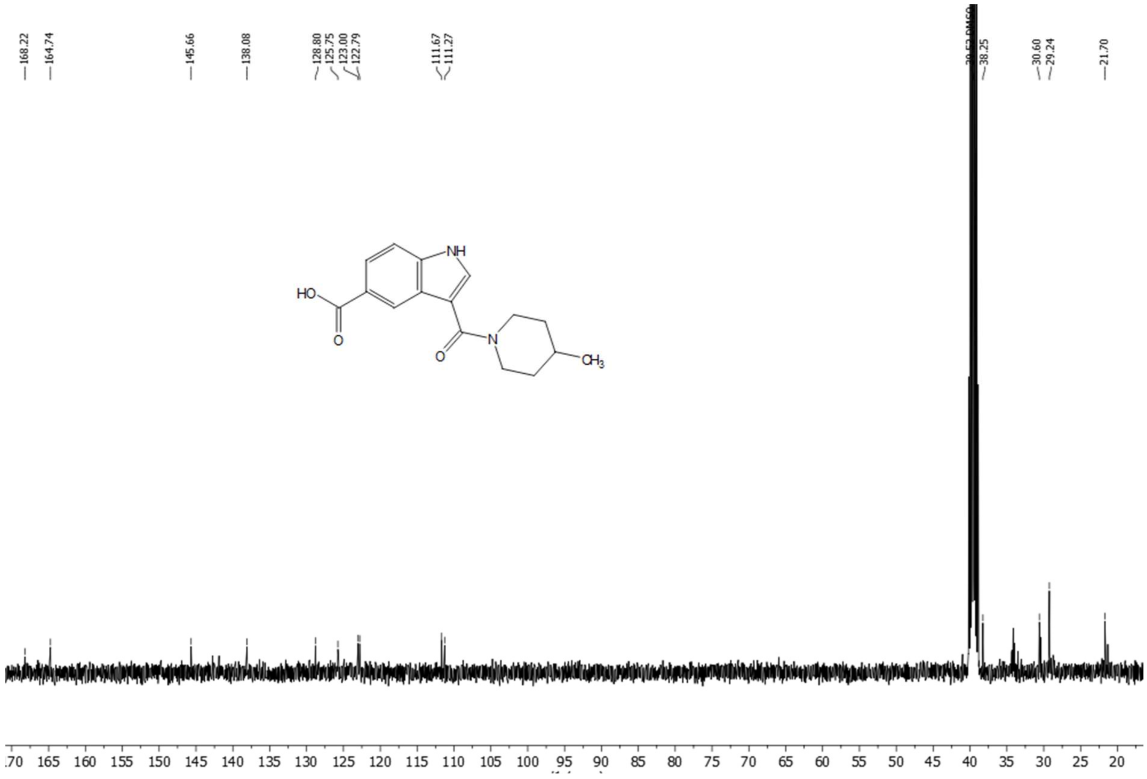

^{13}C-NMR spectrum of **6**.

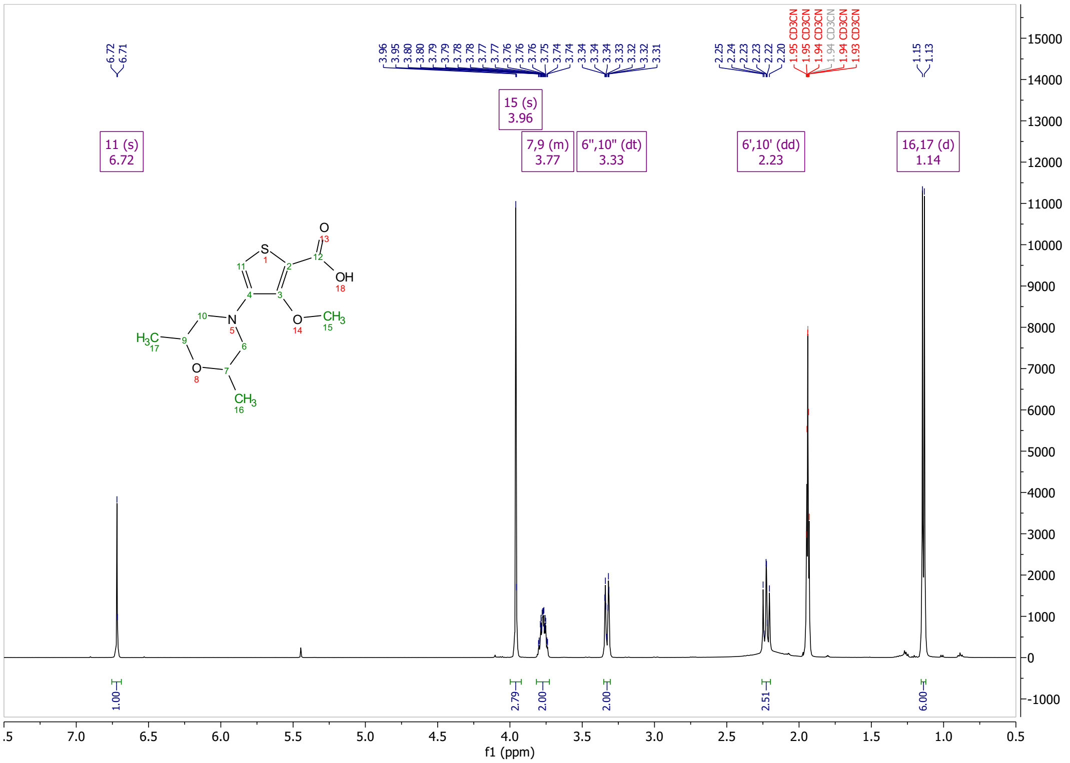

^{1}H-NMR spectrum of **7**.

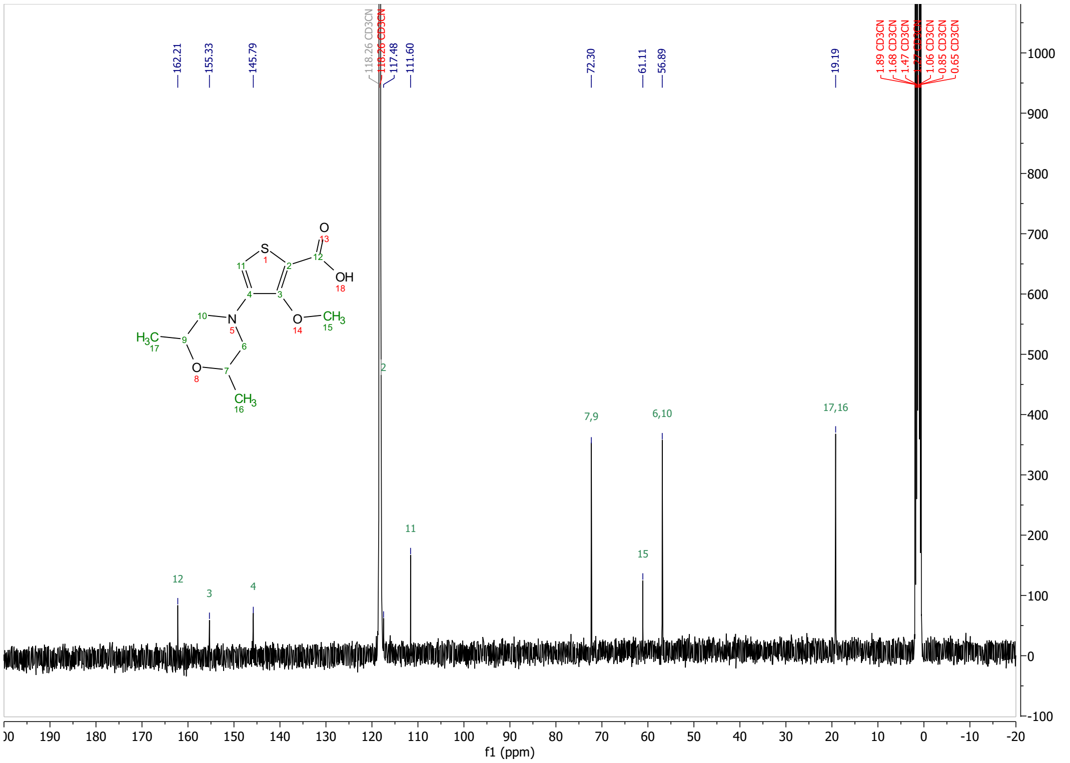

^{13}C-NMR spectrum of **7**.

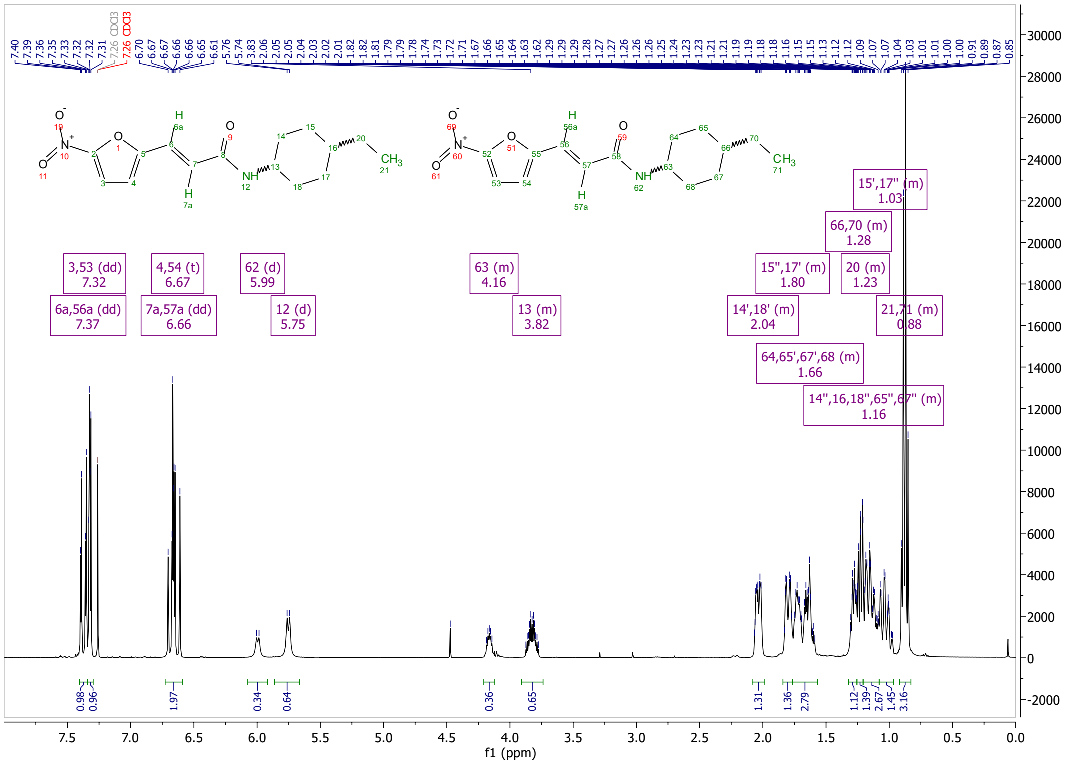

^{1}H-NMR spectrum of **8**.

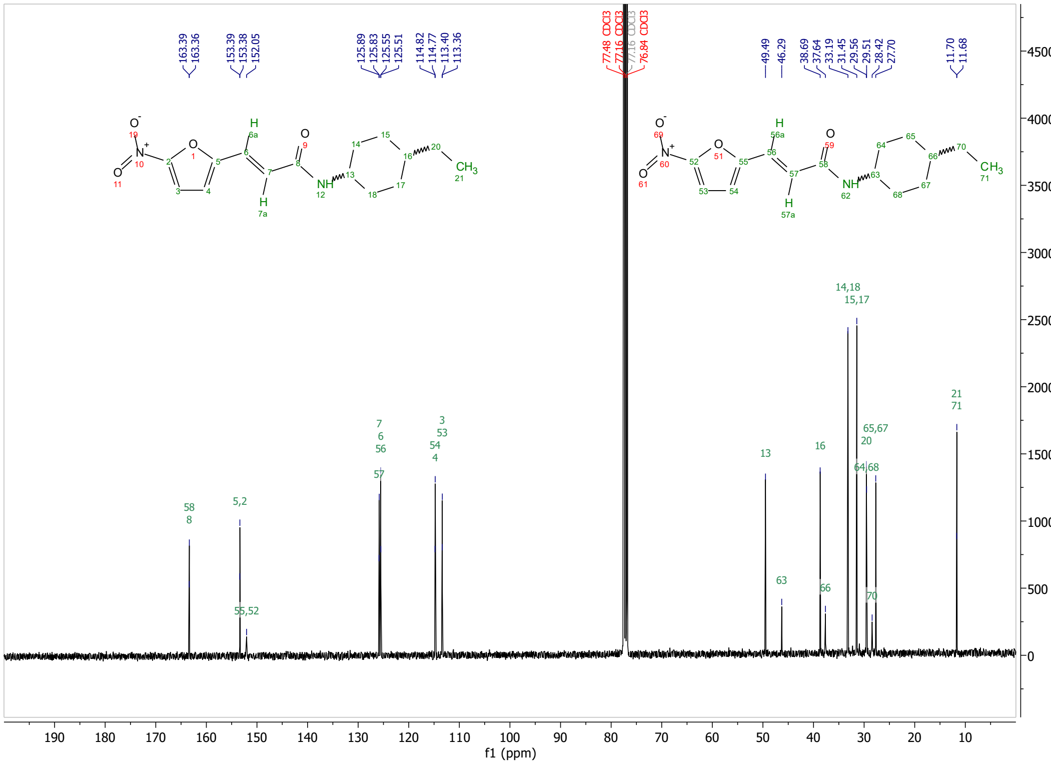

^{13}C-NMR spectrum of **8**.

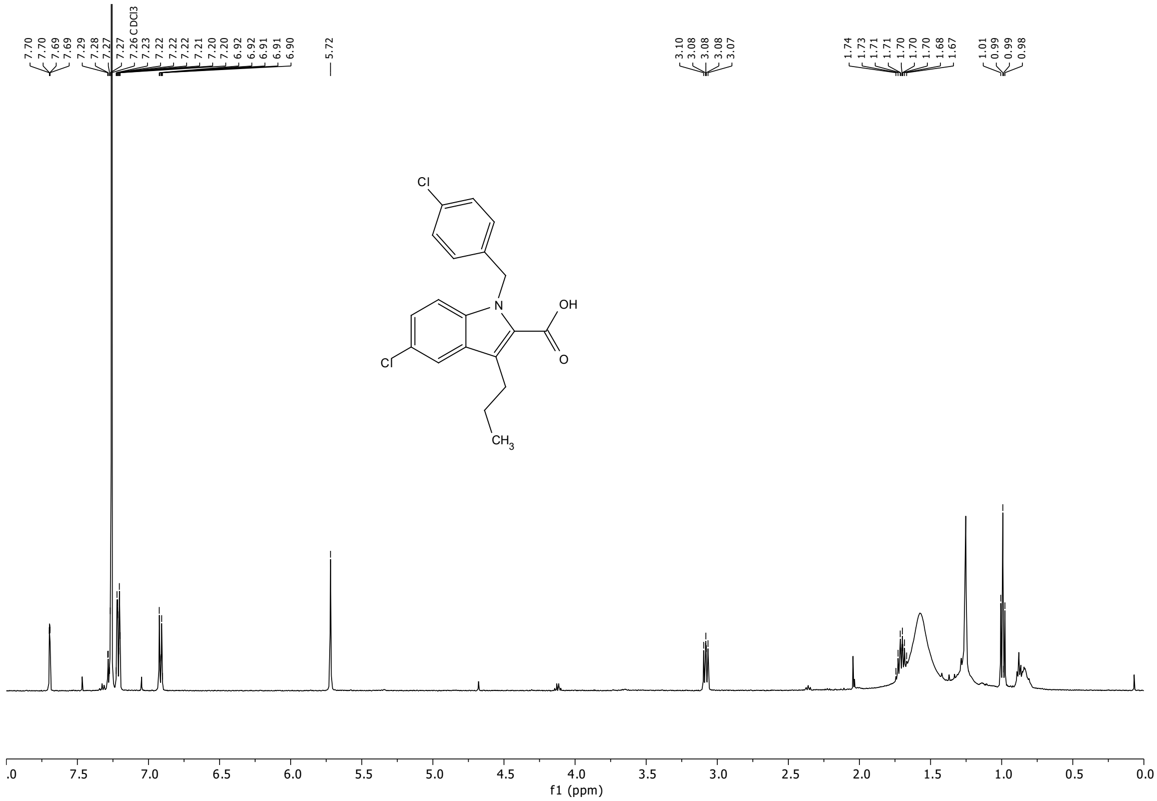

^{1}H-NMR spectrum of **9**.

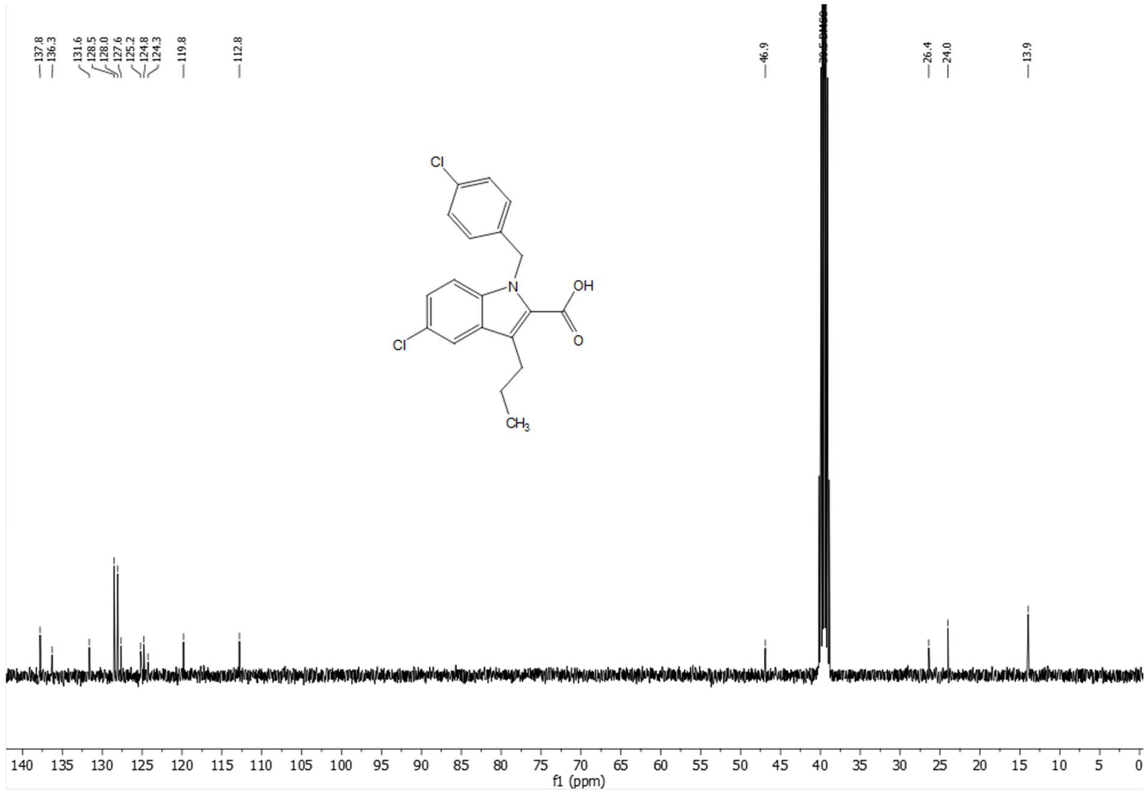

^{13}C-NMR spectrum of **9**.

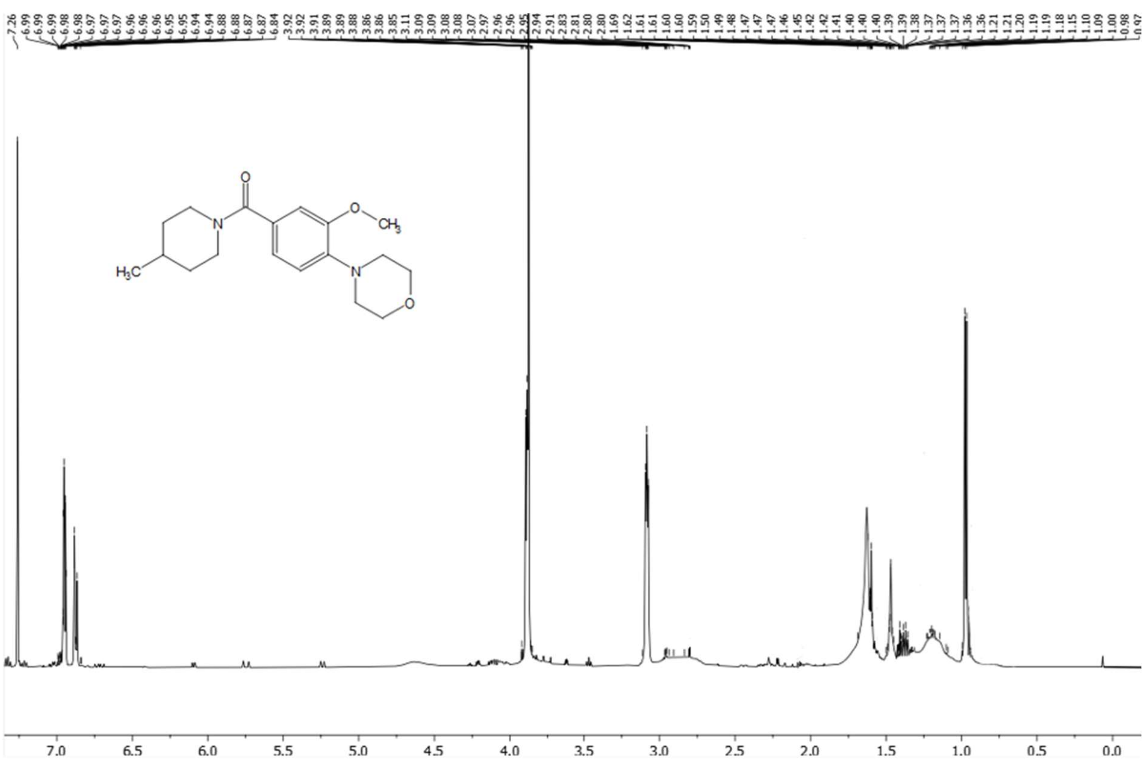

^{1}H-NMR spectrum of **10**.

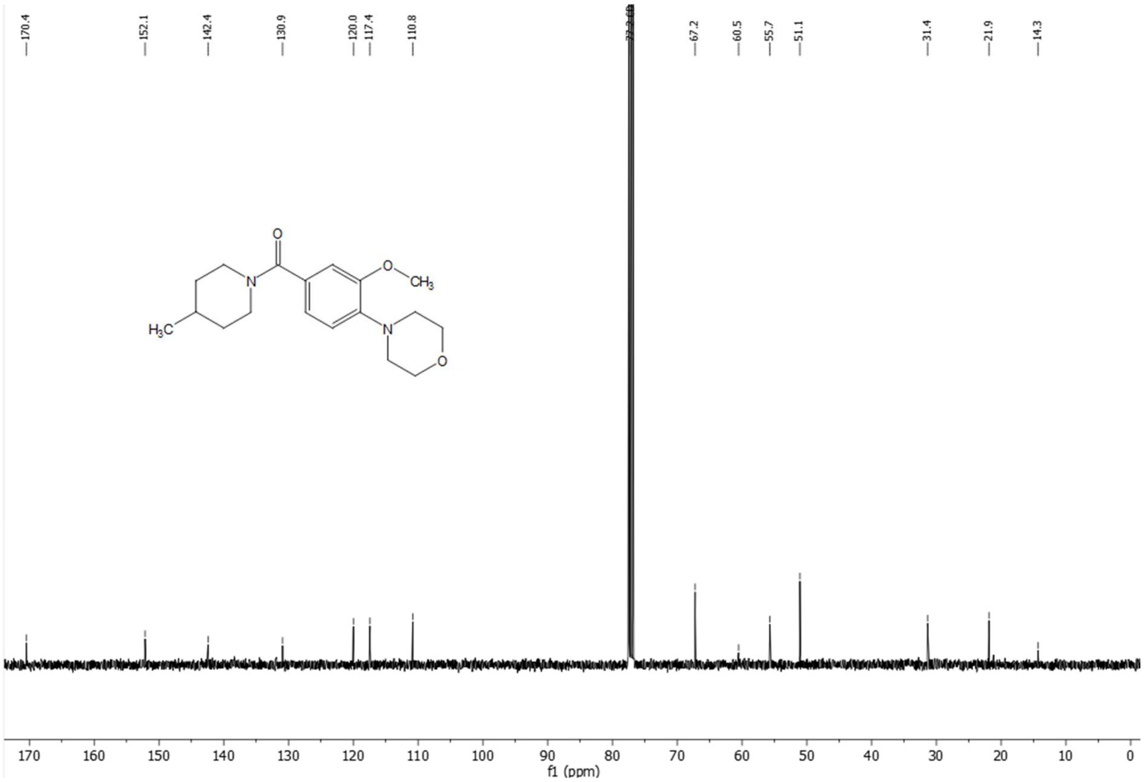

^{13}C-NMR spectrum of **10**.

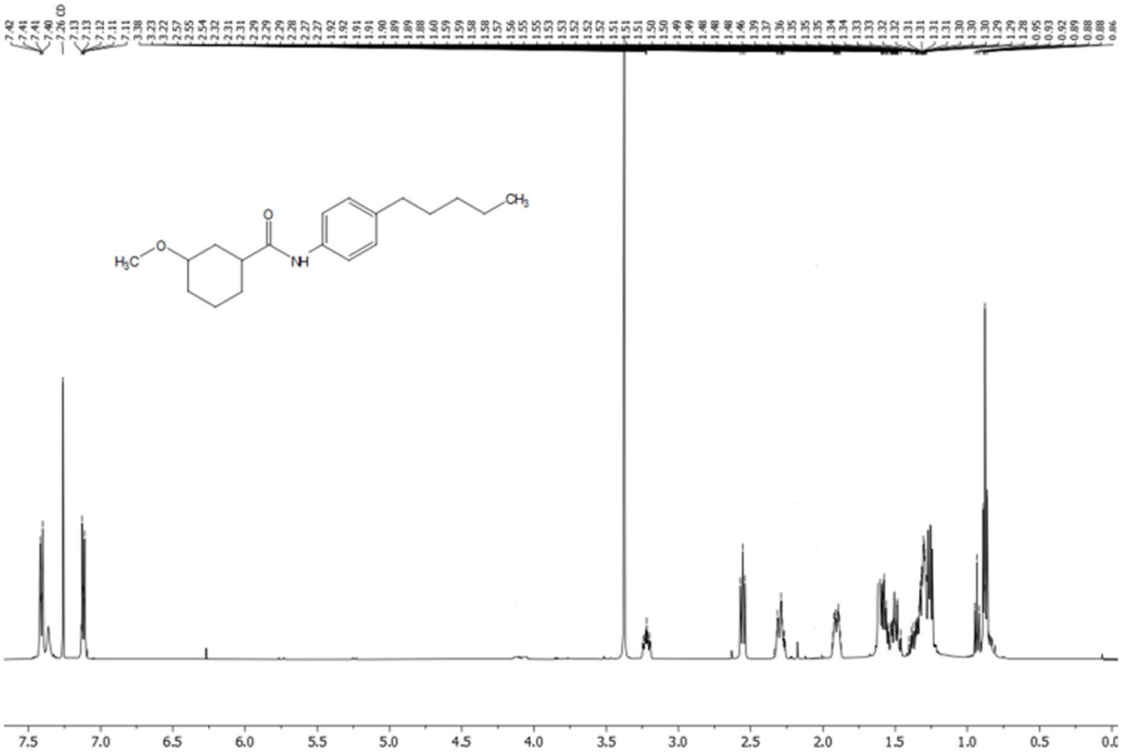

^{1}H-NMR spectrum of **11**.

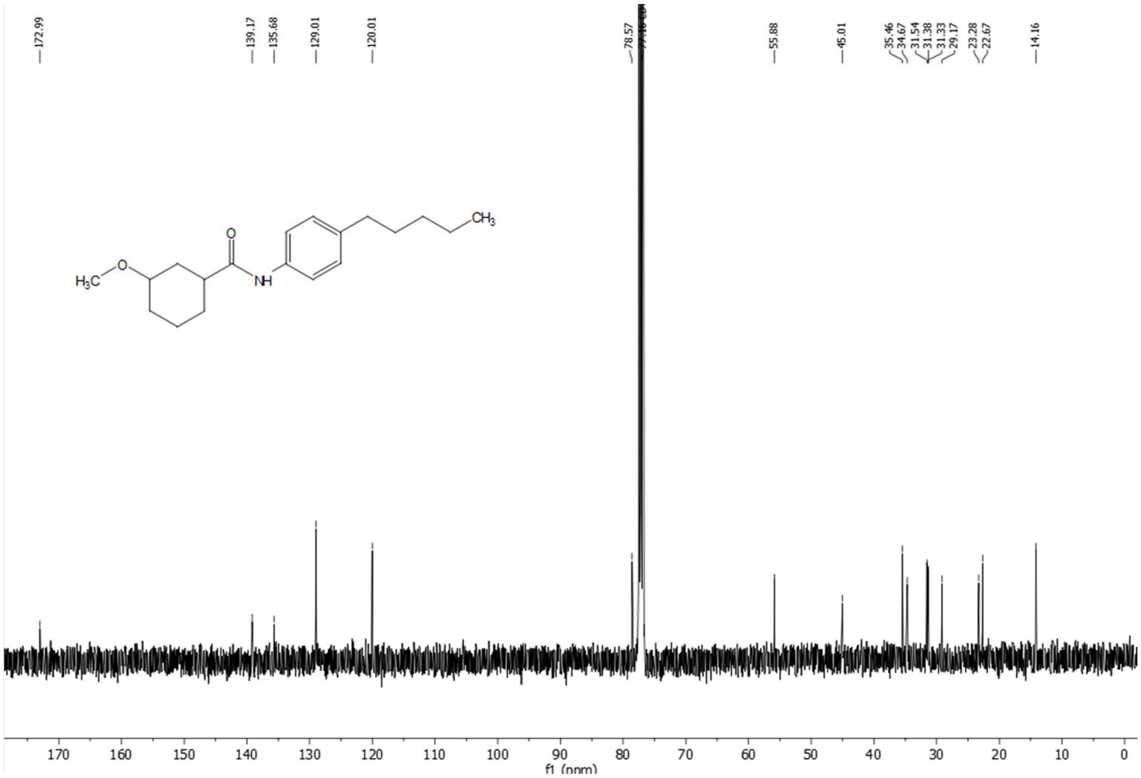

^{13}C-NMR spectrum of **11**.

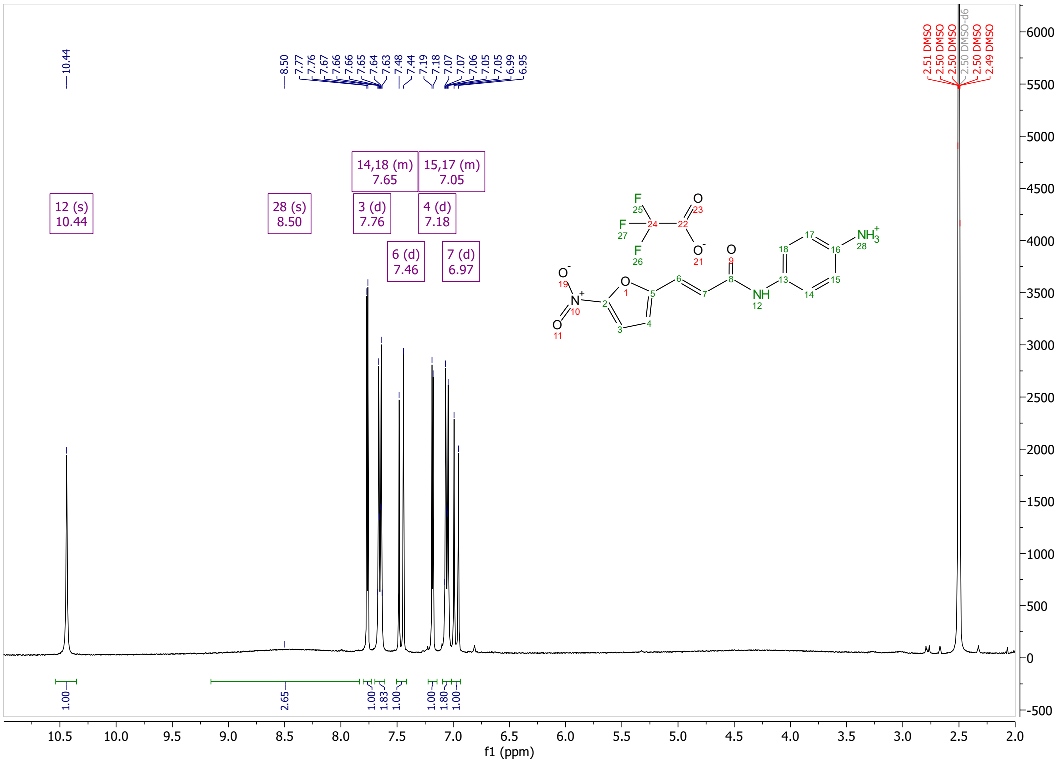

^{1}H-NMR spectrum of **D8**.

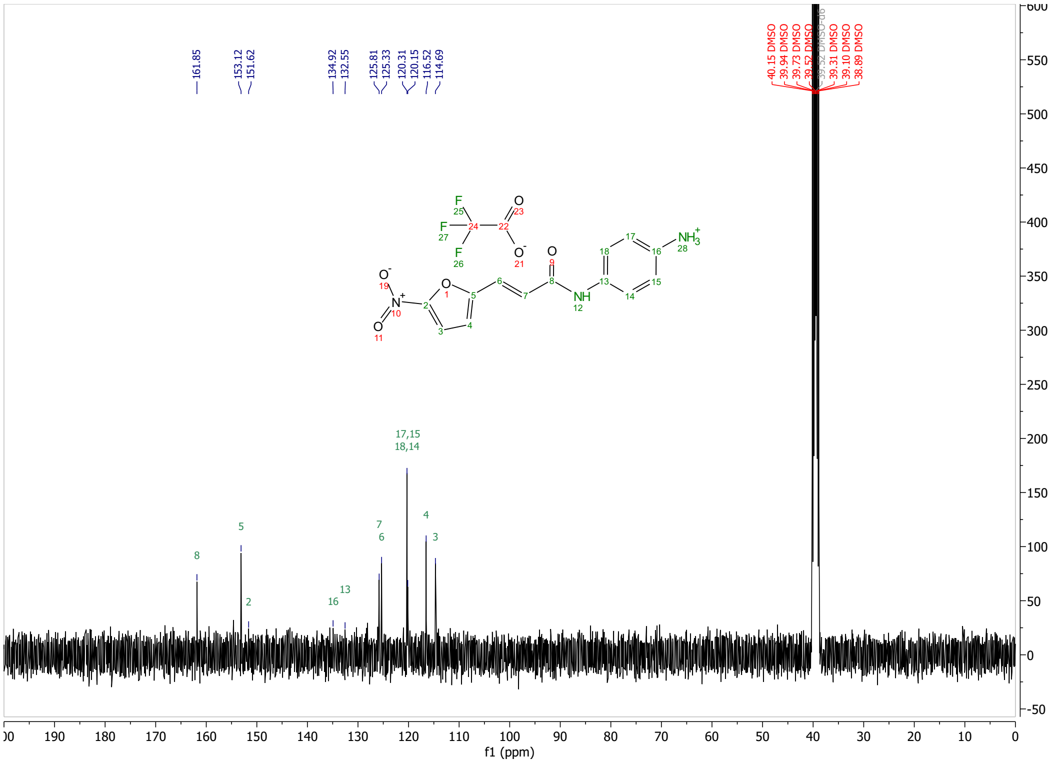

^{13}C-NMR spectrum of **D8**.

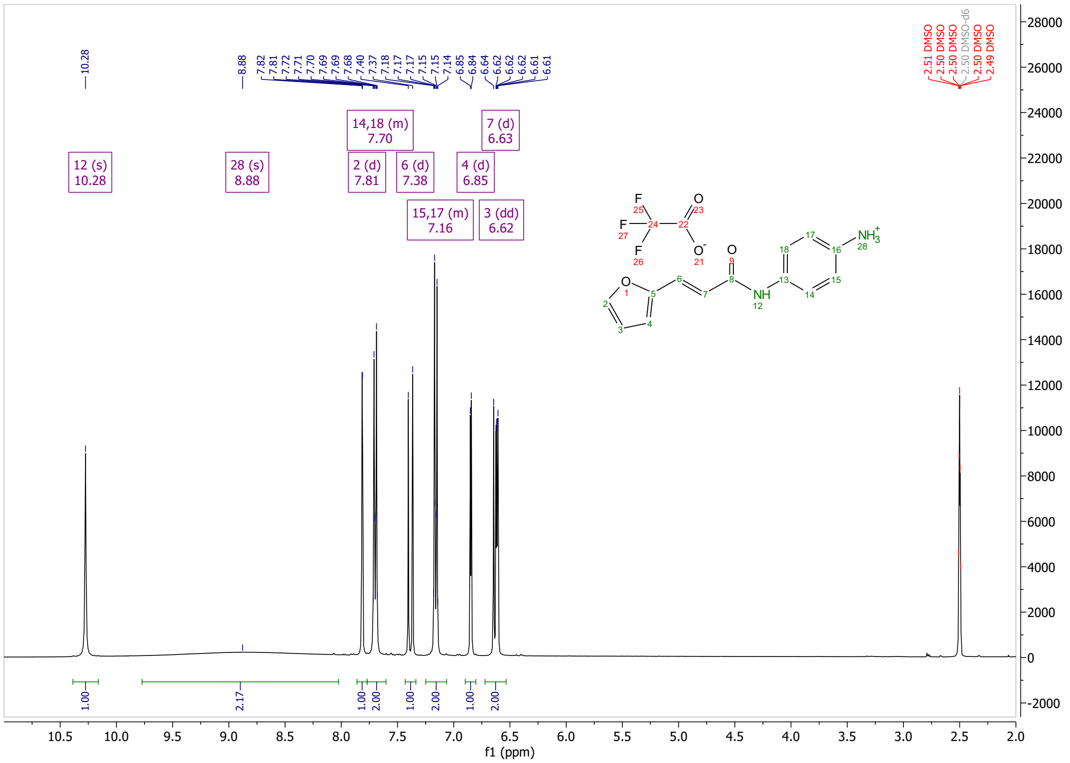

^{1}H-NMR spectrum of **D25**.

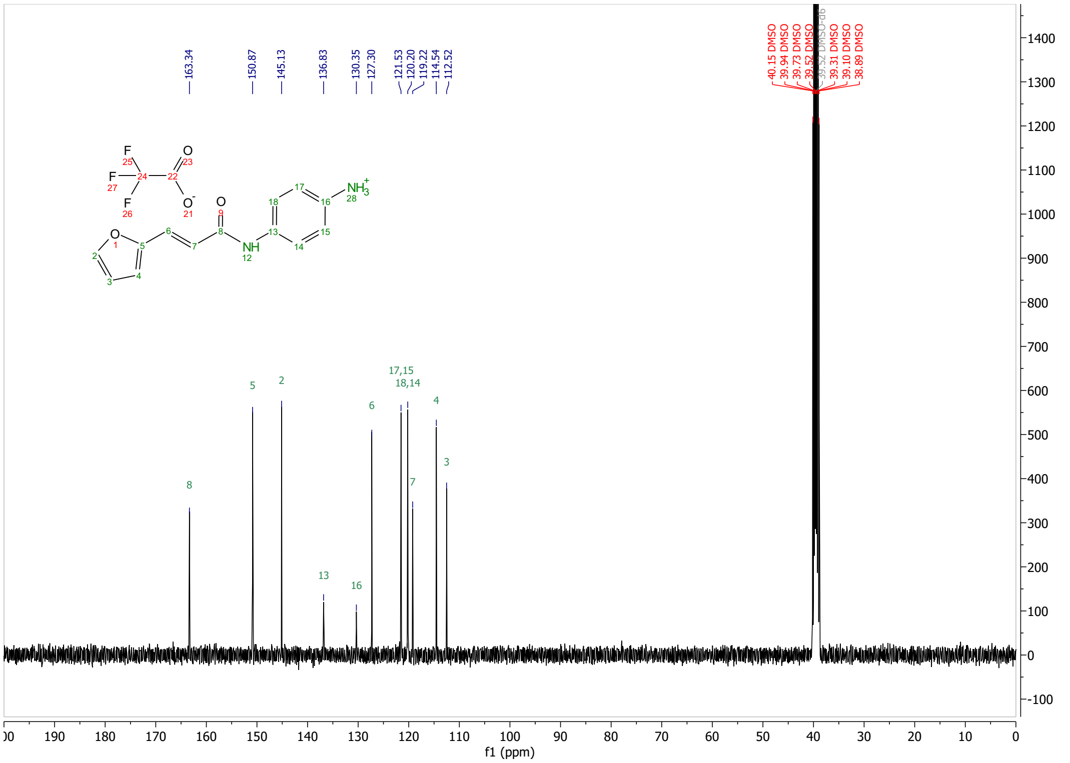

^{13}C-NMR spectrum of **D25**.

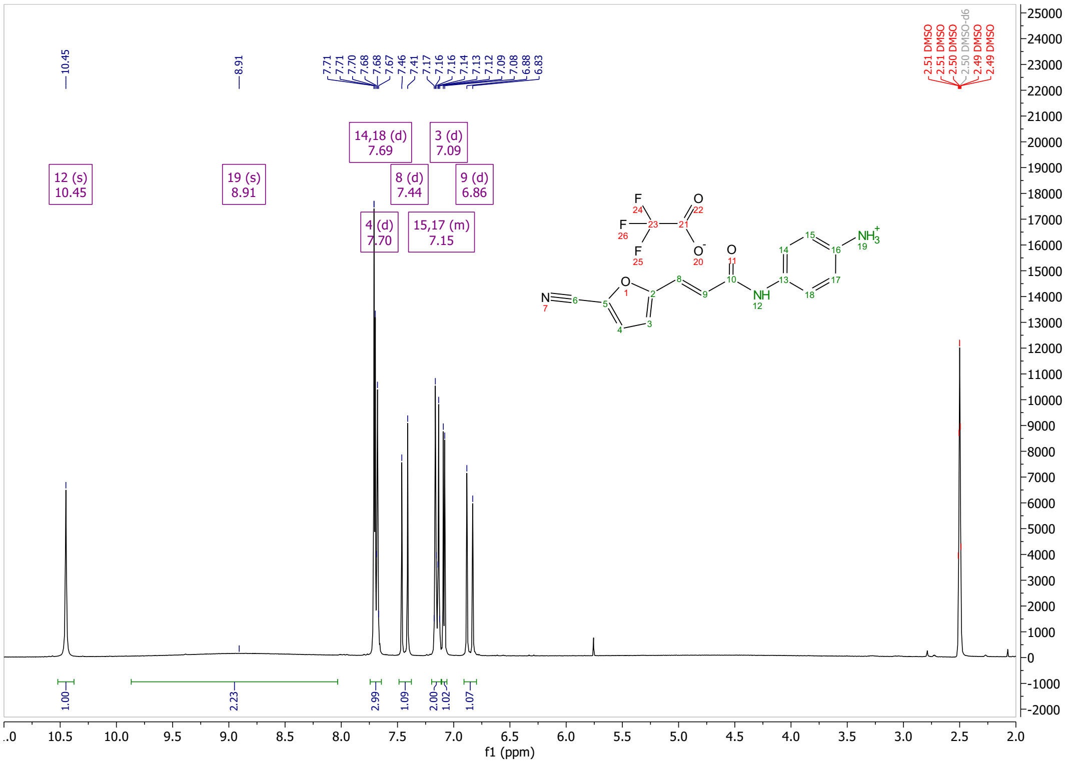

^{1}H-NMR spectrum of **D26**.

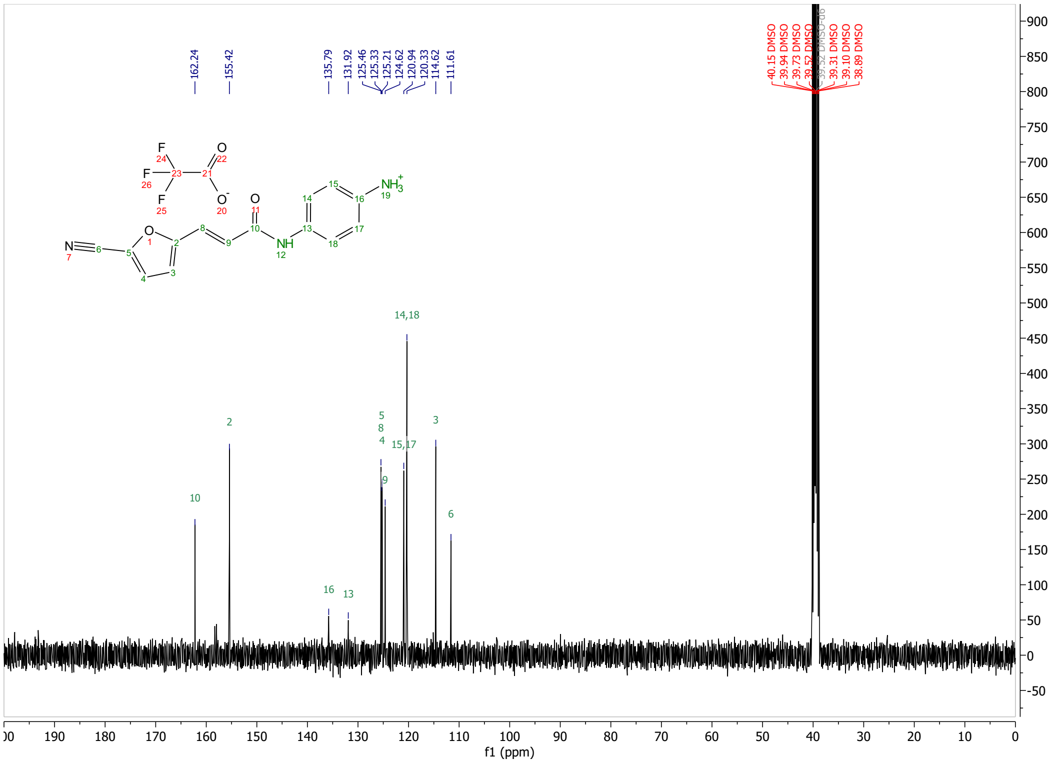

^{13}C-NMR spectrum of **D26**.

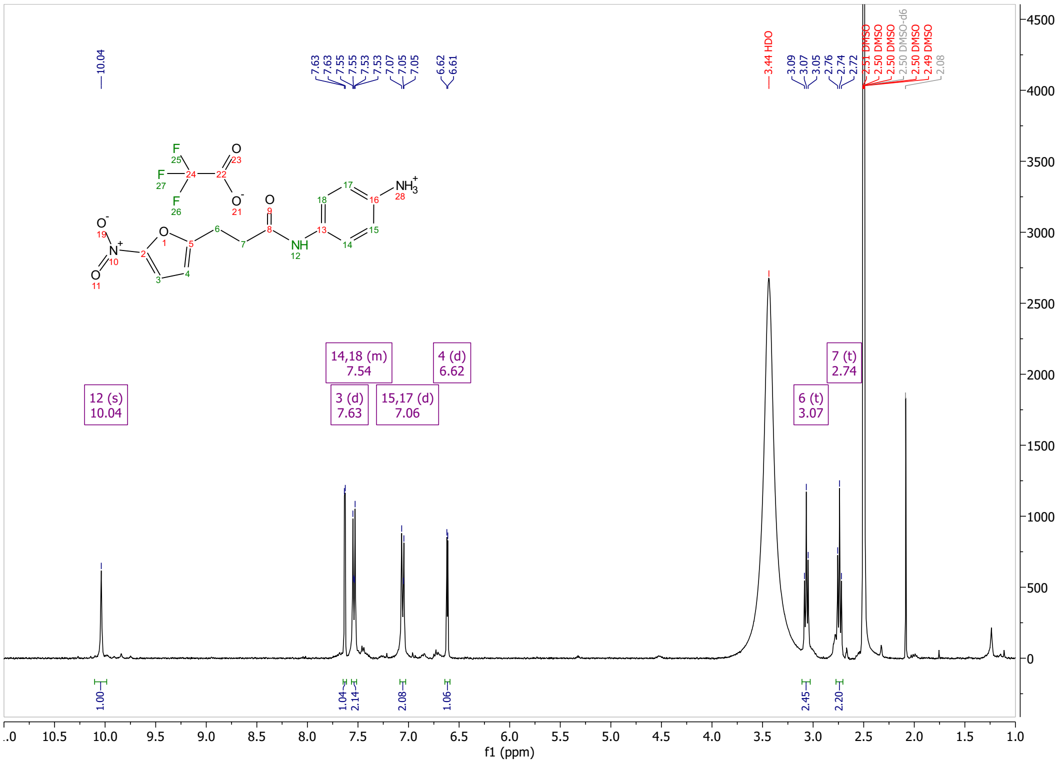

^{1}H-NMR spectrum of **D27**.

References

(S1) Schuh, M. G.; Boldini, D.; Sieber, S. A. Synergizing Chemical Structures and Bioassay Descriptions for Enhanced Molecular Property Prediction in Drug Discovery. *Journal of Chemical Information and Modeling* **2024**, *64*, 4640–4650, DOI: 10.1021/acs.jcim.4c00765.

(S2) Richter, M. F.; Drown, B. S.; Riley, A. P.; Garcia, A.; Shirai, T.; Svec, R. L.; Hergenrother, P. J. Predictive Compound Accumulation Rules Yield a Broad-Spectrum Antibiotic. *Nature* **2017**, *545*, 299–304, DOI: 10.1038/nature22308.

(S3) Preuer, K.; Renz, P.; Unterthiner, T.; Hochreiter, S.; Klambauer, G. Fréchet ChemNet Distance: A Metric for Generative Models for Molecules in Drug Discovery. *Journal of Chemical Information and Modeling* **2018**, *58*, 1736–1741, DOI: 10.1021/acs.jcim.8b00234.

(S4) Krebs, A.; Nyffeler, J.; Rahnenführer, J.; Leist, M. Normalization of data for viability and relative cell function curves. *ALTEX - Alternatives to animal experimentation* **2018**, *35*, 268–271, DOI: 10.14573/1803231.

(S5) Kim, S.; Chen, J.; Cheng, T.; Gindulyte, A.; He, J.; He, S.; Li, Q.; Shoemaker, B. A.; Thiessen, P. A.; Yu, B. et al. PubChem 2023 Update. *Nucleic Acids Research* **2023**, *51*, D1373–D1380, DOI: 10.1093/nar/gkac956.

(S6) Moret, M.; Friedrich, L.; Grisoni, F.; Merk, D.; Schneider, G. Generative Molecular Design in Low Data Regimes. *Nature Machine Intelligence* **2020**, *2*, 171–180, DOI: 10.1038/s42256-020-0160-y.

(S7) Thakkar, A.; Chadimová, V.; Bjerrum, E. J.; Engkvist, O.; Reymond, J.-L. Retrosynthetic Accessibility Score (RAscore) – Rapid Machine Learned Synthesizability Classification from AI Driven Retrosynthetic Planning. *Chemical Science* **2021**, *12*, 3339–3349, DOI: 10.1039/D0SC05401A.

(S8) Genheden, S.; Thakkar, A.; Chadimová, V.; Reymond, J.-L.; Engkvist, O.; Bjerrum, E. AiZynthFinder: A Fast, Robust and Flexible Open-Source Software for Retrosynthetic Planning. *Journal of Cheminformatics* **2020**, *12*, 1–9, DOI: 10.1186/s13321-020-00472-1.

(S9) Erickson, N.; Mueller, J.; Shirkov, A.; Zhang, H.; Larroy, P.; Li, M.; Smola, A. AutoGluon-Tabular: Robust and Accurate AutoML for Structured Data. 2020.

(S10) Landrum, G.; Tosco, P.; Kelley, B.; Ric; sriniker; gedeck; Vianello, R.; Cosgrove, D.; NadineSchneider; Kawashima, E. et al. Rdkit. Zenodo, 2023.

(S11) Ning, Y.; Tian, H.; Gui, J. Biogenesis-Guided Synthesis and Structural Revision of Sarocladione Enabled by Ruthenium-Catalyzed Endoperoxide Fragmentation. *Angewandte Chemie International Edition* **2021**, *60*, 11222–11226, DOI: https://doi.org/10.1002/anie.202101451.

(S12) Li, J.; Xie, S.; Ahmed, S.; Wang, F.; Gu, Y.; Zhang, C.; Chai, X.; Wu, Y.; Cai, J.; Cheng, G. Antimicrobial Activity and Resistance: Influencing Factors. *Frontiers in Pharmacology* **2017**, *8*, 364, DOI: 10.3389/fphar.2017.00364.

(S13) Hübner, I.; Shapiro, J. A.; Hoßmann, J.; Drechsel, J.; Hacker, S. M.; Rather, P. N.; Pieper, D. H.; Wuest, W. M.; Sieber, S. A. Broad Spectrum Antibiotic Xanthocillin X Effectively Kills Acinetobacter baumannii via Dysregulation of Heme Biosynthesis. *ACS Central Science* **2021**, *7*, 488–498, DOI: 10.1021/acscentsci.0c01621, PMID: 33791430.

(S14) Silverman, J. A.; Oliver, N.; Andrew, T.; Li, T. Resistance Studies with Daptomycin. *Antimicrobial Agents and Chemotherapy* **2001**, *45*, 1799–1802, DOI: 10.1128/aac.45.6.1799-1802.2001.

(S15) Schum, D.; Elsen, F. A. V.; Ruddell, S.; Schorpp, K.; Junca, H.; Müsken, M.; Chen, S.-Y.; Fiedler, M. K.; Pickl, T.; Pieper, D. H. et al. Screening Privileged Alkyl Guanidinium

Motifs under Host-Mimicking Conditions Reveals a Novel Antibiotic with an Unconventional Mode of Action. *4*, 3125–3134, DOI: 10.1021/jacsau.4c00449, Publisher: American Chemical Society.

(S16) Coscia, F.; Doll, S.; Bech, J. M.; Schweizer, L.; Mund, A.; Lengyel, E.; Lindebjerg, J.; Madsen, G. I.; Moreira, J. M.; Mann, M. A streamlined mass spectrometry–based proteomics workflow for large-scale FFPE tissue analysis. *251*, 100–112, DOI: 10.1002/path.5420, _eprint: https://pathsocjournals.onlinelibrary.wiley.com/doi/pdf/10.1002/path.5420.

(S17) Demichev, V.; Messner, C. B.; Vernardis, S. I.; Lilley, K. S.; Ralser, M. DIA-NN: neural networks and interference correction enable deep proteome coverage in high throughput. *17*, 41–44, DOI: 10.1038/s41592-019-0638-x, Publisher: Nature Publishing Group.

(S18) Tyanova, S.; Temu, T.; Sinitcyn, P.; Carlson, A.; Hein, M. Y.; Geiger, T.; Mann, M.; Cox, J. The Perseus computational platform for comprehensive analysis of (prote)omics data. *13*, 731–740, DOI: 10.1038/nmeth.3901, Publisher: Nature Publishing Group.
